# Development of Genome-Wide Functional Markers Using Draft Genome Assembly of Guava (*Psidium guajava* L.) cv. Allahabad Safeda to Expedite Molecular Breeding

**DOI:** 10.3389/fpls.2021.708332

**Published:** 2021-09-23

**Authors:** Sujata Thakur, Inderjit Singh Yadav, Manish Jindal, Parva Kumar Sharma, Guriqbal Singh Dhillon, Rajbir Singh Boora, Naresh Kumar Arora, Manav Indra Singh Gill, Parveen Chhuneja, Amandeep Mittal

**Affiliations:** ^1^School of Agricultural Biotechnology, Punjab Agricultural University, Ludhiana, India; ^2^Fruit Research Sub-Station, Punjab Agricultural University, Bahadurgarh, India; ^3^Department of Fruit Science, Punjab Agricultural University, Ludhiana, India

**Keywords:** *Psidium guajava* L., Allahabad Safeda, genome assembly, gene prediction and annotation, comparative transcriptomics, functional markers, genetic diversity

## Abstract

Guava (*Psidium guajava* L.), a rich source of nutrients, is an important tropical and subtropical fruit of the Myrtaceae family and exhibits magnificent diversity. Genetic diversity analysis is the first step toward the identification of parents for hybridization, genetic mapping, and molecular breeding in any crop species. A diversity analysis based on whole-genome functional markers increases the chances of identifying genetic associations with agronomically important traits. Therefore, here, we sequenced the genome of guava cv. Allahabad Safeda on an Illumina platform and generated a draft assembly of ~304 MB. The assembly of the Allahabad Safeda genome constituted >37.95% repeat sequences, gene prediction with RNA-seq data as evidence identified 14,115 genes, and BLAST n/r, Interproscan, PfamScan, BLAST2GO, and KEGG annotated 13,957 genes. A comparative protein transcript analysis of tree species revealed the close relatedness of guava with Eucalyptus. Comparative transcriptomics-based SSR/InDel/SNP-PCR ready genome-wide markers in greenish-yellow skinned and white fleshed-Allahabad Safeda to four contrasting cultivars *viz* apple-color-skinned and white-fleshed-Lalima, greenish-yellow-skinned and pink-fleshed-Punjab Pink, purple-black-skinned and purple-fleshed-Purple Local and widely used rootstock-Lucknow-49 were developed. The molecular markers developed here revealed a high level of individual heterozygosity within genotypes in 22 phenotypically diverse guava cultivars. Principal coordinate, STRUCTURE clustering, and neighbor-joining-based genetic diversity analysis identified distinct clusters associated with fruit skin and flesh color. The genome sequencing of guava, functional annotation, comparative transcriptomics-based genome-wide markers, and genetic diversity analysis will expand the knowledge of genomes of climacteric fruits, facilitating trait-based molecular breeding and diversifying the nutritional basket.

## Introduction

Guava (*Psidium guajava* L.), a member of the Myrtaceae family, is a cross-pollinated perennial fruit tree and has the chromosome number 2n = 22 (Kumar and Ranade, 1952). Except for the triploid seedless type (Raman et al., 1971), almost all of its commercial varieties are diploid. A genome size of ~495–538 MB has been reported for Brazilian white and red-fleshed diploid cultivars; however, tetraploid species such as *Psidium cattleianum* and *Psidium acutangulum*, have a larger genome size of ~1,030–1,144 MB (Da Costa et al., 2008). In India, guava is the fourth most significant fruit crop after mango, banana, and citrus (Ray, 2002). Known as the “apple of tropics” (Nakasone and Paull, 1998), guava is grown in tropical and sub-tropical regions of the world, and is a rich source of nutrients, minerals, and vitamins (Thaipong et al., 2006; Mittal et al., 2020). Despite high economic importance, genomic information regarding guava remains scarce, reducing the slope of genetic gain.

Guava fruit consumer prefers less and soft seeded, prolonged shelf life, colored flesh, apple color fruits and grower demands wilt tolerant, fruit fly resistant, high yielding and climate resilient stress tolerant cultivars. Guava exhibits variability in the color of peel and flesh, flesh thickness, seed number, distribution of seeds, seed strength, and flavor that ranges from sweet to sour in different cultivars (Mehmood et al., 2014). The guava crop is frequently threatened by guava wilt, a complex problem caused by *Fusarium sp*., nematodes, and water logging conditions, demanding the development of resistant genetic rootstocks. Fruit flies, major pests of fruit and vegetable crops, decimate the rainy season guava crop every year in the subtropics. Furthermore, the lower shelf life of guava threatens its export potential, rendering it an underutilized fruit. Natural genetic variability and selection of favorable agronomic traits *via* traditional breeding programs is a long process that relies on the arbitrary rearrangement of alleles between two closely related parent plants (Nimisha et al., 2013). Also, traditional breeding is expensive and time-consuming, particularly for fruit crops where the ontogeny cycle requires years due to the long juvenile phase (Longhi et al., 2013). These constraints can be overcome by resourceful strategies such as genomics-assisted breeding (GAB) (Varshney et al., 2005; Kole et al., 2015) or marker-assisted breeding (MAB) (Baumgartner et al., 2016), where the selection of fruit quality-related traits and resistance to biotic and abiotic stresses can be made at the seedling stage.

A tremendous surge in the availability of genomic resources in the last decade has been translated in accessing the variability in the germplasms (Jain et al., 2019). A shift from isozyme and random amplified polymorphic DNA (RAPD)-based molecular markers to gene-based simple sequence repeat (SSRs), single nucleotide polymorphisms (SNPs), and insertion-deletions (InDels) has increased the precision of gene tagging and quantitative trait locus (QTL) mapping of traits controlling fruit quality, maturity, yield, resistance to abiotic, and biotic stresses (Selmer et al., 2009; Vasemägi et al., 2010; Nimisha et al., 2013; Roorkiwal et al., 2017). SNPs (Verma et al., 2015) and InDels (Liu et al., 2013, 2015; Lv et al., 2013; Yamaki et al., 2013; Wu et al., 2014) relatively pose more abundance, can be scored with high accuracy, are highly repeatable, and are spread over the entire genome. Therefore, SNPs and InDels are preferred and have become widely used markers in the last decade for breeding programs.

The possibility of genome and transcriptome assemblies due to decrease in the cost of next-generation sequencing (NGS) has significantly advanced genomic studies progress in the last few years. NGS has led to the decoding of many genomes and enhanced the knowledge of genome architecture and the development of many molecular markers (Goodwin et al., 2016). In the last decade, many fruit crops such as grapevine (Jaillon et al., 2007), papaya (Ming et al., 2008), apple (Velasco et al., 2010), strawberry (Shulaev et al., 2011; Hirakawa et al., 2014), Japanese apricot (Zhang et al., 2012), peach (Verde et al., 2013), pear (Wu et al., 2013; Chagné et al., 2014), and mango (Wang et al., 2020) had been sequenced. In Myrtaceae species, *Eucalyptus grandis* (Myburg et al., 2014), *Leptospermum scoparium* (Thrimawithana et al., 2019), and “New Age” Chinese guava (Feng et al., 2020) have been sequenced. Here, we have sequenced the commercially important Indian guava cultivar Allahabad Safeda (AS).

Allahabad Safeda is the gold standard cultivar that has been commercially grown for ~4 decades in India owing to attractive fruit size, high total soluble sugars, vitamin C content, and better organoleptic traits. It is involved in several breeding programs at the national level. To understand the genetics of guava and boost GAB/MAB programs, we generated a draft genome assembly of AS using Illumina Paired-end reads. A comparative transcriptome analysis of AS, Purple Local (PL), CISH-G5 (Lalima), Punjab Pink (PP), and Lucknow-49 (L-49) mapped to an AS draft genome assembly developed SSR-, InDel-, and SNP-based functional markers. An evaluation of a set of 233 markers was performed to study genetic diversity in a diversity panel of 22 *P. guajava* and 2 *P*. *cattleianum* genotypes to validate the markers. The markers developed in the study would be helpful in future breeding programs for guava.

## Materials and Methods

### Allahabad Safeda Genome Sequencing and Assembly

Allahabad Safeda leaf samples were collected from a 10-year-old tree maintained in the orchards of Punjab Agricultural University, Ludhiana, in Punjab (India). The leaf samples were flash-frozen in liquid nitrogen and stored at −80°C before genomic DNA extraction. Following the instructions of the manufacturer, total DNA was extracted with DNeasy® Plant Mini-Kit (QIAGEN, Hilden, Germany). Two paired-end (PE) libraries with an insert size of 300 and 500 bp, respectively, were prepared using TruSeq® stranded DNA Library Prep (Illumina, San Diego, CA, United States) and sequenced using a HiSeq 2500 Illumina sequencer (Illumina, San Diego, CA, United States). All the raw files have been submitted to NCBI under Bioproject PRJNA557348 with Biosample SAMN12395251 under SRA SRR9865865 and SRR9865866.

The quality of PE sequence reads was checked by FastQC, v0.11.8 (Andrew 2010-http://www.bioinformatics.babraham.ac.uk/projects/fastqc). Low-quality reads were removed, and adapter sequences were trimmed using Trimmomatic, version 0.39 (Bolger et al., 2014) with parameters -ILLUMINACLIP:TruSeq3-PE.fa:2:30:10; SLIDINGWINDOW:4:15; MINLEN:50. The *de novo* assembly of the Illumina PE reads was performed by SPAdes v3.13.0 with the default settings (Bankevich et al., 2012). The draft genome assembly was further evaluated using the BBMap suite of BBTools version 36.9 (BBMap - Bushnell B. - sourceforge.net/projects/bbmap/). Benchmarking Universal Single-Copy Orthologs (BUSCO) analysis was performed to assess the completeness of the genome assembly (Simão et al., 2015). The genome assembly was deposited with NCBI as -Guava genome assembly PAU_PgAS_1 with NCBI accession VSKU00000000.

### Allahabad Safeda Gene Prediction and Genome Annotation

The *de novo* repeat identification was performed for the AS genome assembly by RepeatModeler v1.0.11 with RepeatMasker's repeat library downloaded from RepBase (*https://www.girinst.org*). RepeatMasker- version 2.1 (http://www.repeatmasker.org) was then used for complete identification of repetitive sequences, and the masked genome was further used for annotation.

Transfer RNA (tRNA) genes were predicted with tRNAscan-SE version 2.0.5 (Chan et al., 2021) using the prediction method infernal. The MAKER v 2.31.10 genome annotation pipeline (Cantarel et al., 2008) was used for gene prediction in the masked genome assembly. A Trinity-based AS transcriptome assembly (Mittal et al., 2020) was used as EST evidence. To generate *ab initio* gene predictions with the repeat masked assembly, SNAP (Korf, 2004) and AUGUSTUS v 3.2.2 (Stanke et al., 2006) were used. AUGUSTUS was trained using tomato training parameters. The MAKER pipeline was re-run with repeat, EST, SNAP hidden Markov models (hmm), and guava training parameters developed over tomato using AUGUSTUS as evidence.

All the predicted genes were annotated by comparing the protein sequences (output of MAKER—third round) against the Pfam hmm library of PfamScan (http://www.ebi.ac.uk/Tools/pfa/pfamscan). Protein domain identification was performed by comparing the protein sequences to the InterPro database using InterProScan version 5.39-77.0 (*http://www.ebi.ac.uk/interpro/download/*), with additional parameters of generating GO ids and detecting pathways. The transcript sequences of predicted genes were subjected to DIAMOND (Buchfink et al., 2014) BLAST against the n/r database of NCBI. The XML results from the DIAMOND BLAST were mapped and annotated using Blast2GO ver-5.2.5 (Conesa et al., 2005) to perform Gene Ontology functional classification of the genes, and Go ids were subsequently plotted with Web Gene Ontology Annotation Plot (WEGO2.0) (http://wego.genomics.org.cn/cgi-bin/wego/index.pl) to visualize the distribution of gene functions. Kyoto Encyclopedia of Genes and Genomes (KEGG) Automatic Annotation Server (KAAS) (Moriya et al., 2007) was used to perform the functional classification by assigning the guava protein transcript sequences to a pathway based on the KEGG database (*www.genome.jp/kegg*).

### Allahabad Safeda Phylogenetic Analysis and Generation of Pseudochromosomes

A phylogenetic analysis was conducted with Orthofinder v 2.3.12. MAKER-identified AS protein sequences were compared with that of *Musa acuminata, Ananas comosus, Oryza sativa, Zea mays, Solanum lycopersicum, Vitis vinifera, Carica papaya, Arabidopsis thaliana, Gossypium hirsutum, Populus trichocarpa, Glycine max, Cucumis sativus, Prunus persica, Malus domestica, Frageria vesca, Eucalyptus grandis, Citrus clementina*, and *Ziziphus jujube*. Protein files were downloaded from Phytozome except for *Ziziphus jujube* downloaded from https://doi.org/10.5061/dryad.83fr7. The species tree was generated with iTOL (Letunic and Bork, 2021).

Draft pseudochromosomes of AS were generated using Chromosomer (version-0.1.3) (Tamazian et al., 2016). The genome of *E. grandis* (Myburg et al., 2014) was downloaded from Phytozome and used as a reference to generate the draft chromosomes of the AS genome.

### Allahabad Safeda Genome-Wide Marker Development

#### Mining of SSRs

The MIcroSAtellite (MISA) identification tool (Beier et al., 2017) (http://pgrc.ipk-gatersleben.de/misa/misa.html) was used for the identification of microsatellites in the AS genome assembly. To design primers flanking the microsatellite loci identified with MISA, primer modeling software Primer3 (Whitehead Institute-https://sourceforge.net/projects/primer3/) was used.

#### Development of EST-SSRs/EST-InDel Markers

Ribonucleic acid sequencing for AS, PP, and Apple Color (AC) described previously (Mittal et al., 2020), PL (genotype that accumulates purple pigmentation in the foliage, flower buds, petals, and fruits at immature, mature, ripe, and over-ripe stages; Mittal et al., unpublished), and L-49 used as root stock (Mittal et al. unpublished) was performed. Trinity-based transcriptome assemblies for PP, AC, PL, and L-49 were generated as that for AS [described in (Mittal et al., 2020)]. PL, L-49, AC, and PP RNA-seq assemblies subjected to BLASTn against AS transcriptome identified polymorphic InDels. Primer3 version 0.40 (http://primer3.sourceforge.net/) was used to design the primers from positions flanking Indels/SSRs with gaps ≥8 bp and with a product size range of 80–120 bp, primer length of 20–27 bp with Tm ranging from 50 to 60°C, and GC content of 40–60%. The InDel/SSR sites were mapped to the AS genome assembly to identify the coordinates and distribution over 11 pseudochromosomes.

The designed 93 InDel and 15 SSR markers were tested for polymorphism on 24 guava genotypes. PCR products were amplified in a thermocycler with denaturation at 95°C for 3 min, 95°C for 30 s, 55°C as annealing for 30 s, and 72°C as amplification step for 1 min with a repeat of 35 cycles and a final elongation step of 5 min at 72°C in a 10-μl reaction. The reaction was performed with 7 ng DNA as a template and .5μM forward and reverse primers in GoTaq® Green Master Mix (Promega Biotech India Pvt. Ltd., New Delhi, India) in the presence of 10 mg/ml BSA (0.5 μl) and 10 mg/ml PVP (0.5μl) as adjuvants. The amplified products were resolved on 6% polyacrylamide gel containing ethidium bromide in a vertical gelelectrophoresis system (C.B.S Scientific Co., San Diego, California, USA) and visualized with an Alphaimager HP gel documentation system (ProteinSimple, San Jose, CA, United States) with the AlphaView software.

#### Development of EST-SNP Markers and KASP Assay

Bowtie2 (Langmead and Salzberg, 2012) was used for indexing the genome and mapping the RNA-seq reads of five genotypes on the AS genome. To obtain a variant call format (VCF) file that includes SNP information, the sequence alignment/map format (SAM) files were converted to binary sequence alignment/map format (BAM) files with SAMtools (Li et al., 2009) and subjected to SNP calling with Freebayes (Garrison and Marth, 2012). The high-confidence SNPs were selected with VCFtools (Danecek et al., 2011) with criteria such as Indels and SNPs above quality score > 60 and read depth >20. Polymarker (Ramirez-Gonzalez et al., 2015) was used to design the Kompetitive Allele Specific PCR (KASP) assay with the diploid genome parameter.

To confirm that the SNPs obtained were not because of heterozygosity within the genome, the RNA-seq reads of AS, PP, AC, PL, and L-49 were further screened with visualization tool Integrative Genomics Viewer (IGV) (Robinson et al., 2011) with the AS genome as a reference. SNPs that were not called because of heterozygosity within the genome and were flanked by a region of conserved 50 bp with SNP depth >25 were selected for the KASP assay. Near equidistant markers spanning the 10 pseudochromosomes were synthesized (Integrated DNA Technologies, IDT®, Coralville, IA, United States). The KASP assay was subjected to 24 genotypes in 384 well format ABI-thermocycler with PCR profile 95°C for 15 min for enzyme activation and DNA denaturation, 95°C for 20 s for subsequent DNA denaturation and 64°C for 1 min for coupled annealing and amplification as a touchdown for 10 cycles, and 57°C for 1 min for the next 30 cycles. The 4-μl reaction was set up as a 10-ng genomic DNA template, 2-μl LGC master mix (LGC Biosearch Technologies, Hoddesdon, United Kingdom), .054 μl of the primer mix [forward1 (12 μM)/forward2 (12 μM)/reverse common primer (30 μM)]. After PCR, the plate was read in an Infinite F200 Pro (Tecan, Mannesdorf, Switzerland) fluorescent reader and then assessed for polymorphism on KlusterCaller version 3.4.1.36 by the LGC Genomics software.

#### Annotation of Synthesized SSR/InDel/SNP Markers

All the synthesized primers were subjected to blastn with default parameters against the AS transcriptome. The components with full query coverage were searched for Gene Ontology (Mittal et al., 2020). Subsequently, GO ids were visualized with WEGO for binning into cellular components, biological functions, and molecular processes. All the genes harboring molecular markers mapping to the genome were analyzed for transcript expression in colored vs. non-colored tissues with edgeR (Robinson et al., 2009).

#### Genetic Diversity Analysis in Guava

Allelic information on the 22 guava genotypes (variable for flesh and peel color; Table 1) generated by SSR/InDel/SNP markers was computed with the STRUCTURE 2.3.4 software (Pritchard et al., 2000) with Burnin 250,000, Monte Carlo Markov Chain (MCMC) 750,000, and K 1–15 with 10 iterations each. The most probable K value was inferred using the DELTAk “Evanno method” (Evanno et al., 2005) using the STRUCTURE HARVESTER web (Earl and vonHoldt, 2012). Principal coordinate analysis was run for all the genotypes with GenAlEx6.5.1b2 (Peakall and Smouse, 2012). The neighbor-joining tree was constructed using MEGAX (Kumar et al., 2018). Calculations of Shannon's Information Index (*I*), effective alleles (Ne), different alleles (Na), number of loci with private alleles (PA), unbiased expected heterozygosity (uHe), the expected heterozygosity (He), and polymorphic information content (PIC) were also performed. Na was calculated by the direct count of alleles across subpopulations per loci and averaged by the arithmetic mean across loci per sub-population. Ne was calculated using He by the formula:


$$\begin{array}{l} Ne=\frac{1}{1-He}where\;He=1-\sum p^{2} \end{array}$$

**Table 1:** Popular guava genotypes/cultivars widely cultivated or used in breeding programs in India.

**S.No**.	**Genotype**	**Origin/ Pedigree**	**Remarks**
***Psidium guajava***			
**White flesh and green/pale yellow skin**			
1.	Allahabad Safeda	–	Commercial variety throughout India
2.	Lucknow-49/Sardar	Allahabad Safeda	Commercial variety throughout India (Seedling Selection)
3.	Arka Amulya	Allahabad Safeda × Seedless	Hybrid (IIHR)
4.	Shweta	Apple Color	Half sib selection (CISH)
5.	Punjab Safeda	Shweta × 17-16	Hybrid (PAU)
6.	Hisar Safeda	Allahabad Safeda × Seedless	Hybrid (CCSHAU)
7.	Seedless	Natural mutant	–
8.	VNR Bihi	Taiwan	Seedling Selection
**Pink flesh and green/pale yellow skin**			
1.	Lalit	Apple Color	Half sib-selection (CISH)
2.	Arka Kiran	Kamsari × Purple Local	Hybrid (IIHR)
3.	17-16	L-49 × Portugal	Hybrid (PAU)
4.	Punjab Pink	Portugal × L-49 = F_1_ × Apple Color	Hybrid (PAU)
5.	Punjab Kiran	Apple Color × 17-16	Hybrid (PAU)
6.	Hisar Surkha	Apple Color × Banarasi Surkha	Hybrid (CCSHAU)
7.	HB-88	Apple Color × 17-16	Hybrid (PAU)
**White flesh and apple color skin**			
1.	CISH-G1	Apple colored strains	Seedling selection (CISH)
2.	CISH-G5 (Lalima)	Apple colored strains	Seedling selection (CISH)
3.	AC1-4	Apple colored strains	Seedling selection (PAU)
4.	Punjab Apple Guava (AC 6-2)	Apple colored strains	Seedling selection (PAU)
5.	AC 10-7	Apple colored strains	Seedling selection (PAU)
**Yellow flesh and green/pale yellow skin**			
1.	Portugal	–	Selection
**Purple flesh/dark green to purple Skin**			
1.	Purple Local	Malaysia	Selection
***Psidium cattleianum*****—**Strawberry guava			
***Psidiumcattleianum var. littorale*****—**Lemon guava			

*#PAU, Punjab Agricultural University-Regional Fruit Research Station, Bahadurgarh, Punjab, India*.

*CCSHAU, Chaudhary Charan Singh Haryana Agricultural University, Hisar, Haryana, India*.

*CISH, Central Institute of Sub-Tropical Horticulture, Lucknow, Uttar Pradesh, India*.

*IIHR, Indian Institute of Horticultural Research, Bangalore, Tamil Nadu, India*.

Here, *p* is the frequency of the allele. *I* was calculated (per locus and averaged across the number of loci) using the formula:


$$\begin{array}{l} I=-\sum p_{i}\;\ln{\;p}_{i} \end{array}$$

where ln is the natural logarithm of p_i_, i.e., is frequency of ith allele, and private alleles are the alleles unique to the subpopulation. The expected heterozygosity (He) provides the probability that the two individuals would be different and was calculated using the formula:


$$\begin{array}{l} He=1-\sum p_{i}^{2} \end{array}$$

Unbiased expected heterozygosity (uHe) was calculated using the allele frequency and sample size (n) with the formula:


$$\begin{array}{l} uHe=\frac{n}{n-1}\left(1-\sum p_{i}^{2}\right) \end{array}$$

An analysis of molecular variance for white flesh, apple color, and pink flesh subgroups for within population, among population, and within an individual was performed, and the number of migrants (Nm) was calculated. The PIC value for molecular markers was calculated with PowerMarkerV3.25 (Liu and Muse, 2005).

## Results

**Figure 1:**
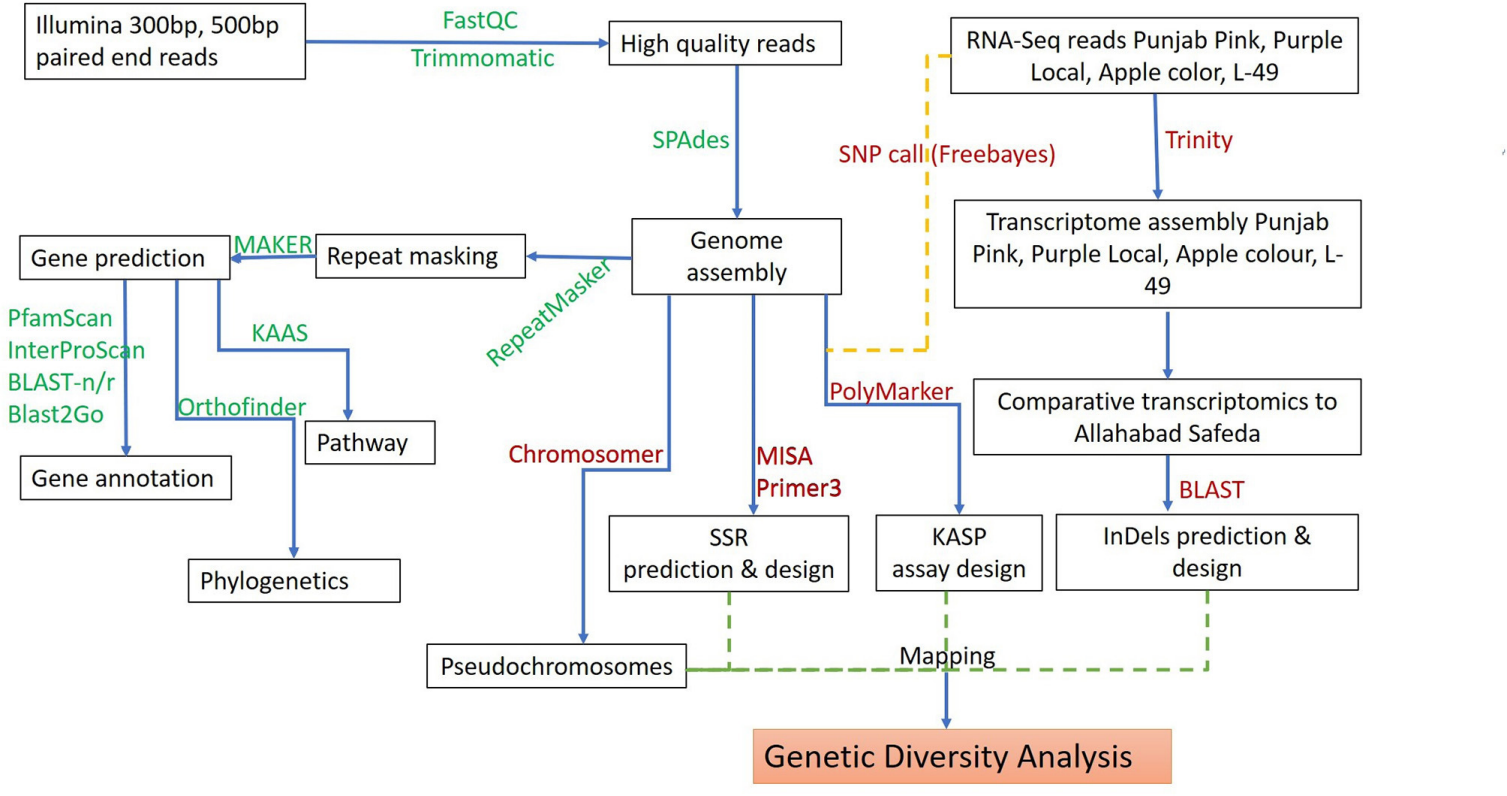
Schematic workflow for *de novo* whole-genome assembly, annotation, and development of molecular markers in guava.

The AS genomic DNA paired-end libraries sequenced on HiSeq 2500 (Illumina, San Diego, CA, United States) resulted in~22 GB raw data. The quality check with FastQC-v0.11.8 (Andrew 2010-http://www.bioinformatics.babraham.ac.uk/projects/fastqc) and trimming for adapter sequences with Trimmomatic removed low-quality reads. The surviving 92.89 and 87.02% read pairs for 300 and 500 bp-PE libraries as input for *de novo* assembly with SPAdes (default settings) led to the generation of 303.782 MB draft assembly length consisting of 45,455 scaffolds. Scaffold N_50_ of 17.992 KB with the longest scaffold of 134.502 KB represents~50X guava genome coverage (Table 2). A BUSCO analysis (using 2,121 genes of eudicotyledons) revealed that 89.9% of the genes in the guava genome were conserved with 87.3% complete and single-copy (S), 2.6% complete and duplicated (D), 5.8% fragmented (F), and only 4.3% missing (M) gene models (Figure 1, Supplementary Figure 1). Taken together, it suggests a good-quality draft genome assembly of AS for genome annotation and molecular marker development.

**Table 2:** Allahabad Safeda draft genome assembly and annotation statistics.

**Genome assembly**	**Number/Size**
Scaffolds	45,455
Scaffold N_50_ (KB)	17.992
Longest Scaffold (KB)	134.502
Number of scaffolds > 50 KB	456
Assembly Length (MB)	303.782
GC content (%)	39.51
**Genome Annotation**	
Repeat content (%)	37.95
Predicted genes	14,115
**Gene Annotation**	
InterProScan	11,686
Pfam	10,981
NCBI n/r	13,854
BLAST2GO	13,840
KAAS	5,325

### Genome Annotation of Allahabad Safeda Identifies ~38% Repeat Content and Predicted >14,000 Genes

RepeatModeler (v.1.0.10) (Smit and Hubley, 2008)-identified *de novo* repeats in the AS draft genome assembly were used as an additional repeat library, along with RepBase libraries, to find repeat elements with RepeatMasker (v.4.0.7) (http://www.repeatmasker.org). The repeats constituted > 37.95% genome assembly with 22.42% reported repetitive sequences and 15.53% unique to AS (Table 3). Both Class I long terminal repeats (LTR), (non-LTR) and Class II (DNA) repeat elements were represented in the genome. Class I retrotransposons (~12.98% repeats) were predominantly LTR elements. Interestingly, Copia and Gypsy elements in the LTR family constituted 6.71 and 3.40% known repeats, respectively. DNA elements constituted ~8.28% repeats, with MULE-MuDR accounting for 3.75% of the known repeats. We also identified 508 tRNAs in the unmasked AS draft genome and 248 in the repeat masked genome with tRNAscan-SE (Chan et al., 2021) (Supplementary Table 1). Four hundred and three tRNAs were identified as coding for 20 amino acids; however, 53 were pseudogenes. Only 29 tRNA genes exhibited the presence of introns.

**Table 3:** Allahabad Safeda draft genome assembly repeat analysis.

**Class of repeat elements**	**Length occupied (bp)**	**% age of genome assembly**
**CLASS I**		
**SINE**	563,002	0.19
**LINE**	50,95,683	1.68
L1	33,61,192	1.11
**LTR elements**	33,740,592	11.11
Copia	20,396,474	6.71
Gypsy	11139958	3.67
**CLASS II**		
**DNA elements**	25,161,969	8.28
Mule-MuDR	11,696,956	3.85
hAT-Ac	40,37,215	1.33
Helitron	220,087	0.72
Retroposon	2,226	>0
Satellites	12,084,92	0.40
smallRNA	544,227	0.18
**Unknown**	47,169,770	15.53
**Total**	115,279,351	37.95

Gene prediction in the masked draft genome assembly in the first round of MAKER identified 16,392 *ab initio* gene models with EST evidence from the previous RNA-seq of AS (Mittal et al., 2020). Using gene models computed with SNAP (hmm file), retraining parameters of *S. lycopersicon* as a training model with AUGUSTUS on the repeat masked AS genome, EST-GFF, and RM GFF, the second round of MAKER developed 9,509 trained gene models. Re-running SNAP and AUGUSTUS on second-round results of MAKER developed new hmm and guava-based retraining parameters. The re-run of MAKER (third round) with new hmm guava retraining parameters, new EST-GFF, and new RM-GFF files predicted 14,115 genes.

**Figure 2:**
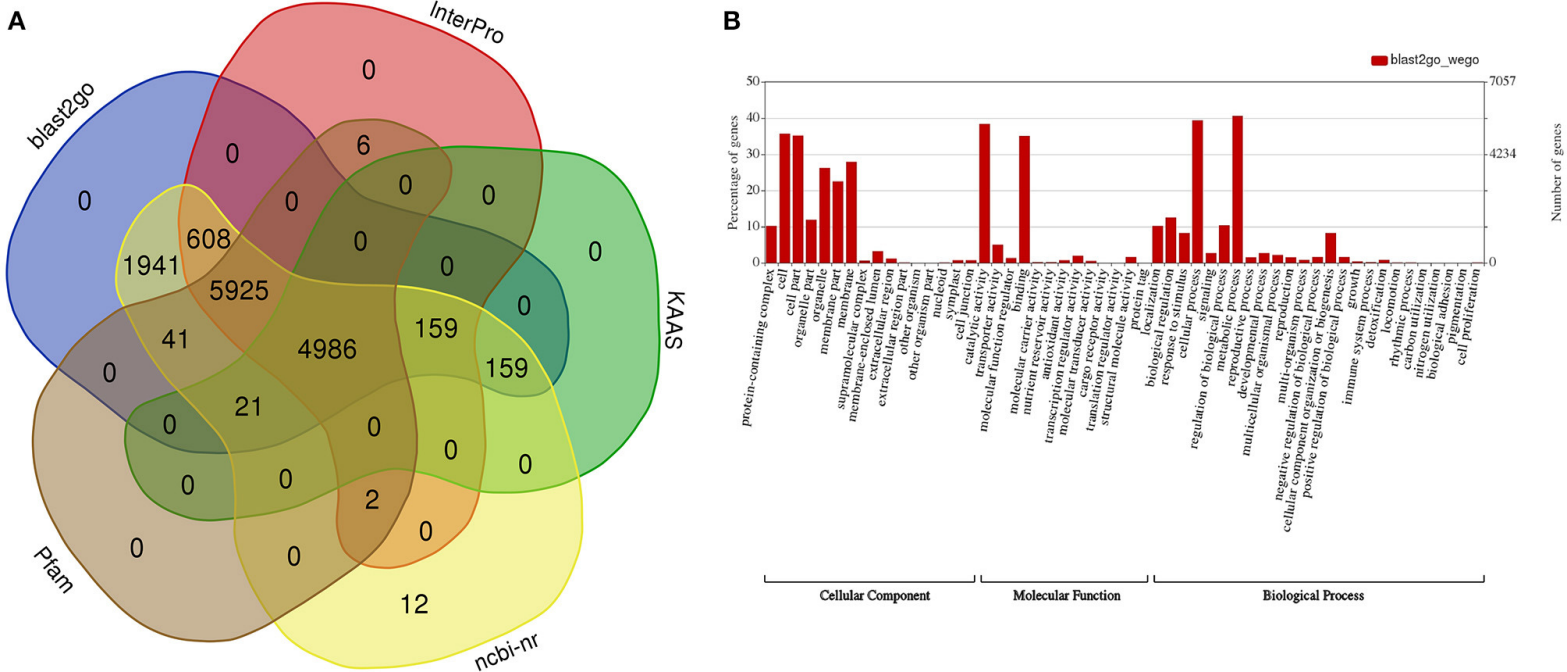
Functional annotation of predicted genes in the draft guava genome assembly. **(A)** Number of genes functionally annotated in five public databases, **(B)** functional categorization of the annotated genes into cellular components, molecular function, and biological processes with WEGO.

A functional annotation for the predicted genes discovered 11,686, 10,981 (8,534 domains, 1,746 repeats, 7,421 families, and 126 motifs), 13,854, and 13,840 genes displaying significant similarities to known proteins in the InterPro, Pfam, NCBI n/r, and GO databases, respectively (Figure 2A, Supplementary Data Files 3–6), and 13,957 (98.8%) genes were annotated in at least one of these four databases. A Gene Ontology functional classification of the predicted genes with WEGO assigned 7,766, 7,322, and 8,440 genes to the GO classes of biological process, cellular component, and molecular function, respectively (Figure 2B). A KEGG classification with KAAS assigned annotated genes to 389 pathways (Supplementary Data File 7).

### Guava Exhibits a Close Evolutionary Relationship With Eucalyptus

**Figure 3:**
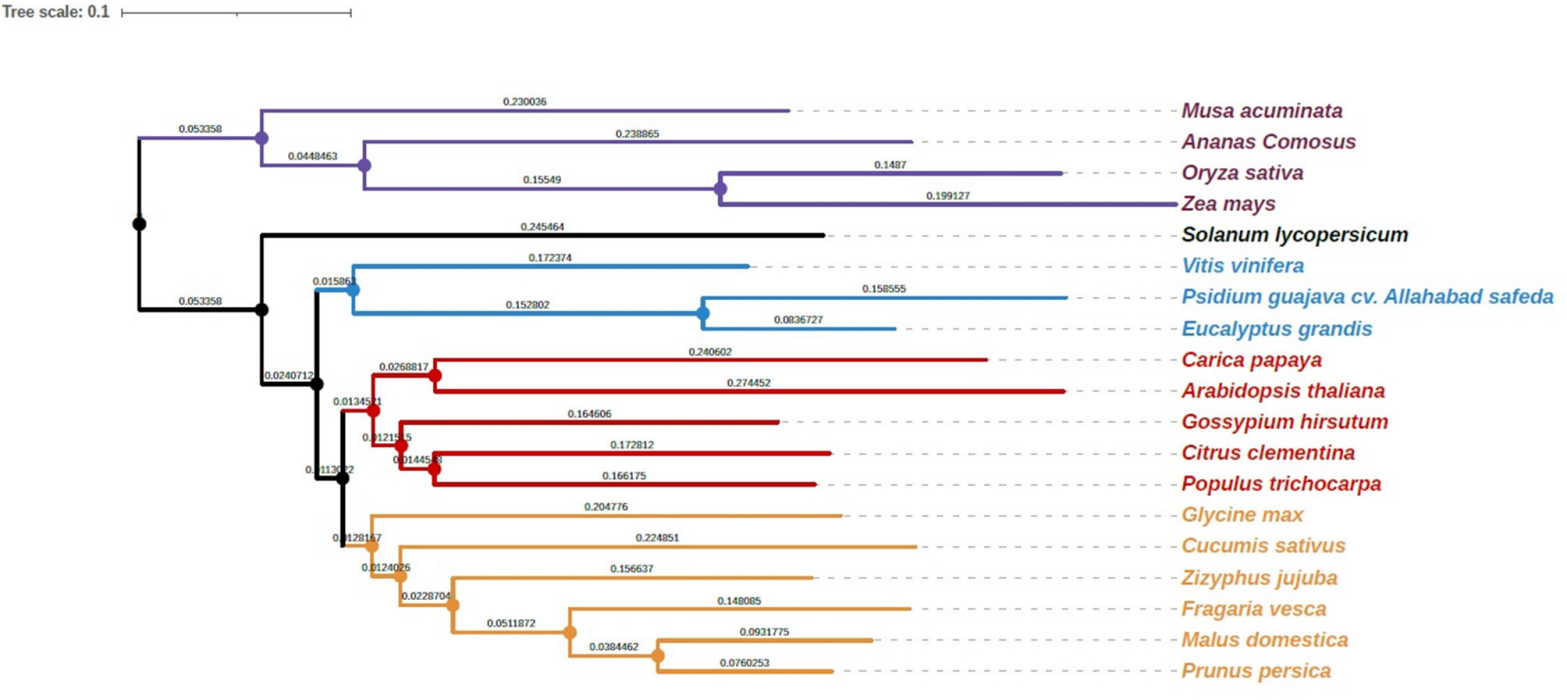
Phylogenetic analysis of guava cv. Allahabad Safeda in comparison with Myrtaceae family sequenced genome, Eucalyptus, and other sequenced fruit species. ^#^Length of the branches and scale is the same as that depicted by software.

To investigate the relationship of the guava genome with other species, we performed gene family clustering of *P. guajava* (AS), *M. acuminata* (DH-Pahang), *A. comosus* (F153), *O. sativa* (Nipponbare), *Z. mays* (PH207), *S. lycopersicum* (Heinz 1706), *V. vinifera* (PN40024), *C. papaya* (SunUp), *A. thaliana, G. hirsutum, P. trichocarpa* (Nisqually 1), *G. max* (Wm82), *C. sativus* (Gy14 gynoecious inbred line), *P. persica* (Lovell), *C. clementina* (Mandarin), *F. vesca* (Hawaii 4), *M. domestica* (Golden Delicious), *Z. jujuba* (Junzao) and *E. grandis* (non-fleshy fruit; Myrtaceae family member). OrthoFinder assigned 594,430 genes (89.6% of total) to 35,370 orthogroups (Supplementary Table 2). The rooted species tree obtained with the STAG inference of orthofinder was generated with iTOL, which placed guava adjacent to *E*. *grandis* (Figure 3).

### Fifty Percent Draft Guava Genome Maps Over 11 Chromosomes of Eucalyptus

Myrtaceae is a large family of dicotyledonous woody plants containing 130–150 genera (Grattapaglia et al., 2012), with *Eucalyptus* and *Psidium* being economically important genera. Also, owing to the close evolutionary relationship depicted by Orthofinder, we developed pseudochromosomes in guava using Eucalyptus as reference. Chromosomer (Tamazian et al., 2016) builds pseudochromosomes from genome contigs or scaffolds using alignments to the chromosomes of reference provided by a closely related species. With an average nucleotide identity of 81.411% between AS and *E. grandis*, the ~152 Mb guava genome was mapped on *E. grandis*, resulting in 11 pseudochromosomes.

### Development of Genomic-SSR and Functional SSR/InDel/SNP Markers in Guava Deploying Comparative Transcriptomics

**Figure 4:**
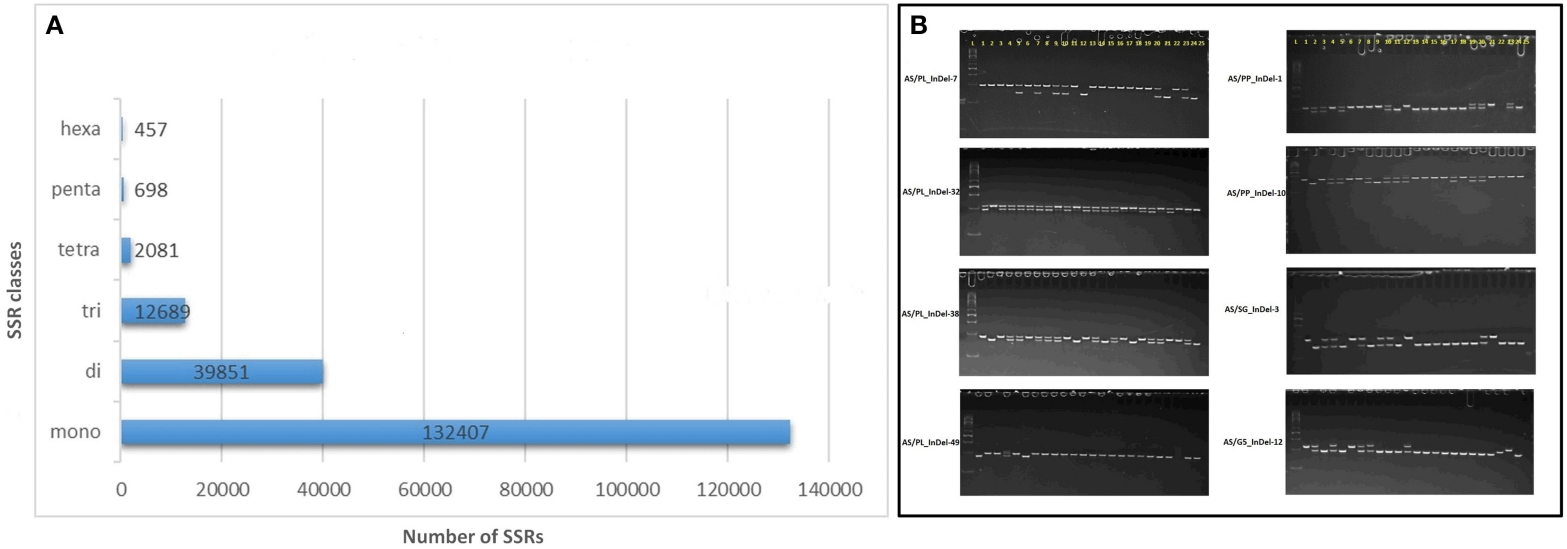
Genomic simple sequence repeat (SSR) and EST-insertion-deletion (InDel) markers in guava genome. **(A)** SSR motif distribution in guava draft genome assembly as identified with the MIcroSAtellite (MISA) identification tool. **(B)** Subset of InDel-based amplicons amplified and separated by 6% polyacrylamid gel-based electrophoresis. #InDels tested and presented represents marker developed by comparative transcriptomics of AS, Allahabad Safeda; PL, Purple Local; PP, Punjab Pink; SG/L-49, Sardar Guava/Lucknow-49; G5, Apple-Colored-CISH-G5/Lalima. L represents 50-bp DNA ladder on the left and 25 lanes represent (1) Purple Local (2) Lucknow-49/Sardar Guava (3) Shweta (4) Hisar Safeda (5) Punjab Safeda (6) VNR Bihi (7) Seedless (8) Hisar Surkha (9) Punjab Pink (10) Punjab Kiran (11) Lalit (12) Portugal (13) CISH-G5/Lalima (14) AC-62/ Punjab Apple Guava (15) AC-107 (16) AC-14 (17) Arka Kiran (18) CISH-G1 (19) HB-88 (20)17-16 (21) Lemon guava (22) Strawberry guava (23) Arka Amulya (24) Allahabad Safeda and (25) No DNA template control.

A total of 188,183 SSR loci were identified in the AS genome with the MISA script (Beier et al., 2017) and included 21,295 SSRs in compound formation (Supplementary Data File 8). Of the repeat motifs observed, mono-nucleotide repeats were the most abundant, constituting 70.3% of all the SSRs, followed by di- 21.1%, tri- 6.7%, tetra-1.1%, penta- 0.3%, and hexa- 0.02% nucleotide motifs (Figure 4A, Supplementary Data File 8). The mononucleotide repeat motifs A/T occurred at the highest frequencies (126,244) followed by C/G (6,163). Of the dinucleotide motifs, AG/CT (29,051) was the most frequent followed by AT/AT (7,509), AC/GT (3,019), and CG/CG (7,509) motifs. For the other motif, AAG/CTT (4,108), AAAG/CTTT (282), AAAAT/ATTTT (219), and AAAAAT/ATTTTT (86) were the most frequent in the trinucleotide, tetranucleotide, pentanucleotide, and hexanucleotide classes, respectively. Out of the 188,183 motifs identified, 152,367 (80.96%) SSR primers were successfully obtained with primer3_core (Supplementary Data File 9).

A set of 15 SSRs and 93 InDels was designed with Primer3 *via* AS comparative analysis with PL, PP, L-49, and AC transcripts, where primers were designed from regions flanking ≥ 8 nucleotide insertions/deletions (Supplementary Table 3). Out of the 108 primers tested, 90 (84.11%) were able to detect polymorphism in 24 genotypes (Table 1), with alleles ranging from 1 to 4 and average PIC value of 0.267. A subset of 8 such markers exhibiting biallelic polymorphism is shown in Figure 4B.

**Figure 5:**
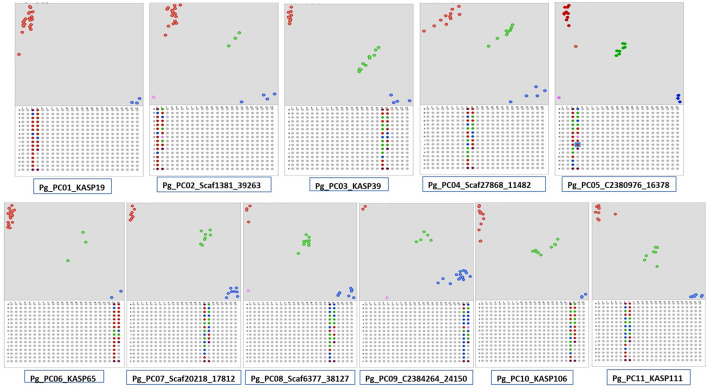
Kompetitive allele-specific polymorphism (KASP) detection assay development in guava. Single nucleotide polymorphisms (SNPs) were identified in AS/PL/L-49/AC/PP by Freebayes followed by Polymarker. Scatter plots for the selected KASP assay show clustering of genotypes on the X- (FAM) and Y- (HEX) axes on the 11 pseudochromosomes. Genotypes exhibiting blue color have a FAM-type allele, red-colored have a HEX-type allele, and green-colored have both types of alleles (heterozygotes).

A total 386,952 sequence variants were obtained with Freebayes using variant calling format files generated on PL, PP, L-49, and AC compared with AS. The variants identified included 368,556 SNPs and 18,396 InDels. After filtering with VCF tools, all the InDels were removed and based on depth (≥20 reads) and quality score (≥60), 231,632 SNPs were obtained. Among the SNPs, transitions accounted for 58% of the SNPs, where A↔G transitions were more as compared with C↔T. Transversions accounted for 41.35% of the SNPs, where T↔A transversions were the highest followed by G↔T, A↔C, and G↔C. The high-quality SNPs were used as an input with a 50-bp flanking sequence region in the Polymarker software resulting into 62,722 primer sets distributed over the guava genome (Supplementary Data File 10). The primer sets over near-equal distances spanning the 11 pseudochromosomes of guava were further screened with Integrated Genome Viewer (Supplementary Figure 2) for high-quality SNPs flanked by ≥50-bp homozygous regions. A total of 130 such high-quality near equidistant markers (Supplementary Table 4) were amplified in 24 genotypes, and 106 were found polymorphic distinguishing the genotypes, genetically. More than 61% of the markers exhibited a PIC value > 0.3. Figure 5 shows the scatter plots generated with Klustercaller by running the KASP assay with primers on all the 11 pseudochromosomes demonstrating the utility of such biallelic markers for guava molecular breeding.

**Figure 6:**
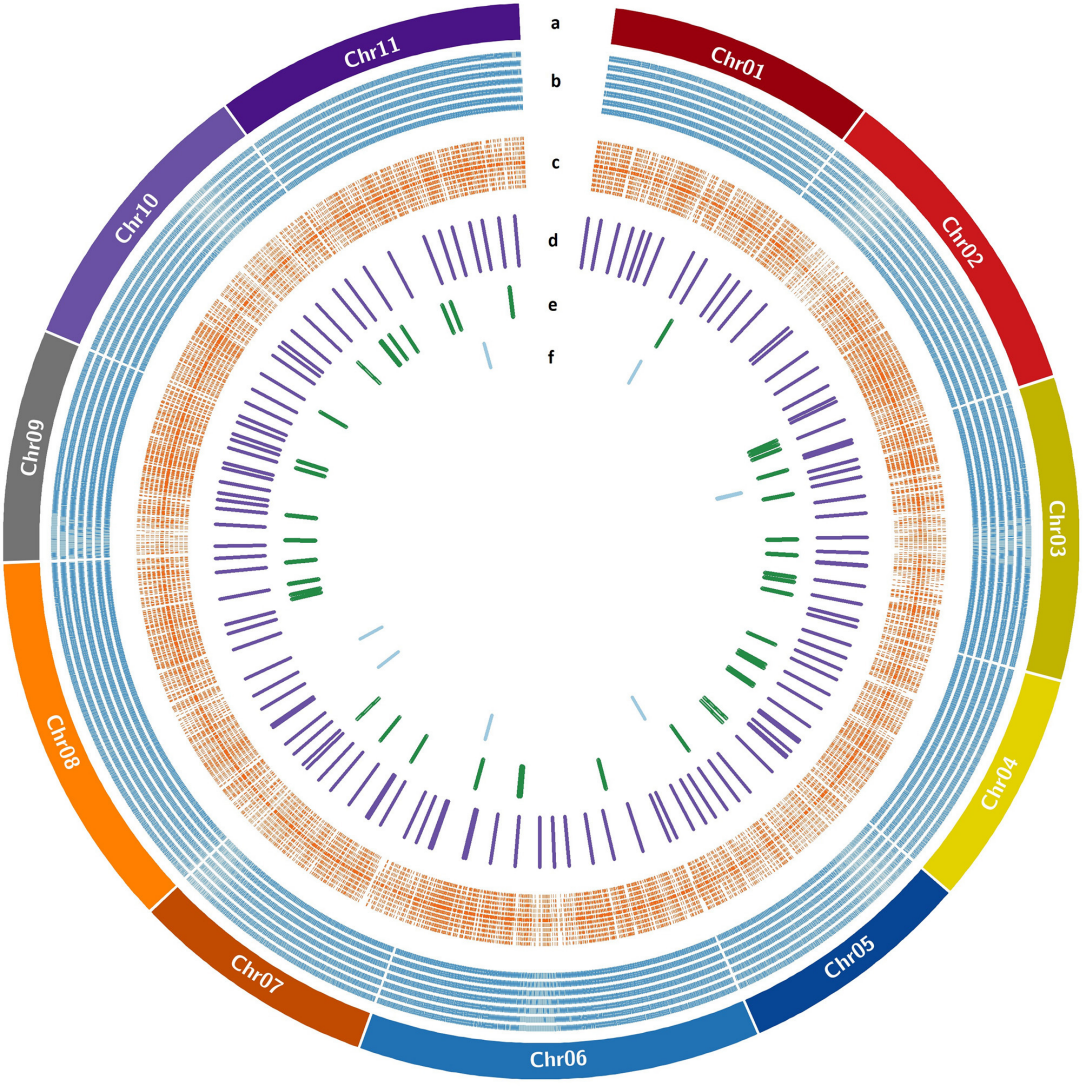
EST-SSR/InDel/SNP markers distribution over guava pseudochromosomes. **(a)** Guava pseudochromosomes, **(b)** SSR markers designed with Primer3design, **(c)** KASP markers designed with Polymarker, **(d)** validated KASP markers, **(e)** validated InDel markers, and **(f)** validated SSR markers.

**Figure 7:**
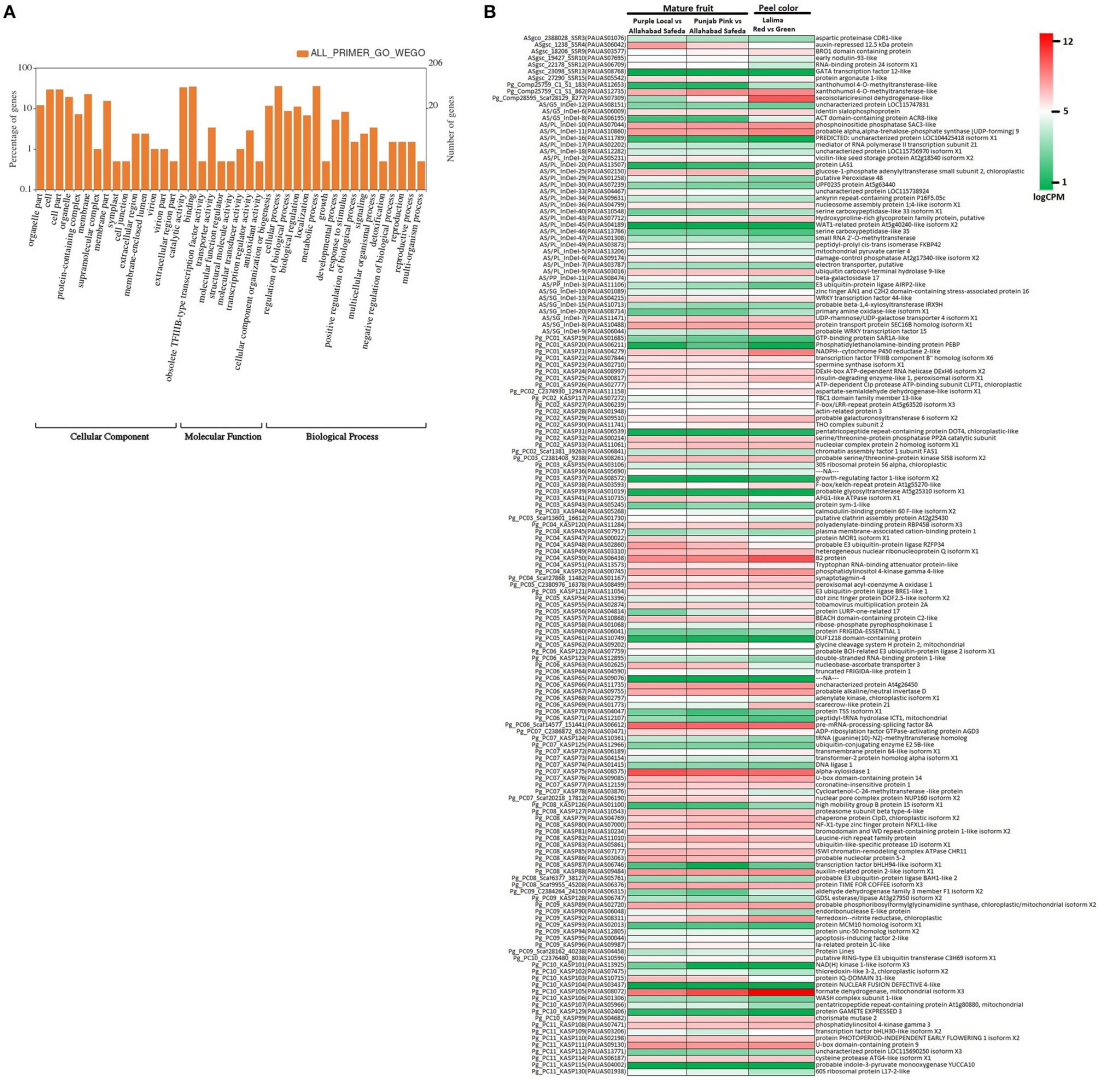
Functional classification of genes harboring markers. **(A)** WEGO analysis shows functional classification based on cellular component, molecular function, and biological process. **(B)** Heat map of gene expression in colored vs. non-colored tissues *viz*. mature fruit of Purple Local vs. Allahabad Safeda, mature fruit of Punjab Pink vs. Allahabad Safeda, and red peel vs. green peel of Lalima.

The genome wide genomic SSR and EST-InDel/SNP markers spanned over the 11 guava chromosomes are shown in Figure 6. Also, markers exhibiting polymorphism in the 24 genotypes are shown in the inner rings of the circos plot. The functional analysis of 233 tested markers with WEGO shows that 130 of them are involved in important cellular components, biological processes, or molecular functions (Figure 7A). We compared the transcript expression of a mature fruit of PL to AS, PP to AS, and red peel of Lalima to green peel. The heat map (Figure 7B) shows that the differential expression of genes in colored vs. non-colored tissues is associated with many markers.

### Newly Developed Markers Clustered Diverse Guava Genotypes/Cultivars in Accordance to Flesh/Peel Color

**Figure 8:**
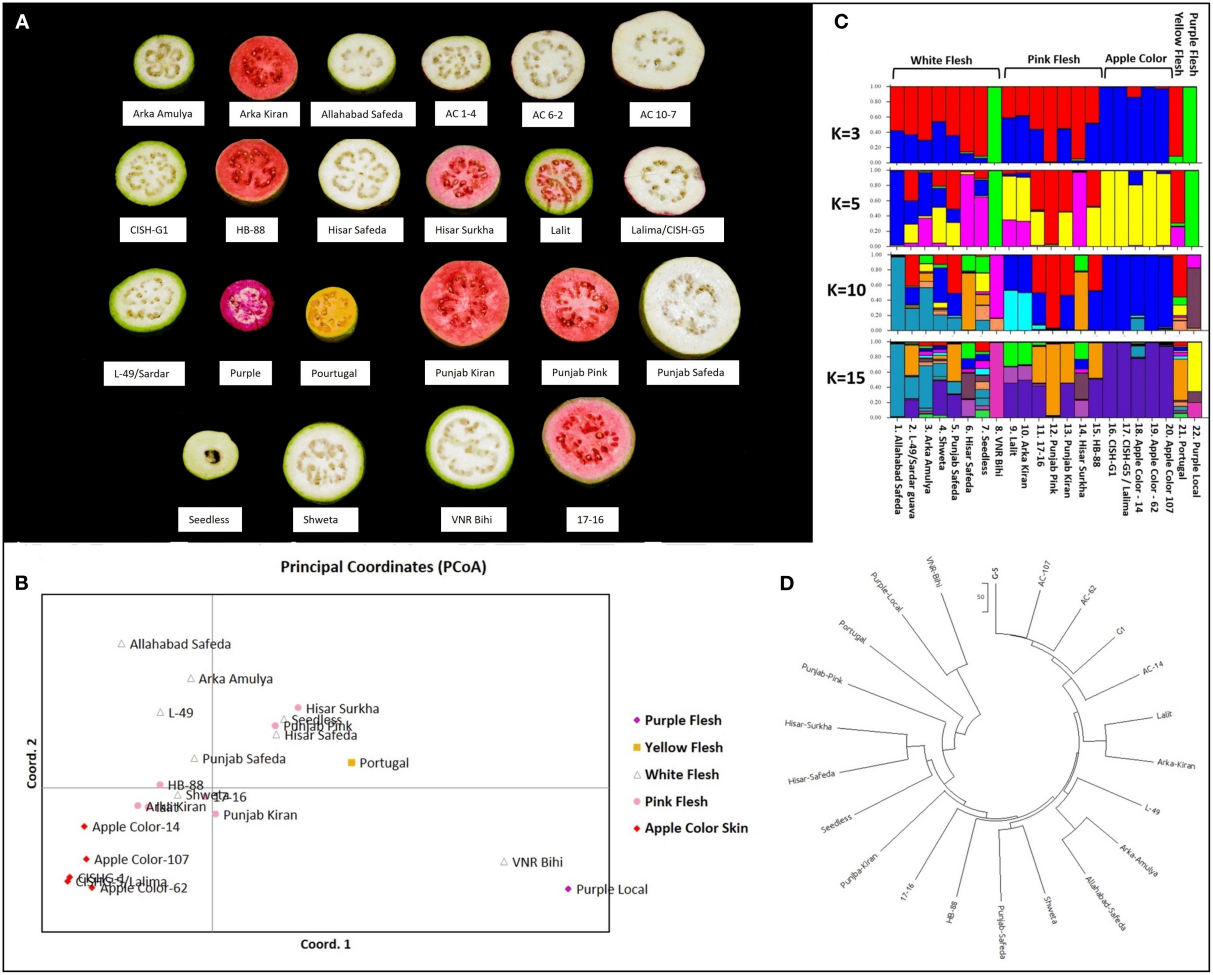
Diversity analysis of *Psidium guajava* in the 22 cultivars. **(A)** Phenotypic variability in five flesh/peel color variable cultivars. **(B)** Principal coordinate analysis computed with GenAlEx. **(C)** STRUCTURE analysis assuming admixture model with assumed structures as 3, 5, 10, and 15. **(D)** Neighbor-joining tree constructed with MEGA X (with the sum of branch length = 1451.86206055).

The principal coordinate analysis (Peakall and Smouse, 2012) was performed using the 186 validated biallelic SSR/InDel/SNP markers to investigate population clusters across 22 genotypes (Figure 8A, Table 1, Supplementary Data File 11). Accordingly, the PCoA plot indicated that the 5 fruit-flesh/skin-based genotypes generally clustered separately. The apple color peel genotypes clustered together, compared with the pink-fleshed being relatively scattered. Pink-fleshed genotypes were more closely related to the white-fleshed than the apple color, yellow, and purple-fleshed. Purple/yellow-fleshed and apple color genotypes were the most diverse and did not show overlap (Figure 8B).

The population structure of the 22 genotypes was inferred using the clustering program STRUCTURE, testing for 1 to 15 clusters (K) (Figure 8C). Evanno's correction revealed the peak of delta K at K = 3, suggesting the presence of three main clusters (Supplementary Figure 3), and an additional peak at K = 6 suggests the presence of a substructure. Based on the optimal K = 3 by STRUCTURE, cluster 1 consisted of four pink-fleshed (PP, Hisar Surkha, Punjab Kiran and 17–16), a yellow-fleshed (Portugal), and six white fleshed genotypes (Hisar Safeda, Arka Amulya, Punjab Safeda, L-49, Seedless, and AS). Cluster 2 consisted of PL and VNR Bihi, and cluster 3 consisted of all apple color genotypes, Shweta, and the remaining pink-fleshed genotypes (Supplementary Figure 4).

At K = 5, cluster 1 divided into three separate clusters where one cluster had three pink- (PP, Punjab Kiran, and 17-16), two white- (Punjab Safeda, L-49), and a yellow fleshed genotype (which remained the same even at K = 10). Another cluster had AS and Arka Amulya clustered together, and the last cluster had Hisar Surkha, Hisar Safeda, and Seedless (Supplementary Figure 4). At K = 10, all the apple-colored genotypes formed one cluster; two white-fleshed (Shweta, Seedless), two pink- (Lalit and Arka Kiran), and two white-fleshed (AS and Arka Amulya) formed three clusters separately, while Seedless displayed maximum admixture (Supplementary Figure 4). At K = 15 the trend of clusters remained the same. However, Portugal, Arka Amulya, and Shweta also displayed a high level of admixture. Interestingly, the apple-colored genotypes were never assigned to a new independent cluster as K was increased. The results of STRUCTURE were in coherence with the results of the PCoA analyses. The neighbor-joining tree constructed with MEGAX (Kumar et al., 2018) was also consistent with the results of the PCoA and STRUCTURE analyses (Figure 8D). In conclusion, the analysis with three independent methods supports the division of these diverse genotypes correlated with fruit color; however, pink- and white fleshed ones show an overlap.

Furthermore, we examined the allelic patterns of the subgroups and found them highly diverse. The mean Shannon's information index (I), the expected heterozygosity (He), and the unbiased expected heterozygosity (uHe) were high (0.289, 0.198, and 0.255, respectively), suggesting a high level of diversity among and within the genotypes (Table 4). The highest diversity was shown by the subgroup consisting of white flesh color with I, He, and uHe of 0.506, 0.342, and 0.365, respectively. Interestingly, despite the average Na, Ne, and PA of 1.499, 1.345, and 0.12 in the 22 *P. guajava* cultivars, the highest Na, Ne, and PA (1.909, 1.594, and 0.011) was observed for the subgroup with white flesh color. With 11 unique loci contributing to private alleles, eight genotypes across four out of five subgroups carried private alleles (Table 5). With six being the highest number of private alleles for PL, these might be linked with the unique characteristics of the genotype (Mittal et al. Unpublished). The analysis of molecular variance (AMOVA) showed that out of the total variation in the subgroups white flesh, apple color, and pink flesh, only 12% variation was among the subgroups and 19% variation was among the individuals, while most of the variation was within each individual (69%), depicting high-level heterozygosity of the examined loci (Table 6). The Nm was low at 1.905, depicting a low number of gene migrations among the subgroups (Table 6). Also, the PCoA graph exhibited very less overlap among the subgroups (Figure 8B). A low number of gene migrations indicate that the subgroups have evolved in distinct environments with very little interaction for gene migration *via* crossing.

**Table 4:** Allelic pattern diversity indexes for each subgroup based on the number of different alleles (Na), number of effective alleles (Ne), Shannon's information index (*I*), private alleles frequency (PA), expected heterozygosity (He), and unbiased expected heterozygosity (uHe).

**Sub-group**	**Purple Flesh**	**Yellow Flesh**	**White Flesh**	**Pink Flesh**	**Apple Color**	**Mean**
Na	1.070 ± 0.023	1.344 ± 0.037	1.909 ± 0.021	1.823 ± 0.028	1.349 ± 0.035	1.499
Ne	1.070 ± 0.023	1.344 ± 0.037	1.594 ± 0.024	1.525 ± 0.025	1.194 ± 0.023	1.345
I	0.060 ± 0.014	0.250 ± 0.024	0.506 ± 0.015	0.454 ± 0.018	0.178 ± 0.019	0.289
PA	0.032 ± 0.013	0.011 ± 0.008	0.011 ± 0.008	0.000 ± 0.000	0.005 ± 0.005	0.012
He	0.043 ± 0.010	0.180 ± 0.018	0.342 ± 0.011	0.306 ± 0.013	0.117 ± 0.013	0.198
uHe	0.086 ± 0.021	0.360 ± 0.035	0.365 ± 0.012	0.330 ± 0.014	0.131 ± 0.014	0.255

*± Represents standard error*.

**Table 5:** Distribution of private alleles in the 22 *Psidium guajava* genotypes.

**Genotype**	**Sub-population**	**No. PA**	**Loci with Private Alleles**
Purple Local	Purple	6	Pg_PC06_Scaf14577_151441, Pg_Comp25759_C1_S1_862, Pg_PC01_KASP24, Pg_PC10_KASP99, AS/PL_InDel-20, AS/PL_InDel-46
Portugal	Yellow Flesh	2	AS/SG_InDel-18, ASgsc_5291_SSR5
Thailand	White Flesh	2	Pg_PC03_C2381408_9238, AS/PL_InDel-40
Seedless	White Flesh	1	AS/PL_InDel-40
Arka Amulya	White Flesh	1	AS/PL_InDel-40
Allahabad Safeda	White Flesh	2	Pg_PC03_C2381408_9238, AS/PL_InDel-40
CISH-G5/Lalima	Apple Color	1	AS/G5_InDel-4
Apple Color-107	Apple Color	1	AS/G5_InDel-4

**Table 6:** Analysis of molecular variance for white flesh, apple color, and pink flesh subgroups for within population, among population, and within individual variation.

**Source**	**df^#^**	**SS^#^**	**MS^#^**	**Est. Var.^#^**	**%^#^**	**Nm^#^**
Among sub-population	2	173.536	86.768	3.881	12%	1.905
Among individuals	17	610.864	35.933	6.367	19%	
Within individuals	20	464.000	23.200	23.200	69%	
Total	39	1248.400		33.447	100%	

# *K, K value for sub-populations; source, source of variation; df, degree of freedom; SS, sum of squares; MS, mean sum of squares; Est. Var., estimated variation; %, percentage of variance explained; and Nm, haploid number of migrants*.

## Discussion

### Allahabad Safeda Evolutionary Relationship

A draft genome of heterozygous *Psidium guajava* cultivar AS has been successfully assembled by Illumina-based NGS at ~50x coverage. Owing to the scarce genomic resources in tropical and subtropical fruit species as compared with temperate families such as Vitaceae, Rosaceae, and Rutaceae, the genome assembly of guava provides a foundation for evolutionary studies, comparative genomic investigation of the unique biological characteristic, intraspecific genome diversity, and molecular breeding in this nutraceutical rich crop. Repeat content in guava (37.95%) is at par with the repeat content of smaller genomes of similar sizes such as 35% in rice (Matsumoto et al., 2005), 40.5% in mango (Wang et al., 2020), 41.4% in grapevine (Jaillon et al., 2007), 44.5% in Eucalyptus (Myburg et al., 2014), and 45% in *Citrus clementina* as compared with the larger genomes that consist almost entirely of repetitive sequences such as wheat and maize with a repeat content of ~85% (Schnable et al., 2009; Appels et al., 2018). Therefore, the guava genome provides additional evidence that there is a linear increase in the repeat-sequence content of the genome with genome size (Novák et al., 2020).

Comparing predicted proteins in AS with 18 other plant species demonstrates close evolutionary relatedness between *E*. *grandis* and *P*. *guajava*. All the monocots *M. acuminata, A. comosus, Z. mays* and *O. sativa* grouped together, while the Rosaceae species *P. persica, M. domestica*, and *F*. *vesca* clustered separately. The precision of comparative genome analysis depends on deciphering proteomes as to how their genomes were assembled and annotated (Chagné et al., 2014). In AS, we identified 764 species-specific genes that did not have orthologs detected in the other species (Supplementary Table 2). Further analysis of these proteins using a more comprehensive array of species for comparison would be required to determine whether these proteins encode for traits specific to guava.

### Guava Expressed Genome-Based Markers—Leveraging Targeted Molecular Breeding

Owing to the long juvenility period and difficulty in developing standard mapping populations like that in cereals, pulses, and other annuals, we need a large number of molecular markers to make MAS possible in fruit tree species. In guava RAPD, ISSR, AFLP, COS (Prakash et al., 2002; Risterucci et al., 2005; Rai et al., 2012), and, very recently, genomic SSR markers (Kumar et al., 2020) have been developed and used for germplasm characterization and genetic diversity analysis. Under the aegis of the European Union Project “GUAVAMAP,” SSR markers for *P*. *guajava* were developed with a traditional approach involving the construction of an SSR-enriched library followed by cloning and Sanger sequencing. The first molecular linkage map was developed with AFLP and COS library based on MADS-, HOMEO-box, and RGL sequence-derived markers in a bi-parental F_1_population (MP1) of a cross “Enana Cubana roja × N6” (Valdés-Infante et al., 2003).

Next-generation sequencing tools have equipped the researchers with precise technologies for marker development, thus evading laborious methods that involve the construction of genomic and cDNA libraries followed by cloning and sequencing. The annotation of coding sequences and NGS-based EST comparison among colored vs. white flesh, apple color vs. green skin color, soft seed vs. hard seed, and short vs. long shelf life should lead to the generation of EST-SSR and EST-SNP makers. Such markers may directly be associated with the traits of interest (Varshney et al., 2005). However, in the absence of genome assembly, the generation of markers for targeted breeding is difficult. Therefore, the ultimate goal of the assembled genome here is to serve as a guideline in developing tools for MAB in guava. We identified an abundant number of SSRs and SNPs, and structural variations such as InDels spread across the whole genome, which will be highly useful in developing functional markers for guava breeding. MYB-based (Takos et al., 2006; Ban et al., 2007; Chagné et al., 2016) and red TE-based specific markers (Zhang et al., 2019) developed to distinguish the red skin from non-red in apple; SNP-based markers for Alternaria brown spot in citrus (Cuenca et al., 2016), markers for citrus Tristeza virus resistance, and for demarcating polyembryonyin citrus (Gentile et al., 2020) are several utilized examples emphasizing the necessity of marker development for the pre-selection of hybrid seedlings for commercially important traits. Here, we developed high throughput genomic SSR markers from genome assembly (Supplementary Data File 9). The Validation of 15 SSRs on the 24 genotypes promises their utilization for genetic mapping, as already shown independently (Kumar et al., 2020). The comparative transcriptomic-based InDel and KASP assay markers on a genome-wide basis make this study the first report on the development of function-based markers in guava. Out of the 233 markers tested, 195 were able to detect polymorphism in the 24 genotypes studied. The biallelic markers exhibit a PIC value of 0–0.5 (Botstein et al., 1980). In the case of the authors, they identified a high average PIC value of 0.279, emphasizing the high potential utility of the newly developed markers for genetic diversity analysis. Moreover, 130 of these polymorphic markers were involved in cellular, biological, and molecular functions. This novel set of SSR/InDel/SNP-based markers ensures expedited molecular breeding programs in guava. Furthermore, the identification of >60,000 KASP assay-ready SNPs in phenotypically diverse genotypes (Supplementary Data File 10) provides a gold mine for developing a plethora of genome-wide biallelic markers, the heart of targeted association/bi-parental mapping.

### Guava Population Structure and Diversity of Indian Cultivars

Population structure analysis is often the primary step for understanding genetic diversity, performing genome-wide association mapping to identify true marker-trait associations, and identifying genes associated with traits (Luo et al., 2019). The genetic diversity and population structure of guava genotypes in India are underexplored, so germplasm characterization should be instrumental in selecting diverse parental genotypes for varietal improvement (Kumar et al., 2020). SSR markers developed (Risterucci et al., 2005) under the aegis of GUAVAMAP have been utilized for characterization and genetic diversity analysis of guava germplasm worldwide (Viji et al., 2010; Priya et al., 2011; Coser et al., 2012; Sitther et al., 2014; Kherwar et al., 2018; Ma et al., 2020). Recently developed 26 g-SSRs (Kumar et al., 2020) were also used to estimate genetic diversity. However, 186 novel EST-based InDels and SNP markers for population structure analysis in guava is an important step forward for functional marker utilization in this fruit crop. These EST-markers clustered distinctly the apple-colored, purple-fleshed, yellow-fleshed, white-fleshed, and pink-fleshed genotypes (Figure 8). The NJ tree placed all the apple-colored genotypes next to each other, and white fleshed AS and Arka Amulya together with L-49, explaining the fact that L-49 is a chance seedling from AS. The genotype 17-16 is the female parent of Punjab Kiran and HB88, and the clustering showed all three in a single group. Hisar Safeda and Hisar Surkha grouped together explaining the fact that other than the difference in flesh color, the tree habit, foliage, fruit shape is so similar that it is hard to differentiate between two cultivars without cutting open the fruit. A similar genetic inference has also been derived independently (Kumar et al., 2020). Most of the pink-fleshed and white-fleshed genotypes showed dispersed clusters rather than clustering independently, raising the demand for a larger number of markers on a genome-wide scale. However, it might even be more complicated like Hisar Safeda and Hisar Surkha that pink-fleshed and white-fleshed genotypes are genetically similar (Kherwar et al., 2018; Kumar et al., 2020).

Private alleles provide important information on identifying distinctive genetic variability at loci and diversified genotypes, which could be employed in crop breeding to enhance allele affluence in a population (Borba et al., 2009; Salem and Sallam, 2016). The calculation of private alleles reveals the allelic information of a certain predefined subpopulation. The six loci bearing the private alleles in PL signify that these loci are critical for explaining the phenotypic diversity of this unique cultivar. Thus, studying the genes in the genomic regions bearing these loci is important to establish the role of these genes in providing unique phenotypic qualities to PL.

Overall, the draft genome, gene information linked to biological and other resources such as high-throughput and EST-based InDel/SNP markers, developed in the this study provides crucial information on the genus *Psidium* as well as the Myrtaceae family and lays the ground for improvement of quality and agronomic traits by gene mapping in biparental populations for fruit quality, biotic and abiotic stresses, genome-wide association, and comparative genomics. The draft genome provided here can be used as a reference to re-sequence the *Psidium* germplasm for mining candidate gene-based high-throughput markers and developing SNP arrays for guava breeders. The availability of such information would make genomic selection possible for guava breeding programs.

## Data Availability Statement

Data has been submitted to NCBI under Bio project PRJNA557348, Biosample SAMN12395251 under SRA SRR9865865, SRR9865866, and genome assembly PAU_PgAS_1 with NCBI accession VSKU00000000. All data and materials generated or analyzed during this study are included in this article or are available from the corresponding author on reasonable request. The Guava Genome Annotation & Markers are available at https://doi.org/10.6084/m9.figshare.14573262.v1.

## Author Contributions

AM and IY conceived the idea. AM, MJ, ST, NA, and RB collected the plant material. IY, ST, PS, and AM performed the bioinformatics analysis. ST, MJ, and AM performed the wet lab experiments. NA, RB, and MG maintained the orchards, provided the plant material, and coordinated the phenotypic analysis. PC, GD, and ST conducted KASP marker development and genetic analysis. ST and AM wrote the paper. AM, IY, PC, and MG coordinated the overall experimentation. All authors read the manuscript and approved it.

## Funding

The experiments were supported by the self-financing scheme of Punjab Agricultural University, Ludhiana and Department of Biotechnology Grant No: BT/PR24373/AGIII/103/1012/2018 to AM.

## Conflict of Interest

The authors declare that the research was conducted in the absence of any commercial or financial relationships that could be construed as a potential conflict of interest.

## Publisher's Note

All claims expressed in this article are solely those of the authors and do not necessarily represent those of their affiliated organizations, or those of the publisher, the editors and the reviewers. Any product that may be evaluated in this article, or claim that may be made by its manufacturer, is not guaranteed or endorsed by the publisher.

## Supplementary Material

Figshare for the excel sheet as Data files can be found online at: https://doi.org/10.6084/m9.figshare.14573262.v1

## Abbreviations

AS: Allahabad Safeda
NGS: next generation sequencing
SNPs: single nucleotide polymorphisms
InDel: insertion/deletion
SSR: simple sequence repeat
MAB: marker-assisted breeding
GAB: genomics-assisted breeding
BLAST: Basic Local Alignment Search Tool
KASP: Kompetitive Allele-Specific PCR
PAGE: polyacrylamid gel electrophoresis
KEGG: Kyoto Encyclopedia of Genes and Genomes
BUSCO: Benchmarking Universal Single-Copy Orthologs
PCoA: principal coordinate analysis
QTLs: quantitative trait loci
RAPD: random amplified polymorphic DNA.

## References

[B1] Appels R. Eversole K. Feuillet C. Keller B. Rogers J. Stein N.. (2018). Shifting the limits in wheat research and breeding using a fully annotated reference genome. Science 361:eaar7191. 10.1126/science.aar7191 30115783

[B2] Ban Y. Honda C. Hatsuyama Y. Igarashi M. Bessho H. Moriguchi T. (2007). Isolation and functional analysis of a MYB transcription factor gene that is a key regulator for the development of red coloration in apple skin. Plant Cell Physiol. 48, 958–970. 10.1093/pcp/pcm066 17526919

[B3] Bankevich A. Nurk S. Antipov D. Gurevich A. A. Dvorkin M. Kulikov A. S.. (2012). SPAdes: a new genome assembly algorithm and its applications to single-cell sequencing. J. Comput. Biol. 19, 455–477. 10.1089/cmb.2012.0021 22506599 PMC3342519

[B4] Baumgartner I. O. Kellerhals M. Costa F. Dondini L. Pagliarani G. Gregori R.. (2016). Development of SNP-based assays for disease resistance and fruit quality traits in apple (Malus × domestica Borkh.) and validation in breeding pilot studies. Tree Genet. Genomes 12:35. 10.1007/s11295-016-0994-y

[B5] Beier S. Thiel T. Münch T. Scholz U. Mascher M. (2017). MISA-web: a web server for microsatellite prediction. Bioinformatics 33, 2583–2585. 10.1093/bioinformatics/btx198 28398459 PMC5870701

[B6] Bolger A. M. Lohse M. Usadel B. (2014). Trimmomatic: a flexible trimmer for Illumina sequence data. Bioinformatics 30, 2114–2120. 10.1093/bioinformatics/btu170 24695404 PMC4103590

[B7] Borba T. C. de O. Mendes C. dos A. Guimarães É. P. Brunes T. O. Fonseca J. R.. (2009). Genetic variability of Brazilian rice landraces determined by SSR markers. Pesqui. Agropecuária Bras. 44, 706–712. 10.1590/S0100-204X2009000700009

[B8] Botstein D. White R. L. Skolnick M. Davis R. W. (1980). Construction of a genetic linkage map in man using restriction fragment length polymorphisms. Am. J. Hum. Genet. 32, 314–331. 6247908 PMC1686077

[B9] Buchfink B. Xie C. Huson D. H. (2014). Fast and sensitive protein alignment using DIAMOND. Nat. Methods 12, 59–60. 10.1038/nmeth.3176 25402007

[B10] Cantarel B. L. Korf I. Robb S. M. C. Parra G. Ross E. Moore B.. (2008). MAKER: an easy-to-use annotation pipeline designed for emerging model organism genomes. Genome Res. 18, 188–196. 10.1101/gr.6743907 18025269 PMC2134774

[B11] Chagné D. Crowhurst R. N. Pindo M. Thrimawithana A. Deng C. Ireland H.. (2014). The draft genome sequence of European pear (*Pyrus communis* L. 'Bartlett'). PLoS ONE 9:e92644. 10.1371/journal.pone.0092644 24699266 PMC3974708

[B12] Chagné D. Kirk C. How N. Whitworth C. Fontic C. Reig G.. (2016). A functional genetic marker for apple red skin coloration across different environments. Tree Genet. Genomes 12. 10.1007/s11295-016-1025-8

[B13] Chan P. Lin B. Mak A. Lowe T. (2021). tRNAscan-SE 2.0: improved detection and functional classification of transfer RNA Genes. Nucleic Acids Res. 10.1093/nar/gkab688 34417604 PMC8450103

[B14] Conesa A. Götz S. García-Gómez J. M. Terol J. Talón M. Robles M. (2005). Blast2GO: a universal tool for annotation, visualization and analysis in functional genomics research. Bioinformatics 21, 3674–3676. 10.1093/bioinformatics/bti610 16081474

[B15] Coser S. M. Ferreira M. F. da S. Ferreira A. Mitre L. K. Carvalho C. R.. (2012). Assessment of genetic diversity in *Psidium guajava* L. using different approaches. Sci. Hortic. 148, 223–229. 10.1016/j.scienta.2012.09.030

[B16] Cuenca J. Aleza P. Garcia-Lor A. Ollitrault P. Navarro L. (2016). Fine mapping for identification of citrus alternaria brown spot candidate resistance genes and development of new SNP markers for marker-assisted selection. Front. Plant Sci. 7:1948. 10.3389/fpls.2016.01948 28066498 PMC5179576

[B17] Da Costa I. R. Dornelas M. C. Forni-Martins E. R. (2008). Nuclear genome size variation in fleshy-fruited Neotropical Myrtaceae. Plant Syst. Evol. 276, 209–217. 10.1007/s00606-008-0088-x

[B18] Danecek P. Auton A. Abecasis G. Albers C. A. Banks E. DePristo M. A.. (2011). The variant call format and VCFtools. Bioinformatics 27, 2156–2158. 21653522 10.1093/bioinformatics/btr330 PMC3137218

[B19] Earl D. A. vonHoldt B. M. (2012). STRUCTURE HARVESTER: a website and program for visualizing STRUCTURE output and implementing the Evanno method. Conserv. Genet. Resour. 4, 359–361. 10.1007/s12686-011-9548-7

[B20] Evanno G. Regnaut S. Goudet J. (2005). Detecting the number of clusters of individuals using the software STRUCTURE: a simulation study. Mol. Ecol. 14, 2611–2620. 10.1111/j.1365-294X.2005.02553.x 15969739

[B21] Feng C. Feng C. Lin X. Liu S. Li Y. Kang M. (2020). A chromosome-level genome assembly provides insights into ascorbic acid accumulation and fruit softening in guava (*Psidium guajava*). Plant Biotechnol. J. 19, 717–730. 10.1111/pbi.13498 33098334 PMC8051600

[B22] Garrison E. Marth G. (2012). Haplotype-based variant detection from short-read sequencing. arXiv 1–9. *arXIV: 1207.3907*.

[B23] Gentile A. Malfa L. S. Deng Z. (2020). The Citrus Genome. Cham: Springer.

[B24] Goodwin S. McPherson J. D. McCombie W. R. (2016). Coming of age: ten years of next-generation sequencing technologies. Nat. Rev. Genet. 17, 333–351. 10.1038/nrg.2016.49 27184599 PMC10373632

[B25] Grattapaglia D. Vaillancourt R. E. Shepherd M. Thumma B. R. Foley W. Külheim C.. (2012). Progress in Myrtaceae genetics and genomics: eucalyptus as the pivotal genus. Tree Genet. Genomes 8, 463–508. 10.1007/s11295-012-0491-x

[B26] Hirakawa H. Shirasawa K. Kosugi S. Tashiro K. Nakayama S. Yamada M.. (2014). Dissection of the octoploid strawberry genome by deep sequencing of the genomes of fragaria species. DNA Res. 21, 169–181. 10.1093/dnares/dst049 24282021 PMC3989489

[B27] Jaillon O. Aury J. M. Noel B. Policriti A. Clepet C. Casagrande A.. (2007). The grapevine genome sequence suggests ancestral hexaploidization in major angiosperm phyla. Nature 449, 463–467. 10.1038/nature06148 17721507

[B28] Jain A. Roorkiwal M. Kale S. Garg V. Yadala R. Varshney R. K. (2019). InDel markers: an extended marker resource for molecular breeding in chickpea. PLoS ONE 14:e0213999. 10.1371/journal.pone.0213999 30883592 PMC6422259

[B29] Kherwar D. Usha K. Mithra S. V. A. Singh B. (2018). Microsatellite (SSR) marker assisted assessment of population structure and genetic diversity for morpho-physiological traits in guava (*Psidium guajava* L.). J. Plant Biochem. Biotechnol. 27, 284–292. 10.1007/s13562-017-0438-2

[B30] Kole C. Muthamilarasan M. Henry R. Edwards D. Sharma R. Abberton M.. (2015). Application of genomics-assisted breeding for generation of climate resilient crops: progress and prospects. Front. Plant Sci. 6:563. 10.3389/fpls.2015.00563 26322050 PMC4531421

[B31] Korf I. (2004). Gene finding in novel genomes. BMC Bioinformatics 5:59. 10.1186/1471-2105-5-59 15144565 PMC421630

[B32] Kumar C. Kumar R. Singh S. K. Goswami A. K. Nagaraja A. Paliwal R.. (2020). Development of novel g-SSR markers in guava (Psidium guajava L.) cv. Allahabad Safeda and their application in genetic diversity, population structure and cross species transferability studies. PLoS ONE 15:e0237538. 10.1371/journal.pone.0237538 32804981 PMC7431106

[B33] Kumar L. S. S. Ranade S. G. (1952). Autotriploidy in guava (Psidium guajava, Linn.). Curr. Sci. 21, 75–76. Available online at: jstor.org/stable/24212422

[B34] Kumar S. Stecher G. Li M. Knyaz C. Tamura K. (2018). MEGA X: molecular evolutionary genetics analysis across computing platforms. Mol. Biol. Evol. 35, 1547–1549. 10.1093/molbev/msy096 29722887 PMC5967553

[B35] Langmead B. Salzberg S. L. (2012). Fast gapped-read alignment with Bowtie 2. Nat. Methods 9, 357–359. 10.1038/nmeth.1923 22388286 PMC3322381

[B36] Letunic I. Bork P. (2021). Interactive Tree Of Life (iTOL) v5: an online tool for phylogenetic tree display and annotation. Nucleic Acids Res. 49, 293–296. 10.1093/nar/gkab301 33885785 PMC8265157

[B37] Li H. Handsaker B. Wysoker A. Fennell T. Ruan J. Homer N.. (2009). The Sequence Alignment/Map format and SAMtools. Bioinformatics 25, 2078–2079. 10.1093/bioinformatics/btp352 19505943 PMC2723002

[B38] Liu B. Wang Y. Zhai W. Deng J. Wang H. Cui Y.. (2013). Development of InDel markers for *Brassica rapa* based on whole-genome re-sequencing. Theor. Appl. Genet. 126, 231–239. 10.1007/s00122-012-1976-6 22972202

[B39] Liu K. Muse S. V. (2005). PowerMaker: an integrated analysis environment for genetic maker analysis. Bioinformatics 21, 2128–2129. 10.1093/bioinformatics/bti282 15705655

[B40] Liu L. Dang P. M. Chen C. Y. (2015). Development and utilization of InDel markers to identify peanut (*Arachis hypogaea*) disease resistance. Front. Plant Sci. 6:988. 10.3389/fpls.2015.00988 26617627 PMC4643128

[B41] Longhi S. Cappellin L. Guerra W. Costa F. (2013). Validation of a functional molecular marker suitable for marker-assisted breeding for fruit texture in apple (Malus × domestica Borkh.). Mol. Breed. 32, 841–852. 10.1007/s11032-013-9912-2

[B42] Luo Z. Brock J. Dyer J. M. Kutchan T. Schachtman D. Augustin M.. (2019). Genetic diversity and population structure of a *Camelina sativa* spring panel. Front. Plant Sci. 10:184. 10.3389/fpls.2019.00184 30842785 PMC6391347

[B43] Lv H. hao Yang L. mei Kang J. gen Wang Q. biao Wang X. wu Fang Z.. (2013). Development of InDel markers linked to Fusarium wilt resistance in cabbage. Mol. Breed. 32, 961–967. 10.1007/s11032-013-9925-x

[B44] Ma Z. Liu S. Liang Z. Xu S. Hu W. (2020). Analysis of genetic diversity of 45 guava germplasm evaluated using SSR markers. Int. J. Fruit Sci. 20, 385–393. 10.1080/15538362.2019.1640168

[B45] Matsumoto T. Wu J. Kanamori H. Katayose Y. Fujisawa M. Namiki N.. (2005). The map-based sequence of the rice genome. Nature 436, 793–800. 10.1038/nature03895 16100779

[B46] Mehmood A. Jaskani M. J. Khan I. A. Ahmad S. Ahmad R. Luo S.. (2014). Genetic diversity of Pakistani guava (*Psidium guajava* L.) germplasm and its implications for conservation and breeding. Sci. Hortic. 172, 221–232. 10.1016/j.scienta.2014.04.005

[B47] Ming R. Hou S. Feng Y. Yu Q. Dionne-Laporte A. Saw J. H.. (2008). The draft genome of the transgenic tropical fruit tree papaya (*Carica papaya* Linnaeus). Nature 452, 991–996. 10.1038/nature06856 18432245 PMC2836516

[B48] Mittal A. Yadav I. S. Arora N. K. Boora R. S. Mittal M. Kaur P.. (2020). RNA-sequencing based gene expression landscape of guava cv. Allahabad Safeda and comparative analysis to colored cultivars. BMC Genomics 21:484. 10.1186/s12864-020-06883-6 32669108 PMC7364479

[B49] Moriya Y. Itoh M. Okuda S. Yoshizawa A. C. Kanehisa M. (2007). KAAS: an automatic genome annotation and pathway reconstruction server. Nucleic Acids Res. 35, 182–185. 10.1093/nar/gkm321 17526522 PMC1933193

[B50] Myburg A. A. Grattapaglia D. Tuskan G. A. Hellsten U. Hayes R. D. Grimwood J.. (2014). The genome of *Eucalyptus grandis*. Nature 510, 356–362. 10.1038/nature13308 24919147

[B51] Nakasone H. Y. Paull R. E. (1998). Tropical Fruits. Wallingford: Cab International.

[B52] Nimisha S. Kherwar D. Ajay K. M. Singh B. Usha K. (2013). Molecular breeding to improve guava (*Psidium guajava* L.): current status and future prospective. Sci. Hortic. 164, 578–588. 10.1016/j.scienta.2013.10.017

[B53] Novák P. Guignard M. S. Neumann P. Kelly L. J. Mlinarec J. KoblíŽková A.. (2020). Repeat-sequence turnover shifts fundamentally in species with large genomes. Nat. Plants 6, 1325–1329. 10.1038/s41477-020-00785-x 33077876

[B54] Peakall R. Smouse P. E. (2012). GenALEx 6.5: genetic analysis in Excel. Population genetic software for teaching and research-an update. Bioinformatics 28, 2537–2539. 10.1093/bioinformatics/bts460 22820204 PMC3463245

[B55] Prakash D. P. Narayanaswamy P. Sondur S. N. (2002). Analysis of molecular diversity in guava using RAPD markers. J. Hortic. Sci. Biotechnol. 77, 287–293. 10.1080/14620316.2002.11511494

[B56] Pritchard J. K. Stephens M. Donnelly P. (2000). Inference of population structure using multilocus genotype data. Genetics 155, 945–959. 10.1093/genetics/155.2.945 10835412 PMC1461096

[B57] Priya K. Latha P. M. Aswath C. Reddy L. Padmakar B. Vasugi C.. (2011). Cultivar identification and genetic fingerprinting of guava (*Psidium guajava*) using microsatellite markers. Int. J. Fruit Sci. 11, 184–196. 10.1080/15538362.2011.578521

[B58] Rai M. K. Phulwaria M. Harish Gupta A. K. Shekhawat N. S. Jaiswal U. (2012). Genetic homogeneity of guava plants derived from somatic embryogenesis using SSR and ISSR markers. Plant Cell. Tissue Organ Cult. 111, 259–264. 10.1007/s11240-012-0190-1

[B59] Raman V. S. Rangasamy S. R. S. Manimekalai G. (1971). Triploidy and seedlessness in guava (*Psidium guajava* L.). Cytologia 36, 392–399. 10.1508/cytologia.36.392

[B60] Ramirez-Gonzalez R. H. Uauy C. Caccamo M. (2015). PolyMarker: a fast polyploid primer design pipeline. Bioinformatics 31, 2038–2039. 10.1093/bioinformatics/btv069 25649618 PMC4765872

[B61] Ray P. K. (2002). Breeding Tropical and Subtropical Fruits. New Delhi: Springer Science and Business Media.

[B62] Risterucci A. M. Duval M. F. Rohde W. Billotte N. (2005). Isolation and characterization of microsatellite loci from Psidium guajava L. Mol. Ecol. Notes 5, 745–748. 10.1111/j.1471-8286.2005.01050.x

[B63] Robinson J. T. Thorvaldsdóttir H. Winckler W. Guttman M. Lander E. S. Getz G.. (2011). Integrative genomics viewer. Nat. Biotechnol. 29, 24–26. 10.1038/nbt.1754 21221095 PMC3346182

[B64] Robinson M. D. McCarthy D. J. Smyth G. K. (2009). edgeR: a Bioconductor package for differential expression analysis of digital gene expression data. Bioinformatics 26, 139–140. 10.1093/bioinformatics/btp616 19910308 PMC2796818

[B65] Roorkiwal M. Jain A. Thudi M. Varshney R. K. (2017) Advances in chickpea genomic resources for accelerating the crop improvement, in The Chickpea Genome. Compendium of Plant Genomes, eds Varshney R. Thudi M. Muehlbauer F. (Cham: Springer). 10.1007/978-3-319-66117-9_6

[B66] Salem K. F. M. Sallam A. (2016). Analysis of population structure and genetic diversity of Egyptian and exotic rice (*Oryza sativa* L.) genotypes. Comptes Rendus Biol. 339, 1–9. 10.1016/j.crvi.2015.11.003 26727453

[B67] Schnable P. S. Ware D. Fulton R. S. Stein J. C. Wei F. Pasternak S.. (2009). The B73 maize genome: Complexity, diversity, and dynamics. Science 326, 1112–1115. 10.1126/science.1178534 19965430

[B68] Selmer K. K. Brandal K. Olstad O. K. Birkenes B. Undlien D. E. Egeland T. (2009). Genome-wide linkage analysis with clustered SNP markers. J. Biomol. Screen. 14, 92–96. 10.1177/1087057108327327 19171925

[B69] Shulaev V. Sargent D. J. Crowhurst R. N. Mockler T. C. Folkerts O. Delcher A. L.. (2011). The genome of woodland strawberry (*Fragaria vesca*). Nat. Genet. 43, 109–116. 10.1038/ng.740 21186353 PMC3326587

[B70] Simão F. A. Waterhouse R. M. Ioannidis P. Kriventseva E. V. Zdobnov E. M. (2015). BUSCO: assessing genome assembly and annotation completeness with single-copy orthologs. Bioinformatics 31, 3210–3212. 10.1093/bioinformatics/btv351 26059717

[B71] Sitther V. Zhang D. Harris D. L. Yadav A. K. Zee F. T. Meinhardt L. W.. (2014). Genetic characterization of guava (Psidium guajava L.) germplasm in the United States using microsatellite markers. Genet. Resour. Crop Evol. 61, 829–839. 10.1007/s10722-014-0078-5

[B72] Smit A. Hubley R. (2008). RepeatModeler Open-1.0.

[B73] Stanke M. Keller O. Gunduz I. Hayes A. Waack S. Morgenstern B. (2006). AUGUSTUS: *Ab initio* prediction of alternative transcripts. Nucleic Acids Res. 34, 435–439. 10.1093/nar/gkl200 16845043 PMC1538822

[B74] Takos A. M. Jaffé F. W. Jacob S. R. Bogs J. Robinson S. P. Walker A. R. (2006). Light-induced expression of a MYB gene regulates anthocyanin biosynthesis in red apples. Plant Physiol. 142, 1216–1232. 10.1104/pp.106.088104 17012405 PMC1630764

[B75] Tamazian G. Dobrynin P. Krasheninnikova K. Komissarov A. Koepfli K. P. O'Brien S. J. (2016). Chromosomer: a reference-based genome arrangement tool for producing draft chromosome sequences. Gigascience 5, 1–11. 10.1186/s13742-016-0141-6 27549770 PMC4994284

[B76] Thaipong K. Boonprakob U. Crosby K. Cisneros-Zevallos L. Hawkins Byrne D. (2006). Comparison of ABTS, DPPH, FRAP, and ORAC assays for estimating antioxidant activity from guava fruit extracts. J. Food Compos. Anal. 19, 669–675. 10.1016/j.jfca.2006.01.003

[B77] Thrimawithana A. H. Jones D. Hilario E. Grierson E. Ngo H. M. Liachko I.. (2019). A whole genome assembly of Leptospermum scoparium (Myrtaceae) for mānuka research. New Zeal. J. Crop Hortic. Sci. 47, 233–260. 10.1080/01140671.2019.1657911

[B78] Valdés-Infante J. Becker D. Rodríguez N. Velázquez B. González G. Sourd D.. (2003). Molecular characterization of Cuban accessions of guava (*Psidium guajava* L.), establishment of a first molecular linkage map and mapping of QTLs for vegetative characters. J. Genet. Breed. 57, 349–357.

[B79] Varshney R. K. Graner A. Sorrells M. E. (2005). Genomics-assisted breeding for crop improvement. Trends Plant Sci. 10, 621–630. 10.1016/j.tplants.2005.10.004 16290213

[B80] Vasemägi A. Gross R. Palm D. Paaver T. Primmer C. R. (2010). Discovery and application of insertion-deletion (INDEL) polymorphisms for QTL mapping of early life-history traits in Atlantic salmon. BMC Genomics 11:156. 10.1186/1471-2164-11-156 20210987 PMC2838853

[B81] Velasco R. Zharkikh A. Affourtit J. Dhingra A. Cestaro A. Kalyanaraman A.. (2010). The genome of the domesticated apple (Malus × domestica Borkh.). Nat. Genet. 42, 833–839. 10.1038/ng.654 20802477

[B82] Verde I. Abbott A. G. Scalabrin S. Jung S. Shu S. Marroni F.. (2013). The high-quality draft genome of peach (Prunus persica) identifies unique patterns of genetic diversity, domestication and genome evolution. Nat. Genet. 45, 487–494. 10.1038/ng.2586 23525075

[B83] Verma S. Gupta S. Bandhiwal N. Kumar T. Bharadwaj C. Bhatia S. (2015). High-density linkage map construction and mapping of seed trait QTLs in chickpea (*Cicer arietinum* L.) using Genotyping-by-Sequencing (GBS). Sci. Rep. 5, 1–14. 10.1038/srep17512 26631981 PMC4668357

[B84] Viji G. Harris D. L. Yadav A. K. Zee F. T. (2010). Use of microsatellite markers to characterize genetic diversity of selected accessions of guava (*Psidium guajava*) in the United States. Acta Hortic. 859, 169–176. 10.17660/ActaHortic.2010.859.20

[B85] Wang P. Luo Y. Huang J. Gao S. Zhu G. Dang Z.. (2020). The genome evolution and domestication of tropical fruit mango. Genome Biol. 21, 1–17. 10.1186/s13059-020-01959-8 32143734 PMC7059373

[B86] Wu J. Wang Z. Shi Z. Zhang S. Ming R. Zhu S.. (2013). The genome of the pear (Pyrus bretschneideri Rehd.). Genome Res. 23, 396–408. 10.1101/gr.144311.112 23149293 PMC3561880

[B87] Wu K. Yang M. Liu H. Tao Y. Mei J. Zhao Y. (2014). Genetic analysis and molecular characterization of Chinese sesame (*Sesamum indicum* L.) cultivars using Insertion-Deletion (InDel) and Simple Sequence Repeat (SSR) markers. BMC Genet. 15:35. 10.1186/1471-2156-15-35 24641723 PMC4234512

[B88] Yamaki S. Ohyanagi H. Yamasaki M. Eiguchi M. Miyabayashi T. Kubo T.. (2013). Development of INDEL markers to discriminate all genome types rapidly in the genus Oryza. Breed. Sci. 63, 246–254. 10.1270/jsbbs.63.246 24273419 PMC3770551

[B89] Zhang L. Hu J. Han X. Li J. Gao Y. Richards C. M.. (2019). A high-quality apple genome assembly reveals the association of a retrotransposon and red fruit colour. Nat. Commun. 10, 1–13. 10.1038/s41467-019-09518-x 30940818 PMC6445120

[B90] Zhang Q. Chen W. Sun L. Zhao F. Huang B. Wang J.. (2012). The genome of Prunus mume. Nat. Commun. 3, 1–8. 10.1038/ncomms2290 23271652 PMC3535359

