# Supplementary material for: Development of Genome-Wide Functional Markers Using Draft Genome Assembly of Guava (Psidium guajava L.) cv. Allahabad Safeda to Expedite Molecular Breeding
Source: Front Plant Sci. 2021 Sep 23;12:708332. doi: 10.3389/fpls.2021.708332 (PMC8494772; doi:10.3389/fpls.2021.708332)
Supplement: Supplementary file 1 [file Presentation_1.PPTX]

## Slide 1
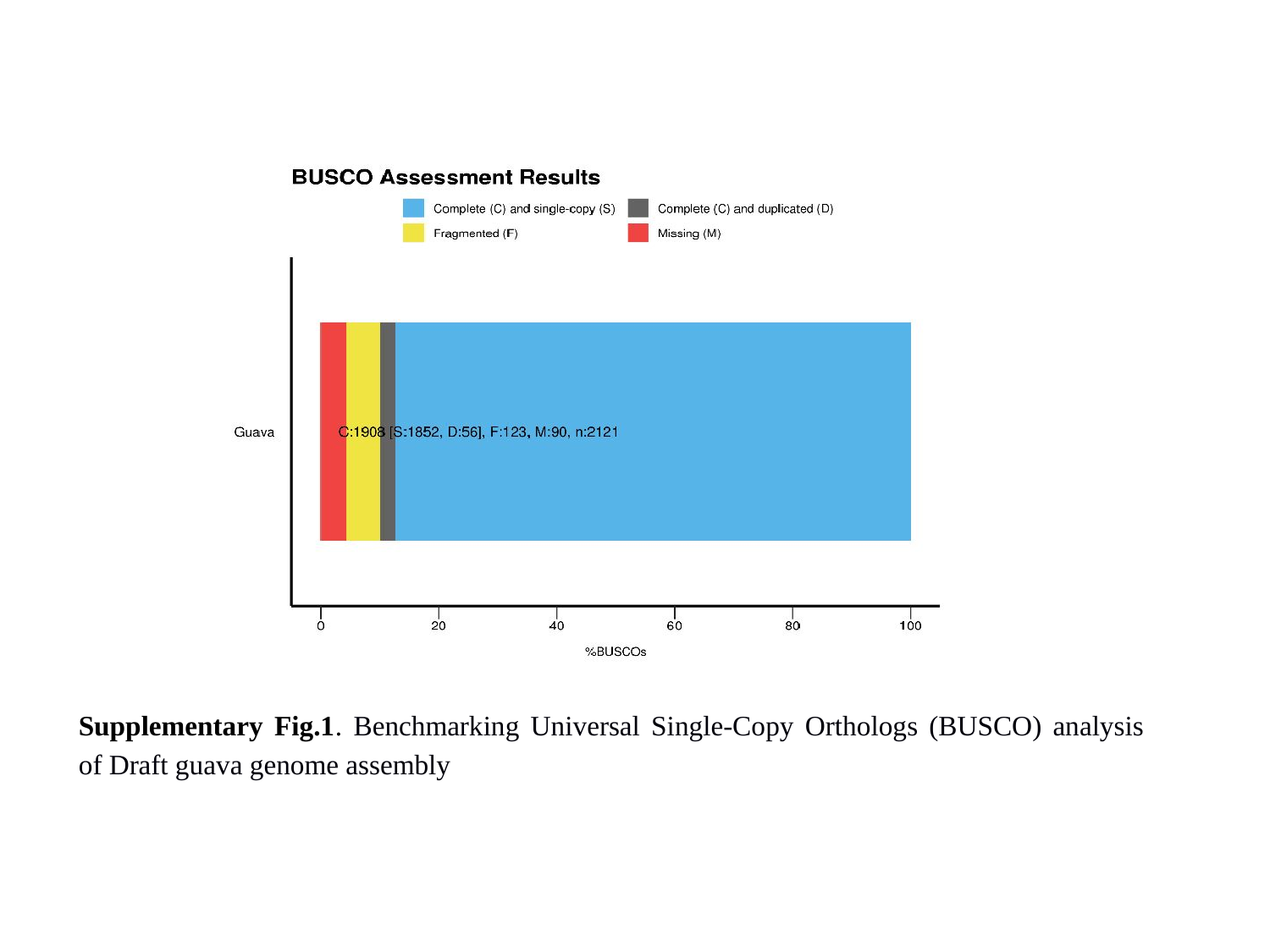

Supplementary Fig.1. Benchmarking Universal Single-Copy Orthologs (BUSCO) analysis of Draft guava genome assembly

## Slide 2
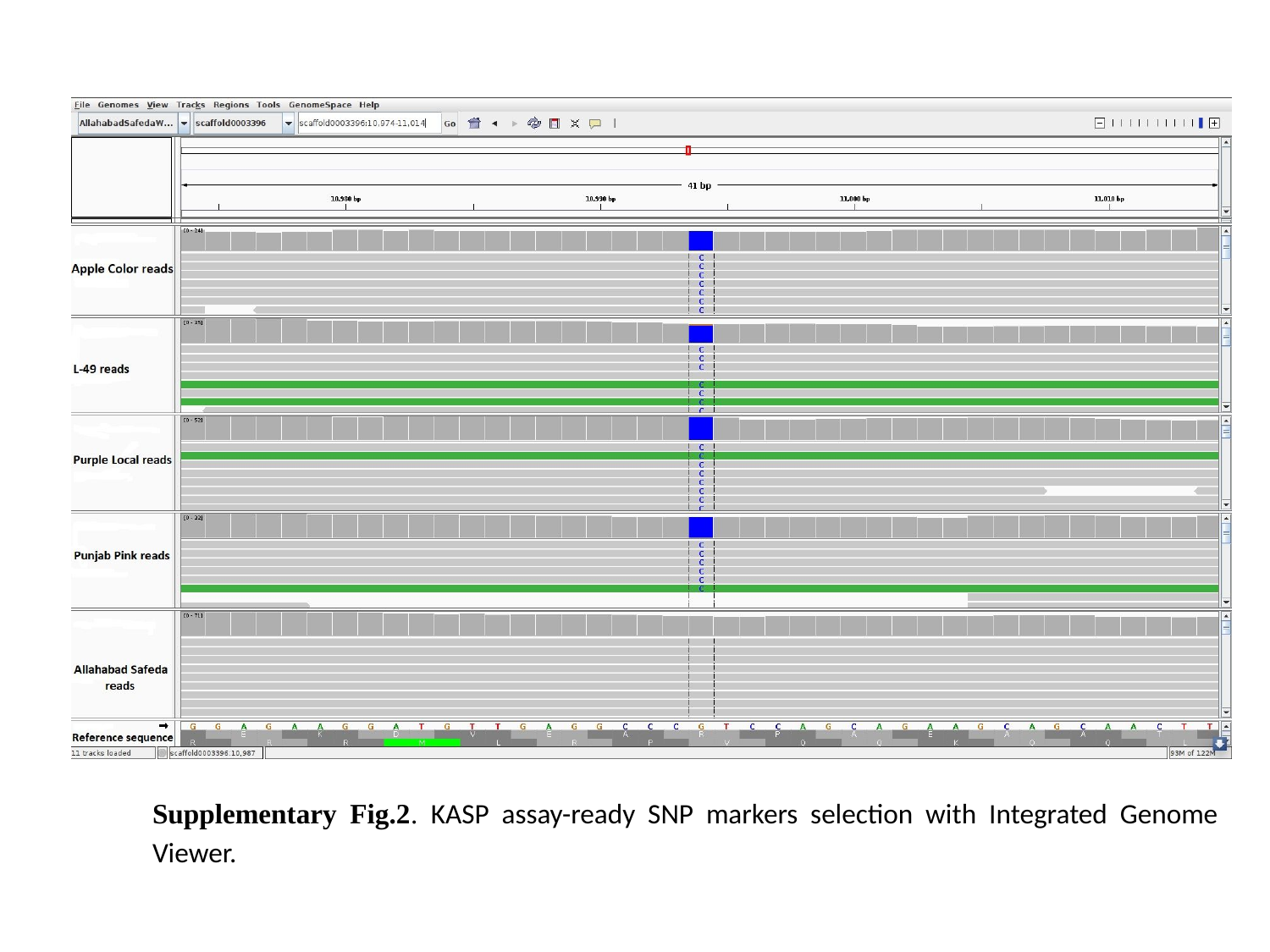

Supplementary Fig.2. KASP assay-ready SNP markers selection with Integrated Genome Viewer.

## Slide 3
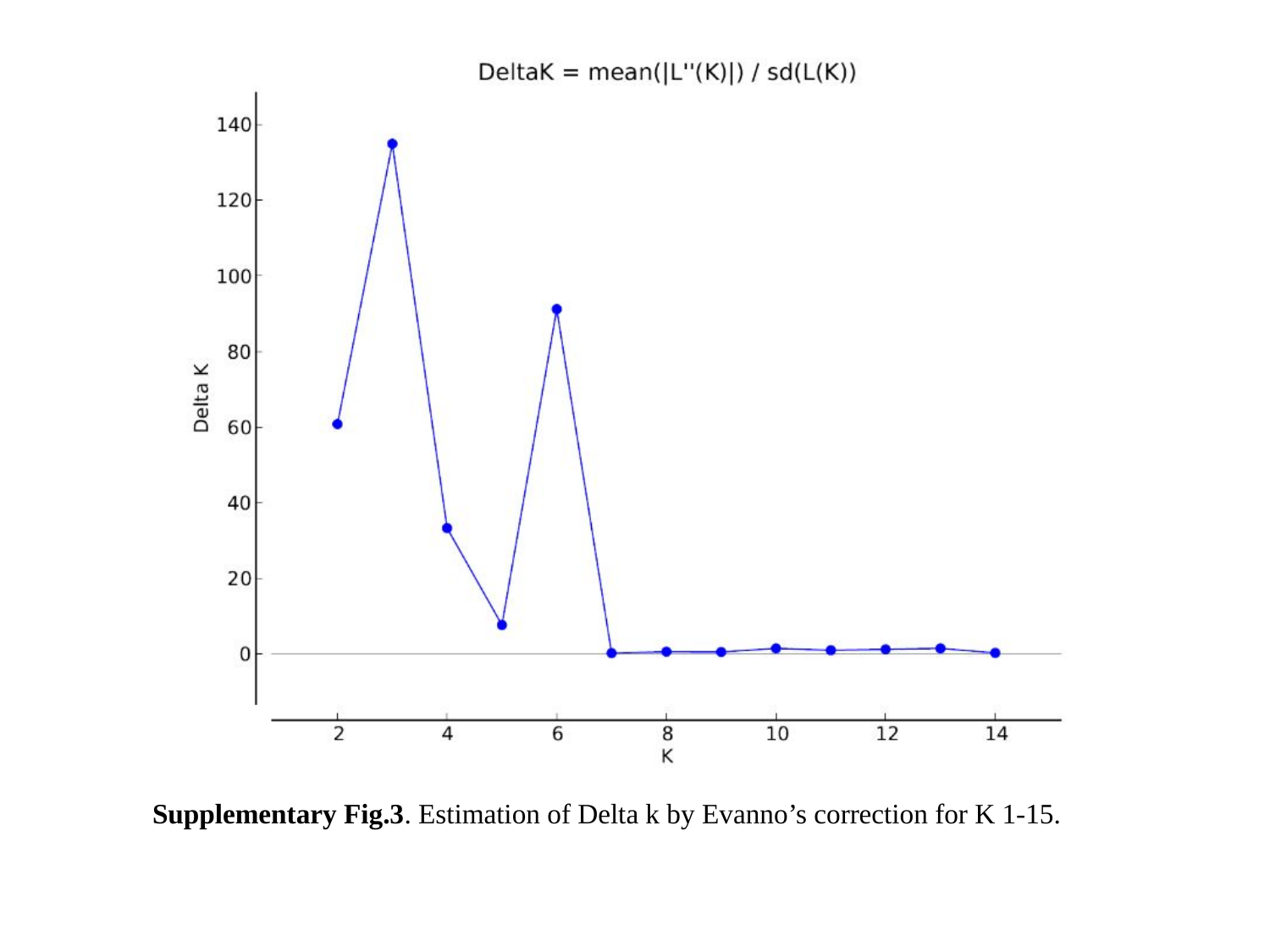

Supplementary Fig.3. Estimation of Delta k by Evanno’s correction for K 1-15.

## Slide 4
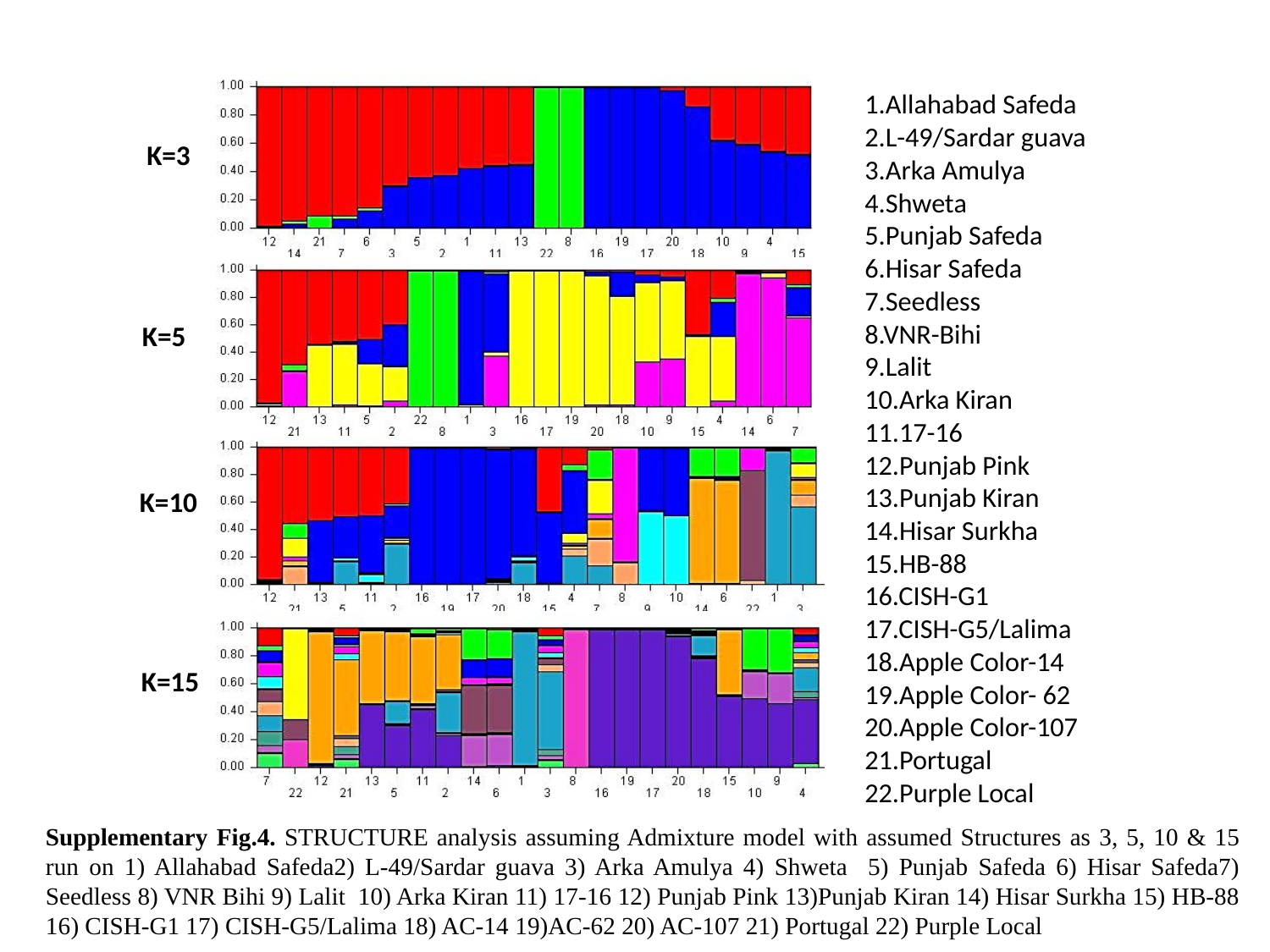

1.Allahabad Safeda
2.L-49/Sardar guava
3.Arka Amulya
4.Shweta
5.Punjab Safeda
6.Hisar Safeda
7.Seedless
8.VNR-Bihi
9.Lalit
10.Arka Kiran
11.17-16
12.Punjab Pink
13.Punjab Kiran
14.Hisar Surkha
15.HB-88
16.CISH-G1
17.CISH-G5/Lalima
18.Apple Color-14
19.Apple Color- 62
20.Apple Color-107
21.Portugal
22.Purple Local
K=3
K=5
K=10
K=15
Supplementary Fig.4. STRUCTURE analysis assuming Admixture model with assumed Structures as 3, 5, 10 & 15 run on 1) Allahabad Safeda2) L-49/Sardar guava 3) Arka Amulya 4) Shweta 5) Punjab Safeda 6) Hisar Safeda7) Seedless 8) VNR Bihi 9) Lalit 10) Arka Kiran 11) 17-16 12) Punjab Pink 13)Punjab Kiran 14) Hisar Surkha 15) HB-88 16) CISH-G1 17) CISH-G5/Lalima 18) AC-14 19)AC-62 20) AC-107 21) Portugal 22) Purple Local
