# Supplementary material for: Development of Genome-Wide Functional Markers Using Draft Genome Assembly of Guava (Psidium guajava L.) cv. Allahabad Safeda to Expedite Molecular Breeding
Source: Front Plant Sci. 2021 Sep 23;12:708332. doi: 10.3389/fpls.2021.708332 (PMC8494772; doi:10.3389/fpls.2021.708332)
Supplement: Supplementary file 2 [file Data_Sheet_1.docx]

**Supplementary Table 1.** tRNASCAN-SE predicted tRNAs in Allahabad Safeda genome

|  | **Unmasked assembly** | **Masked assembly** |
| --- | --- | --- |
| tRNAs decoding Standard 20 AA | 403 | 209 |
| tRNAs with undetermined/unknown isotypes | 2 | 0 |
| Predicted pseudogenes | 53 | 39 |
| Total tRNA | 458 | 248 |
| tRNAs with introns | 29 | 28 |

**Supplementary Table 2.** Statistics of Phylogenetic analysis on *Psidium guajava* (AS), *M*. *acuminata, A*. *comosus, O*. *sativa, Z*. *mays, S*. *lycopersicum, V*. *vinifera, C*. *papaya, A*. *thaliana, G*. *hirustum, P*. *trichocarpa, G*. *max, C*. *sativus, P*. *persica, C*. *clementina, F*. *vesca, M*. *domestica, Z*. *jujuba and E*. *grandis* with OrthoFinder

| Number of species | 19 |
| --- | --- |
| Number of genes | 663622 |
| Number of genes in orthogroups | 594430 |
| Number of unassigned genes | 69192 |
| Percentage of genes in orthogroups | 89.6 |
| Percentage of unassigned genes | 10.4 |
| Number of orthogroups | 35370 |
| Number of species-specific orthogroups | 14455 |
| Number of genes in species-specific orthogroups | 58266 |
| Percentage of genes in species-specific orthogroups | 8.8 |
| Mean orthogroup size | 16.8 |
| Median orthogroup size | 5 |
| G50 (assigned genes) | 34 |
| G50 (all genes) | 30 |
| O50 (assigned genes) | 4623 |
| O50 (all genes) | 5704 |
| Number of orthogroups with all species present | 6407 |
| Number of species-specific orthogroups in AS | 56 |
| Number of unassigned genes of AS | 764 |

**Supplementary Table 3:** Insertion deletion and Simple sequence repeat region based molecular markers mapped or unmapped to pseudochromosomes of draft guava genome assembly - validated on 6% Poly-acrylamid gel electrophoresis system in 22 diverse cultivars/genotypes and related species *P. cattleianum* – strawberry & lemon guava. AS: Allahabad Safeda; PL: Purple Local/ Purple guava; SG/L-49: Sardar guava; PP: Punjab Pink; AC: Apple Color/ CISH-G5/Lalima

| **S.No.** | **Marker_Name** | **Primer sequence** | **Allahabad Safeda Transcriptome Assembly_component_ID/ Genomic Scaffold_ID** | **Function/ Description** | **Gene Ontology_Annotation** | ***In silico* polymorphic (Genotype)** | **6% Poly-acrylamid gel based Validation** | **Expected size (bp)** | **Amplicon Length** | **Polymorphic**  **Information**  **Content** |  |  |  |  |  |  |  |  |
| --- | --- | --- | --- | --- | --- | --- | --- | --- | --- | --- | --- | --- | --- | --- | --- | --- | --- | --- |
| 1 | AS/PL_InDel-2 | CAGCTTTACATCCACCTTCACA  AGTGGATGCACTGATCTTGATG | comp19461_c0_seq1/scaffold0001640 | stress response nst1-like | C:GO:0016020; C:GO:0016021 | Purple local | Polymorphic | 100 | ~80-100 | 0.4163 |  |  |  |  |  |  |  |  |
| 2 | AS/PL_InDel-5 | TGTTCAGAGATTTGAGAGTTCAGG  CCGTTCCTGACAAAAGATCA | comp18910_c0_seq1/scaffold0012219 | mitochondrial pyruvate carrier 2-like | P:GO:0006850; C:GO:0005743 | Purple local | Polymorphic | 114 | ~100-150 | 0.4688 |  |  |  |  |  |  |  |  |
| 3 | AS/PL_InDel-6 | CTCGAACCAATTTACAGTGCAT  GGTTTGTCCAAGAGCAGAAGTC | comp27128_c0_seq37/scaffold0004517 | hypothetical protein EUGRSUZ_H04996 | P:GO:0016310; F:GO:0016301 | Purple local | Polymorphic | 90 | ~75-100 | 0.4770 |  |  |  |  |  |  |  |  |
| 4 | AS/PL_InDel-7 | TATTTGCAGGAGCAAAGTGTTG  CCAAAAGGCCTTCCTTCTAATC | comp26340_c0_seq40/scaffold0001015/ | mitochondrial phosphatidylserine decarboxylase |  | Purple local | Polymorphic | 99 | ~70-100 | 0.3512 |  |  |  |  |  |  |  |  |
| 5 | AS/PL_InDel-9 | AAAAGTGTGTTGGCTTTGGA  TCGACGTGGTAATGAAGAAAGA | comp5992_c0_seq1/ scaffold0000738 |  |  | Purple local | Polymorphic | 115 | ~95-100 | 0.4959 |  |  |  |  |  |  |  |  |
| 6 | AS/PL_InDel-10 | ATCATCGCTACCAGAATCATCA  ATGGGAGGTTCGAGAAAATAGG | comp23961_c0_seq37/ scaffold0002766 | phosphoinositide phosphatase SAC3 | P:GO:0007033; C:GO:0016021; F:GO:0004571; P:GO:0036092; F:GO:0042578 | Purple local | Polymorphic | 103 | ~70-100 | 0.2676 |  |  |  |  |  |  |  |  |
| 7 | AS/PL_InDel-11 | CATCCGTTTGCTTGAACCAT  ATACCGAAAGTTTCTCGGAACA | comp14858_c0_seq1/ scaffold0006465 | not found |  | Purple local | Polymorphic | 116 | ~100-120 | 0.3967 |  |  |  |  |  |  |  |  |
| 8 | AS/PL_InDel-14 | GAATGTGAACGCGGAAATCT  GATGTATGGGACAAGAACGAGA | comp34820_c0_seq1/ scaffold0014990 | E3 ubiquitin- ligase ATL6-like | F:GO:0046872; C:GO:0016020 | Purple local | Polymorphic | 110 | ~95-150 | 0.4907 |  |  |  |  |  |  |  |  |
| 9 | AS/PL_InDel-16 | ACCGAGTGAACTCGTCTAGCA  CGACAATGAGAACAGTGACAGC | comp9539_c0_seq2/ scaffold0008025 | PREDICTED: uncharacterized protein LOC104425418 isoform X1 |  | Purple local | Polymorphic | 119 | ~100-130 | - |  |  |  |  |  |  |  |  |
| 10 | AS/PL_InDel-17 | TCCAACATATTAAGGCTCTGTTTG  AGGGTTTTTGGTTGCTAAGTGA | comp2749_c0_seq1/ scaffold0000662 |  |  | Purple local | Polymorphic | 120 | ~70-125 | - |  |  |  |  |  |  |  |  |
| 11 | AS/PL_InDel-18 | AGAAGACCCAGAAATCAACGAG  TCACATCGAAATCAAGAACAGG | comp1945_c0_seq1/ scaffold0009154 |  |  | Purple local | Polymorphic | 120 | ~100-140 | 0.3254 |  |  |  |  |  |  |  |  |
| 12 | AS/PL_InDel-20 | CAATGCCACTTCAGATTTTCAA  AAGGGTGGAATCTGAACAAATG | comp41376_c0_seq1/ scaffold0013736 | hypothetical protein |  | Purple local | Polymorphic | 80 | ~60-100 | 0.0907 |  |  |  |  |  |  |  |  |
| 13 | AS/PL_InDel-24 | ATGCCTGACGAGCACAATTAC  TCCCTCCAAAATACATGAAGTG | comp18555_c0_seq1/ scaffold0010866 |  |  | Purple local | Non-Polymorphic | 120 |  | 0.2355 |  |  |  |  |  |  |  |  |
| 14 | AS/PL_InDel-25 | GGTCTGGTTGCCGATTCCTA  CGTCCTAGCTCAGCAATAAACA | comp11038_c0_seq2/ scaffold0000005 | RNA polymerase sigma-24 subunit ECF subfamily | F:GO:0003677; F:GO:0016987; F:GO:0003700; F:GO:0008270; P:GO:0006351; F:GO:0046872; P:GO:0006355; P:GO:0006352 | Purple local | Polymorphic | 120 | ~95-200 | - |  |  |  |  |  |  |  |  |
| 15 | AS/PL_InDel-27 | AGCTGTCGAAGCTGAGATGG  GTCAAATGGCTCGGGTCTTC | comp27191_c0_seq54/ scaffold0004467 | Phosphoinositide phosphatase family isoform 1 | F:GO:0043813; P:GO:0009826; P:GO:0007010; P:GO:0036092; C:GO:0005794; P:GO:0009832 | Purple local | Non-Polymorphic | 115 | ~95-105 | - |  |  |  |  |  |  |  |  |
| 16 | AS/PL_InDel-28 | TCTCATGTGCAAGCCAAATTAC  TTCTGGAACAGAATGGTTGC | comp26876_c1_seq16/ scaffold0000690 |  |  | Purple local | Polymorphic | 110 | ~100-110 | 0.1653 |  |  |  |  |  |  |  |  |
| 17 | AS/PL_InDel-29 | CGAATCTTCTCGAACTGACTCC  CAAGCAAGAACTTGAATCGAGA | comp16580_c0_seq1/ scaffold0000235 | probable pyridoxal biosynthesis | P:GO:0042823; P:GO:0055085; F:GO:0003824; F:GO:0046982; C:GO:0016021; P:GO:0042819; C:GO:0005829 | Purple local | Polymorphic | 115 | ~105-135 | 0.4339 |  |  |  |  |  |  |  |  |
| 18 | AS/PL_InDel-30 | GTACGTGTGTGTCGTCCTCTTC  AACCGAATTGAGGAGGTTTTG | comp23700_c0_seq14/ scaffold0002895 | UPF0235 At5g63440 | C:GO:0005634 | Purple local | Polymorphic | 100 | ~95-100 | 0.2676 |  |  |  |  |  |  |  |  |
| 19 | AS/PL_InDel-33 | TTATGTTCATGCCCTGAGTTTG  AATCTCGTGGACGAATTTGACT | comp18659_c0_seq2/ scaffold0001279 |  |  | Purple local | Polymorphic | 104 | ~90-100 | 0.3628 |  |  |  |  |  |  |  |  |
| 20 | AS/PL_InDel-34 | ATTGAGAGGTTACATGCGTCGT  AGCAAGGCGTTAACTCAGTCTC | comp20685_c0_seq1/ scaffold0005006 | ankyrin repeat-containing | F:GO:0008168; P:GO:0032259 | Purple local | Polymorphic | 81 | ~70-80 | 0.1653 |  |  |  |  |  |  |  |  |
| 21 | AS/PL_InDel-36 | CCTTACCACTGCCATGGATAC  GGTTATGGCATGAGTGGTTTTT | comp20747_c0_seq16/ scaffold0001430 | heterogeneous nuclear ribonucleo 1 | F:GO:0000166; F:GO:0003676 | Purple local | Polymorphic | 118 | ~100-130 | 0.1653 |  |  |  |  |  |  |  |  |
| 22 | AS/PL_InDel-37 | GTACACTGTCACCTTCGTCTCG  CCTAACTTGGTTCTGGGCATT | comp24390_c0_seq1/ scaffold0009953 | subtilisin-like protease | C:GO:0009506; P:GO:0006508; C:GO:0016021; F:GO:0004252; C:GO:0005794; C:GO:0009505 | Purple local | Polymorphic | 106 | ~95-105 | - |  |  |  |  |  |  |  |  |
| 23 | AS/PL_InDel-39 | CTTCAGGAGCTCCGACAGTCT  GCCTGGACTCTCTGTAGATGAT | comp27099_c2_seq12/ scaffold0001047 | stromal cell-derived factor 2 | P:GO:0000032; P:GO:0007275; C:GO:0016021; P:GO:1900101; C:GO:0031502; P:GO:0035269; P:GO:0044845; F:GO:0004169 | Purple local | Polymorphic | 117 | ~100-115 | - |  |  |  |  |  |  |  |  |
| 24 | AS/PL_InDel-40 | CGTATACGTTTCGTGACTTTCG  GGAATGCATTTTCTTCTTCACC | comp35031_c0_seq1/ scaffold0006066 |  |  | Purple local | Polymorphic | 87 | ~70-95 | 0.2975 |  |  |  |  |  |  |  |  |
| 25 | AS/PL_InDel-43 | CGCTCTCTGAATCTGGAATCTC  CTGAGGAGGAGGAACAAATACG | comp24875_c0_seq51/ scaffold0003226/ | hydroxyproline-rich glyco | C:GO:0044464 | Purple local | Polymorphic | 109 | ~100-125 | 0.3878 |  |  |  |  |  |  |  |  |
| 26 | AS/PL_InDel-45 | CCCTAATTTTTGCGGTTTGA  AACTACCTTACGGCTCACGATG | comp26567_c0_seq123/ scaffold0000871 | polypyrimidine tract-binding homolog 2-like isoform X1 |  | Purple local | Polymorphic | 114 | ~100-115 | - |  |  |  |  |  |  |  |  |
| 27 | AS/PL_InDel-46 | GGATCGTTTTAAGACTCGATTG  GGACGAGAAGGAATATGACGAC | comp25316_c0_seq7/ scaffold0016169 | serine carboxypeptidase-like 35 | F:GO:0004180; P:GO:0006508 | Purple local | Polymorphic | 125 | ~100-125 | 0.0868 |  |  |  |  |  |  |  |  |
| 28 | AS/PL_InDel-47 | AGCCAATGTTAGCCTTTTCGT  AGGGATGAAAGCTAGCACACTG | comp44519_c0_seq1/ scaffold0000246 |  |  | Purple local | Polymorphic | 90 | ~90-100 | 0.4742 |  |  |  |  |  |  |  |  |
| 29 | AS/PL_InDel-49 | CCATAGTTGGCAACAAGTGTGT  CCTTGAGACCTCGAAAGCAT | comp60468_c0_seq1/ scaffold0001044 | hydroxyproline-rich glyco |  | Purple local | Polymorphic | 99 | ~95-100 | 0.2014 |  |  |  |  |  |  |  |  |
| 30 | AS/PP_InDel-2 | GGCTAGGCTTCTATGAGGCATT  AAACAGCAGGTTTCAGGAAGTC | comp14545_c0_seq1/ scaffold0008359 | root primordium defective 1 | F:GO:0016787 | Punjab Pink | Polymorphic | 84 | ~70-100 | 0.3666 |  |  |  |  |  |  |  |  |
| 31 | AS/PP_InDel-3 | GTTCTCAGTGTACCGGGAAAAG  ATTTGGCACAAGAGGGAAAAG | comp23812_c0_seq9/ scaffold0006826 |  |  | Punjab Pink | Polymorphic | 99 | ~75-115 | 0.2725 |  |  |  |  |  |  |  |  |
| 32 | AS/PP_InDel-11 | GAAATGGTATTTTGGCATGGTC  GTTCAAGCCTGTACCAGAGTCA | comp26975_c0_seq97/ scaffold0003851 | Beta-galactosidase 17 |  | Punjab Pink | Polymorphic | 105 | ~80-125 | 0.3457 |  |  |  |  |  |  |  |  |
| 33 | AS/G5_InDel-6 | CAAGGAAATCAAGGCTCAAAAC  TCCTTTTTGGGTCTGTGAAAGT | comp23868_c0_seq16/ scaffold0002068 | dof zinc finger -like | F:GO:0003677; P:GO:0006355 | Apple Color | Non-Polymorphic | 117 |  | - |  |  |  |  |  |  |  |  |
| 34 | AS/G5_InDel-7 | GATGTATGGGACAAGAACGAGA  GAATGTGAACGCGGAAATCT | comp34820_c0_seq1/ scaffold0014990 | E3 ubiquitin- ligase ATL6-like | F:GO:0046872; C:GO:0016020 | Apple Color | Polymorphic | 110 | ~80-140 | 0.3703 |  |  |  |  |  |  |  |  |
| 35 | AS/G5_InDel-8 | GTTTAACCTTGCGTTCAGGATT  CTTAGCCTCTGCAGGACCTC | comp23163_c0_seq7/ scaffold0002178 | ACT domain-containing family | F:GO:0016779; P:GO:0008152; F:GO:0016597 | Apple Color | Polymorphic | 102 | ~85-120 | 0.3666 |  |  |  |  |  |  |  |  |
| 36 | AS/G5_InDel-10 | GGCTTCTATGAGGCATTAGCTC  ACGAGCATGAAGCGTAAGATTT | comp14545_c0_seq3/ scaffold0008359 | DEAD-box ATP-dependent RNA helicase 37-like | F:GO:0003676; F:GO:0005524; P:GO:0010501; F:GO:0004004 | Apple Color | Polymorphic | 108 | ~90-125 | 0.3666 |  |  |  |  |  |  |  |  |
| 37 | AS/G5_InDel-12 | CCCTCTCACCTACCCAAACC  GATGGAATTTGATAGGCACGAC | comp19536_c2_seq1/ scaffold0003571 |  |  | Apple Color | Polymorphic | 116 | ~95-110 | 0.3180 |  |  |  |  |  |  |  |  |
| 38 | AS/SG_InDel-2 | CAGATGTATGGGACAAGAACGA  GAATGTGAACGCGGAAATCT | comp34820_c0_seq1/ scaffold0014990 | E3 ubiquitin- ligase ATL6-like | F:GO:0046872; C:GO:0016020 | L-49 | Polymorphic | 112 | ~95-130 | 0.3703 |  |  |  |  |  |  |  |  |
| 39 | AS/SG_InDel-3 | CAAGCCATCGTAATCAGCTTTA  ATCAGGAATGGCTTTTCGATAG | comp19461_c0_seq1/ scaffold0001640 | stress response nst1-like | C:GO:0016020; C:GO:0016021 | L-49 | Polymorphic | 92 | ~65-100 | 0.3180 |  |  |  |  |  |  |  |  |
| 40 | AS/SG_InDel-5 | CACGTGTTCAGAGATTTGAGAGTT  TCCTGACAAAAGATCATCCTGA | comp18910_c0_seq1/ scaffold0012219 | mitochondrial pyruvate carrier 2-like | P:GO:0006850; C:GO:0005743 | L-49 | Polymorphic | 114 | ~95-105 | 0.3557 |  |  |  |  |  |  |  |  |
| 41 | AS/SG_InDel-6 | AAATCGAACGGGAGAAGTGTT  ATGAAGCTCGCATATCTCGATT | comp19962_c0_seq2/ scaffold0011537 | DEAD-box ATP-dependent RNA helicase 37-like | F:GO:0003676; F:GO:0005524; P:GO:0010501; F:GO:0004004 | L-49 | Polymorphic | 106 | ~80-105 | 0.0830 |  |  |  |  |  |  |  |  |
| 42 | AS/SG_InDel-7 | GCAAGAAGTTGCTGACACTTTG  AGAGGGGCCCTATCTCTGTG | comp27039_c0_seq22/ scaffold0007417 | UDP-galactose transporter 2 | F:GO:0005338; P:GO:0015780; C:GO:0016021; P:GO:0008643; F:GO:0016740; P:GO:1901679 | L-49 | Polymorphic | 114 | ~95-105 | 0.1812 |  |  |  |  |  |  |  |  |
| 43 | AS/SG_InDel-8 | AGGAGGTGCAGATGTCTTGAAT  TAGCTGAAAGTGCACAAGAGGA | comp24715_c0_seq63/ scaffold0005984 | asparagine-tRNA chloroplastic mitochondrial-like | F:GO:0003676; F:GO:0005524; F:GO:0004816; P:GO:0006421; C:GO:0000139; P:GO:0048208 | L-49 | Polymorphic | 107 | ~95-120 | 0.1516 |  |  |  |  |  |  |  |  |
| 44 | AS/SG_InDel-9 | GAATTTTGATTCGGACGACAAG  AGGCTCTATTTATTTCGCGGTA | comp13374_c0_seq3/ scaffold0002090 | WRKY DNA-binding 7 isoform 1 | F:GO:0003700; P:GO:0006355; F:GO:0043565 | L-49 | Polymorphic | 119 | ~100-125 | 0.3180 |  |  |  |  |  |  |  |  |
| 45 | AS/SG_InDel-10 | CTTAAACTCCGCACAAAAATCC  CGGAAGCGAAAAGGAATCTAAT | comp20612_c0_seq4/ scaffold0000199 | 40S ribosomal S2-4-like | F:GO:0003723; F:GO:0003735; C:GO:0022627; P:GO:0006412 | L-49 | Polymorphic | 114 | ~105-130 | - |  |  |  |  |  |  |  |  |
| 46 | AS/SG_InDel-12 | CGCTGTCTAACATTTCTTGGAG  TAGCATGTTTGAGAAGGGGAAG | comp31977_c0_seq1/ scaffold0011537 | hypothetical protein HELRODRAFT_181557 |  | L-49 | Polymorphic | 120 | ~100-125 | 0.1190 |  |  |  |  |  |  |  |  |
| 47 | AS/SG_InDel-13 | AGCATTGGGTTCTTGTCAATCT  GACCACCTGACTCCTCGTACTT | comp21605_c0_seq6/ scaffold0001185 |  |  | L-49 | Polymorphic | 114 | ~100-125 | 0.3703 |  |  |  |  |  |  |  |  |
| 48 | AS/SG_InDel-14 | CTGGACAGCCGGTATGATTT  CCAAAGAAGGCCCATAGACATA | comp26408_c0_seq65/ scaffold0024288 | K(+) efflux antiporter 4 isoform X1 |  | L-49 | Polymorphic | 108 | ~95-100 | 0.3703 |  |  |  |  |  |  |  |  |
| 49 | AS/SG_InDel-15 | ACCTTGATACACAGCGGTTACG  AGAATCTCGTCAGCTGGCTATC | comp14190_c0_seq1/ scaffold0006289 | pyrophosphate-energized membrane proton pump 3 | F:GO:0004427; P:GO:0015992; P:GO:0055085; C:GO:0016021; F:GO:0009678 | L-49 | Non-polymorphic | 87 |  | - |  |  |  |  |  |  |  |  |
| 50 | AS/SG_InDel-18 | GCTCTGAATTTCATCAATCACC  TCCCATTTGAGAGGGTTGAATA | comp22485_c0_seq3/ scaffold0014261 | transducin family |  | L-49 | Polymorphic | 115 | ~110-200 | 0.0434 |  |  |  |  |  |  |  |  |
| 51 | AS/SG_InDel-20 | GATGATACCAGGAAGGGATGAC  GAGCTCTCTTGAGCTGAACCAC | comp19948_c0_seq3/ scaffold0004058 | primary amine oxidase-like | P:GO:0009308; F:GO:0005507; F:GO:0048038; F:GO:0008131; C:GO:0016021; P:GO:0055114 | L-49 | Polymorphic | 117 | ~95-125 | 0.3557 |  |  |  |  |  |  |  |  |
| 52 | ASgco_2369224_SSR1 | CTCCTTCTAGGTTCTTCTAGGTTC  CAGGGGAGCTCACAAGCAGA | comp27093_c0_seq7/ scaffold0006778 |  |  | Purple local |  | 111 | Not amplified | - |  |  |  |  |  |  |  |  |
| 53 | ASgco_2388028_SSR3 | TCAAATCTCAAATGAACAGACATCA  CATTGGGATGCGCTCTCCTC | scaffold0000196 |  |  | Purple local | Polymorphic | 119 | ~120 | 0.2533 |  |  |  |  |  |  |  |  |
| 54 | ASgsc_1238_SSR4 | GTGGAAATAGCGTTTCGCAGTG  GCGCTTGATGTAGTGGAGTGACC | comp13689_c2/ scaffold0002088 |  |  | Purple local |  |  | Amplicon size beyond range | - |  |  |  |  |  |  |  |  |
| 55 | ASgsc_18206_SSR9 | GGCTACTCACGAGAAGGGTCCA  TTGCTGGAAGTGTTGACCAATCAT | scaffold0000936 |  |  | Purple local | Polymorphic | 116 | ~100 | 0.3618 |  |  |  |  |  |  |  |  |
| 56 | ASgsc_19427_SSR10 | CGAGATACAGTTGAAAGAGCTTGTG  GCTCATTTTGTCCCATGGAAGTT | scaffold0003219 |  |  | Purple local | Non-polymorphic | 110 | ~100-150 | - |  |  |  |  |  |  |  |  |
| 57 | ASgsc_22178_SSR12 | TACAGGCAATGATGGGTGGTTC  ATGAAGGGTCCACGTCAAGGAA | comp24080_c0_seq1/ scaffold0002516 | Zinc finger family |  | Purple local | Polymorphic | 107 | ~100 |  |  |  |  |  |  |  |  |  |
| 58 | ASgsc_23098_SSR13 | AGGCCACGTTCTGGTGATGACT  TGGAGCTAAGGCGCCAGAAG | scaffold0004114 |  |  | Purple local | Non-polymorphic | 105 | ~100-130 | - |  |  |  |  |  |  |  |  |
| 59 | ASgsc_27290_SSR15 | TGGACTTATTCCCTCACCAATGC  TTACCTGCATGTGCTTGTGTGC | comp23838_c0_seq30/ scaffold0001810 | argonaute 1 |  | Purple local | Polymorphic | 120 | ~100-150 | 0.3729 |  |  |  |  |  |  |  |  |
| **Unmapped Markers to Draft Genome Assembly** | | | | | | | | | |  |  |  |  |  |  |  |  |  |
| 60 | AS/PL_InDel-1 | AACTGTTCCAGTCGAGGATGAT  ATTCAGCGCTAAGGAACAGG | comp10880_c0_seq1/ scaffold0000881 |  |  | Purple local | Polymorphic | 114 | ~125 | 0.15161 |  |  |  |  |  |  |  |  |
| 61 | AS/PL_InDel-3 | TGCAAGCAAATGAGCAAAATAG  TCTGTTAGTTTTCTCAAAGCGGTA | comp9025_c0_seq1/ scaffold0000523 | myosin-11-like isoform X1 |  | Purple local | Non-Polymorphic | 115 | ~100-120 | 0.3047 |  |  |  |  |  |  |  |  |
| 62 | AS/PL_InDel-4 | CATCAGAATCATCTATTCTTGTTGC  TGGTTGGAACTTGTAATTTTGC | comp14928_c0_seq2/ scaffold0003069 | hypothetical protein EUGRSUZ_C00488 | C:GO:0016020; C:GO:0016021 | Purple local | Polymorphic | 80 | ~70-80 | 0.1812 |  |  |  |  |  |  |  |  |
| 63 | AS/PL_InDel-8 | CGGATCATTGAGGGAGTAACAT  GGTCAGTCAGATCCAAGAATCA | comp24400_c0_seq3/ scaffold0003400 | E3 ubiquitin- ligase RHA1B-like | F:GO:0008270 | Purple local | Polymorphic | 117 | ~70-160 | 0.3488 |  |  |  |  |  |  |  |  |
| 64 | AS/PL_InDel-12 | CGAATACACACACAAACCGTGT  GCTTTGGTGATCCAAGTCAAAT | comp9401_c0_seq1/ scaffold0004137 |  |  | Purple local | Non-Polymorphic | 105 | ~70-100 | - |  |  |  |  |  |  |  |  |
| 65 | AS/PL_InDel-13 | TAAGGCGTTAAATTTGGTGTCC  GCTATCAGGTTTGGAAAACGTC | comp33066_c0_seq1/ scaffold0001021 |  |  | Purple local | Polymorphic | 110 | ~100-150 | 0.1239 |  |  |  |  |  |  |  |  |
| 66 | AS/PL_InDel-15 | CGAGGGTCAGTATTGTGGTCTA  ATTAACTTCTTGGATGCGTTGG | comp33186_c0_seq1/ scaffold0000953 |  |  | Purple local | Non-Polymorphic | 119 | ~100-125 | - |  |  |  |  |  |  |  |  |
| 67 | AS/PL_InDel-19 | TCAGTCATTGCCTATTGATCTACAA  TTGCATTGATATTGCTTCTTTCA | comp19584_c0_seq5/ scaffold0000459 | dentin sialophospho -like isoform X3 |  | Purple local | Polymorphic | 86 | ~100 | 0.1516 |  |  |  |  |  |  |  |  |
| 68 | AS/PL_InDel-21 | GCGTAGATACATTGGAGAATGACA  GCTCGTCCCTAAAGCTCCTTAT | comp25013_c0_seq49/ scaffold0000944 | dedicator of cytokinesis 8 isoform X1 | P:GO:0008360; P:GO:0007264; P:GO:0008064; F:GO:0005089; C:GO:0005886; P:GO:0043547; C:GO:0005634; P:GO:0009958; P:GO:0010928; P:GO:0016192; C:GO:0005829; C:GO:0070971; C:GO:0019898 | Purple local | Polymorphic | 90 | ~85-100 | 0.3398 |  |  |  |  |  |  |  |  |
| 69 | AS/PL_InDel-22 | CAAAATACCTCTGTTTTGCTCA  GAAGGTCTTGACGGGAACAA | comp25816_c0_seq46/ scaffold0009520 | Radical SAM superfamily isoform 1 |  | Purple local | Polymorphic | 116 | ~100-130 | 0.2896 |  |  |  |  |  |  |  |  |
| 70 | AS/PL_InDel-23 | TACCTTTGCTTCTGACGATGAC  ATGTACTTCAACGCTTGCTTCA | comp14587_c0_seq1/ scaffold0003201 | hypothetical protein EUGRSUZ00188 | F:GO:0042578 | Purple local | Polymorphic | 120 | ~100-140 | 0.2896 |  |  |  |  |  |  |  |  |
| 71 | AS/PL_InDel-26 | CGAGCATGAAACTAGAACAAGC  GTCGTTCACTTGCTTCCCTTAC | comp18915_c0_seq3/ scaffold0001940 |  |  | Purple local | Polymorphic | 109 | ~100-130 | - |  |  |  |  |  |  |  |  |
| 72 | AS/PL_InDel-31 | TAGGCGTGAAGAAATCAATGAC  GGACAAAAGATGGGCTTCATAC | comp25253_c0_seq7/ scaffold0001186 | pentatricopeptide repeat-containing At4g17616 |  | Purple local | Polymorphic | 120 | ~100-140 | 0.2533 |  |  |  |  |  |  |  |  |
| 73 | AS/PL_InDel-32 | AAGAAGACCTGAAGTTGCATCA  CCTTCTTCCTATTTGTGCTTTTGG | comp17869_c0_seq2/ scaffold0017099 |  |  | Purple local | Polymorphic | 111 | ~95-110 | 0.3398 |  |  |  |  |  |  |  |  |
| 74 | AS/PL_InDel-35 | GAAAGTTGGCTAACGAGTCAGG  ACGCATGTAAAACCCAATCC | comp26239_c0_seq3/ scaffold0001575 | vacuolar sorting-associated 2 homolog 1 |  | Purple local | Polymorphic | 94 | ~95-115 | 0.3047 |  |  |  |  |  |  |  |  |
| 75 | AS/PL_InDel-38 | TCCATCAATTAGGGTCCAAAAC  ACAAGTAGGACTGCAGCGTGTA | comp25164_c0_seq22/ scaffold0000357 | cancer-related nucleoside-triphosphatase | F:GO:0005524; P:GO:0016311; F:GO:0098519 | Purple local | Polymorphic | 95 | ~85-95 | 0.3729 |  |  |  |  |  |  |  |  |
| 76 | AS/PL_InDel-41 | GAGAATTGTCGACTTGTTGTGA  CAGGAAAAGCAAACACGATACA | comp14062_c0_seq1/ scaffold0000018 | RNA polymerase-associated RTF1 homolog | F:GO:0003677; P:GO:0045893; P:GO:0016570; C:GO:0016593; P:GO:0009910; P:GO:0006368 | Purple local | Polymorphic | 89 | ~75-100 | 0.3557 |  |  |  |  |  |  |  |  |
| 77 | AS/PL_InDel-42 | GAACAACTTTTCCTCCGTCAAT  ATTTCCCTAACAACCTCTGTGG | comp25134_c0_seq22/ scaffold0000192 | zinc finger 830 |  | Purple local | Polymorphic | 92 | ~75-100 | 0.1516 |  |  |  |  |  |  |  |  |
| 78 | AS/PL_InDel-44 | AAAAATCACGAAGCGGTGTT  CGTGGTGGCGACTGATAAGA | comp26454_c0_seq18/ scaffold0005883 | zinc induced facilitator-like 1-like isoform X1 |  | Purple local | Polymorphic | 111 | ~100-150 | - |  |  |  |  |  |  |  |  |
| 79 | AS/PL_InDel-48 | AGTTCCCCTAGGCTATCTTTGA  TGGGAGATGCCTATATCCATTA | comp57676_c0_seq1/ scaffold0002049 |  |  | Purple local | Polymorphic | 96 | ~75-100 | - |  |  |  |  |  |  |  |  |
| 80 | AS/PP_InDel-1 | GTCATACACATTCCCAAACACA  GGACTAAATGTAAGAGAACCACACG | comp27198_c0_seq19/ scaffold0000828 | alpha-1,3-mannosyl-glyco 2-beta-N-acetylglucosaminyltransferase isoform X1 | C:GO:0005802; C:GO:0005768; P:GO:0006491; F:GO:0016262; C:GO:0016021; F:GO:0003827; P:GO:0006486; P:GO:0006972 | Punjab Pink | Polymorphic | 117 | ~85-115 | 0.3297 |  |  |  |  |  |  |  |  |
| 81 | AS/PP_InDel-4 | ATCAGGGCCTTGAGTTTATCTG  GGCTCTTGTCTTGAACTTCG | comp27412_c0_seq67/ scaffold0003264 | probable disease resistance At5g66900 | F:GO:0043531; P:GO:0006952 | Punjab Pink | Non-Polymorphic | 102 | ~100-140 | - |  |  |  |  |  |  |  |  |
| 82 | AS/PP_InDel-8 | AGTTCCAGCTAACACAGGATGA  TGTTCCCTGCTATCTGTTAATTATG | comp26003_c2_seq3/ scaffold0002371 | probable receptor kinase TMK1 |  | Punjab Pink | Polymorphic | 120 | ~110-140 | 0.2149 |  |  |  |  |  |  |  |  |
| 83 | AS/PP_InDel-9 | CGAGATTTGGATCTTAGAATCG  CTGCAAATCATCCCAACGTC | comp19843_c0_seq5/ scaffold0002317 | long chain base biosynthesis 2a | F:GO:0030170; F:GO:0004758; C:GO:0016021; P:GO:0009058 | Punjab Pink | Polymorphic | 137 | ~125-150 | - |  |  |  |  |  |  |  |  |
| 84 | AS/PP_InDel-10 | TGCATTGCTATTACCTCTACTGGA  AGATAACAAGATCATCTCACTGGAC | comp26186_c1_seq71/ scaffold0002774 | E3 SUMO- ligase SIZ1-like isoform X1 |  | Punjab Pink | Polymorphic | 105 | ~85-100 | 0.3745 |  |  |  |  |  |  |  |  |
| 85 | AS/G5_InDel-1 | ATCACATCTATCCACACAATTATAGTA  TGGATTGACCTTCCAAAATAAAA | comp26859_c2_seq58/ scaffold0003032 |  |  | Apple Color | Polymorphic | 118 | ~95-125 | - |  |  |  |  |  |  |  |  |
| 86 | AS/G5_InDel-2 | ACTCTCTCTCTCCGGCCATC  CGCCTGATTAGGTTTGTTTCAG | comp24644_c0_seq6/ scaffold0000210 | E3 ubiquitin- ligase RING1-like | F:GO:0008270; F:GO:0003824; P:GO:0006511; P:GO:0016567 | Apple Color | Polymorphic | 81 | ~70-100 | - |  |  |  |  |  |  |  |  |
| 87 | AS/G5_InDel-3 | GGTGATCCAAGTCAAATCTATGTTC  TGAATCAATCTGCATCTATTCG | comp9401_c0_seq1/ scaffold0004137 |  |  | Apple Color | Polymorphic | 120 | ~80-150 | 0.2896 |  |  |  |  |  |  |  |  |
| 88 | AS/G5_InDel-4 | GTCGGTCAGTCGCTCTCCTT  CCCTGAAGTTCTTGCCAGTAAA | comp21438_c0_seq1/ scaffold0008350 | Expansin ALPHA ,EXPA4 | C:GO:0016020; P:GO:0009664; C:GO:0005576; C:GO:0009505 | Apple Color | Polymorphic | 118 | ~105-125 | 0.0830 |  |  |  |  |  |  |  |  |
| 89 | AS/G5_InDel-5 | TCAAGAAAAAGAAGTTGCAAACC  GGTCAGTCAGATCCAAGAATCA | comp24400_c0_seq3/ scaffold0003400 | E3 ubiquitin- ligase RHA1B-like | F:GO:0008270 | Apple Color | Polymorphic | 116 | ~65-100 | - |  |  |  |  |  |  |  |  |
| 90 | AS/G5_InDel-9 | GTTGCCTTTTTGGCATCTTTTA  CACTGCTTGTATTCAGGACATTTT | comp27044_c0_seq12/ scaffold0001058 | Retrovirus-related Pol poly LINE-1 | P:GO:0008152; F:GO:0004012; C:GO:0005783; F:GO:0000287; C:GO:0005789; F:GO:0000166; F:GO:0003676; P:GO:0006506; F:GO:0005524; F:GO:0046872; P:GO:0045332; F:GO:0003824; C:GO:0016020; C:GO:0016021; P:GO:0015914; P:GO:0016310; F:GO:0016740; F:GO:0016301; F:GO:0016787 | Apple Color | Polymorphic | 111 | ~95-120 | 0.3729 |  |  |  |  |  |  |  |  |
| 91 | AS/G5_InDel-11 | GCGTGAGCGTCTCTCCTCTA  CGAGCATGAAACTAGAACAAGC | comp18915_c0_seq3/ scaffold0001940 |  |  | Apple Color | Polymorphic | 84 | ~75-85 | - |  |  |  |  |  |  |  |  |
| 92 | AS/SG_InDel-1 | TTCCCATGAACTGTCTATATGGAAT  TGAATAACTGGCAATAATTGGTT | comp25568_c1_seq50/ scaffold0011631 | cyclin-dependent kinase E-1 | F:GO:0005524; F:GO:0004693; P:GO:0051726; P:GO:0006468 | L-49 | Polymorphic | 120 | ~100-125 | 0.2896 |  |  |  |  |  |  |  |  |
| 93 | AS/SG_InDel-4 | TGAACGAGATGAGAGTTTGCAT  AACTTTCACACACCCACCTACC | comp26848_c0_seq15/ scaffold0002284 | hypothetical protein EUGRSUZ_D02572 |  | L-49 | Polymorphic | 117 | ~95-110 | 0.3618 |  |  |  |  |  |  |  |  |
| 94 | AS/SG_InDel-11 | AGGACGGTGGAGAAGAGGAT  TCAACGCAAGAAGAAGAAAACC | comp21265_c0_seq20/ scaffold0001851 | abscisic acid 8 -hydroxylase 1-like | F:GO:0010295; F:GO:0005506; P:GO:0007275; C:GO:0016021; P:GO:0009687; P:GO:0016132; P:GO:0055114; P:GO:0016125; P:GO:0010268; F:GO:0020037 | L-49 | Polymorphic | 113 | ~100-125 | 0.1516 |  |  |  |  |  |  |  |  |
| 95 | AS/SG_InDel-16 | AGAGCCTCTCTGGCATTTTCAC  GCGCTTAATCGTGGATTTATTG | comp23832_c0_seq58/ scaffold0000049 | kinesin NACK1 | F:GO:0016887; F:GO:0005524; F:GO:0003777; C:GO:0005871; P:GO:0007018; C:GO:0005874; F:GO:0008017 | L-49 | Polymorphic | 113 | ~95-120 | 0.3666 |  |  |  |  |  |  |  |  |
| 96 | AS/SG_InDel-17 | CGGATCGTACTGAAACTTGTTG  CAGGAGCTTCCTGTGGAGAG | comp6349_c0_seq1/ scaffold0007501 | Glucose-fructose oxidoreductase domain-containing |  | L-49 | Polymorphic | 124 | ~105-125 | 0.2318 |  |  |  |  |  |  |  |  |
| 97 | AS/SG_InDel-19 | TCTCAGATCTGCCTTCTTTTCA  AACTGTTCCGGCATATTCTCTC | - |  |  | L-49 | Non-polymorphic |  | ~95-115 | - |  |  |  |  |  |  |  |  |
| 98 | ASgco_2380680_SSR2 | CCCCCTTTAATTAGGTGTGAT  GTAATCCCACTACTATCGCTACAGA | scaffold0004261 |  |  | Purple local | Polymorphic | 100 | ~90-100 | 0.2688 |  |  |  |  |  |  |  |  |
| 99 | ASgsc_5291_SSR5 | GCCCATCTCTTATTTCCCCTTC  TGAGCAAGAGGAGAAGCCTTG | scaffold0003179 |  |  | Purple local | Non-polymorphic | 120 | ~125 | 0.0454 |  |  |  |  |  |  |  |  |
| 100 | ASgsc_16702_SSR6 | TCGATTAACAGTGTTCCACA  CCTATGCGATTTTGGTAGATGTTCG | scaffold0002045 |  |  | Purple local | Polymorphic | 117 | ~150 | 0.2583 |  |  |  |  |  |  |  |  |
| 101 | ASgsc_16702_SSR7 | ATCACCACCCACCACCATCATC  ACGGGAGGGGAAGTAGAAGGAA | scaffold0002045 |  |  | Purple local | Polymorphic | 109 | ~125 | 0.2392 |  |  |  |  |  |  |  |  |
| 102 | ASgsc_16842_SSR8 | ATGGCGCTTTAATGCAAAGA  GGGCAGACCCTTCAATTCAT | scaffold0000968 |  |  | Purple local |  | 101 | Not amplified | - |  |  |  |  |  |  |  |  |
| 103 | ASgsc_19775_SSR11 | TTCTGGTATAAGGTCAACT  GACAACAAAATATGAAAGTTTCGAC | scaffold0002491 |  |  | Purple local | Polymorphic | 101 | ~100 | 0.1555 |  |  |  |  |  |  |  |  |
| 104 | ASgsc_23860_SSR14 | GAAGCGGGAAAAACCTCCTG  TGAGAAGGACGAGGAGGAGGAA | scaffold0017878 |  |  | Purple local | Non-polymorphic | 195 | ~150-250 | - |  |  |  |  |  |  |  |  |

**Supplementary table 4:** Single nucleotide polymorphism (SNP) - based Kompetitive Allele Specific Polymorphic (KASP) molecular markers developed equidistantly on ~150 MB guava genome distributed over 11 pseudochromosomes mapped to Eucalyptus genome - validated with Tecan-fluorescence reader (Tecan Infinite F200 Pro – KlusterCaller) after PCR amplification in 22 diverse cultivars/genotypes and related species *P. cattleianum* – strawberry & lemon guava.

| **S.No.** | **Marker_Name** | **Primer sequence**  (HEX- GAAGGTCGGAGTCAACGGATT)  (FAM- GAAGGTGACCAAGTTCATGCT) | **Allahabad Safeda Transcriptome Assembly_component_ID/ Genomic Scaffold_ID** | **Function/ Description** | **Gene Ontology_Annotation** | ***In silico* polymorphic (Genotype)** | **Fluorescence based Validation** | **KASP allele Validated (Reference/ Alternate)** | **Polymorphic**  **Information**  **Content** |
| --- | --- | --- | --- | --- | --- | --- | --- | --- | --- |
| 1 | Pg_PC02_Scaf1381_39263 | HEXgtgttccgattttgatgtgtgatA  FAMgtgttccgattttgatgtgtgatG  ggccagttgacttctgacca | comp23401_c0_seq5/ scaffold0002603 | chromatin assembly factor 1 subunit FAS1 | P:GO:0048856; P:GO:0006325; P:GO:0044767 | Purple local | Polymorphic | T/C | 0.3047 |
| 2 | Pg_PC02_C2374930_12947 | HEXccgcaccaaaattgtacagcA  FAMccgcaccaaaattgtacagcG  ttccatgccacgtgttgcta | comp13810_c0_seq2/ scaffold0006894 | aspartate-semialdehyde dehydrogenase | P:GO:0009086; P:GO:0009097; F:GO:0003942; F:GO:0050661; C:GO:0005737; F:GO:0051287; P:GO:0009089; P:GO:0009088; F:GO:0004073; F:GO:0046983; P:GO:0055114 | Apple colour | Polymorphic | T/C | 0.3484 |
| 3 | Pg_PC03_C2381408_9238 | HEXaacacaagcggagatgagaA  FAMaacacaagcggagatgagaC  ctcaactgcctcttagcccg | comp19617_c0_seq11/ scaffold0003670 | serine threonine- kinase EDR1 | F:GO:0005524; F:GO:0004674; P:GO:0006468 | Purple local  Punjab Pink | Polymorphic | T/G | 0.1239 |
| 4 | Pg_PC03_Scaf13601_16612 | HEXtcacgggtcttgctaagtcA  FAMtcacgggtcttgctaagtcG  gtggatatgtgcacgcatgt | comp27018_c2_seq17/ Scaffold0000347 | clathrin assembly At2g25430 | F:GO:0032440; P:GO:0048268; P:GO:0055114; F:GO:0030276; C:GO:0030136; F:GO:0005545 | Punjab Pink | Polymorphic | A/G | 0.3192 |
| 5 | Pg_PC04_Scaf27868_11482 | HEXgcatttggtgaattttggaccA  FAMgcatttggtgaattttggaccC  tcctcaaattcattcaccaaatgct | comp10929_c0_seq1/ Scaffold0000216 | not found | - | Purple local | Polymorphic | A/C | 0.3398 |
| 6 | Pg_PC05_C2380976_16378 | HEXagccatttcaaccattcaccT  FAMagccatttcaaccattcaccA  tccccttgttggcttctgc | comp28185_c0_seq1/ Scaffold0003874 | peroxisomal acyl-coenzyme A oxidase 1-like | F:GO:0003995; C:GO:0005777; F:GO:0003997; P:GO:0033539; F:GO:0000062; F:GO:0050660; F:GO:0009055; F:GO:0052890; P:GO:0055088 | Apple colour  L-49  Purple local  Punjab pink | Polymorphic | A/T | 0.3180 |
| 7 | Pg_PC06_Scaf14577_151441 | HEXgaggctcaatctcatatacaggG  FAMgaggctcaatctcatatacaggA  ggcaggtgggcattgagttt | comp4807_c0_seq1/Scaffold0002452 | pre-mRNA-processing-splicing factor 8 | F:GO:0000386; F:GO:0017070; F:GO:0030623; F:GO:0097157; C:GO:0071013; F:GO:0030619; C:GO:0005682; F:GO:0030620; P:GO:0000244 | Purple local | Polymorphic | G/A | 0.0866 |
| 8 | Pg_PC07_C2386872_652 | HEXtgtaacctgcatcctacacgA  FAMtgtaacctgcatcctacacgG  gcttacgtaggagcgagagt | comp26897_c2_seq29/ Scaffold0000896 | ADP-ribosylation factor GTPase-activating AGD3 | - | Apple colour  L-49  Purple local | Polymorphic | A/G | 0.1516 |
| 9 | Pg_PC07_Scaf20218_17812 | HEXcttgagcaagtagacgaagcC  FAMcttgagcaagtagacgaagcT  gtccccatcatgaccatcga | comp26024_c0_seq70/ Scaffold0002174 | nuclear pore complex NUP160 isoform X1 | - | Apple colour  L-49  Punjab pink | Polymorphic | G/A | 0.3745 |
| 10 | Pg_PC08_Scaf6377_38127 | HEXctaccactgtcgggagcaG  FAMctaccactgtcgggagcaC  ttaggctggcttcacagagc | comp3367_c0_seq1/Scaffold0001934 | hypothetical protein EUGRSUZ_J02069 | - | Purple local | Polymorphic | C/G | 0.3618 |
| 11 | Pg_PC08_Scaf9955_45208 | HEXccagctgattgaaagatctcacA  FAMccagctgattgaaagatctcacG  gagagagagtgaagaagaaggaga | comp24636_c0_seq75/ Scaffold0002297 | TIME FOR COFFEE isoform X1 | P:GO:0050896 | Purple local | Polymorphic | T/C | 0.1516 |
| 12 | Pg_PC09_C2384264_24150 | HEXcgacttgagccttaattgaagtcT  FAMcgacttgagccttaattgaagtcC  acacccaaagcagcaccat | comp4673_c0_seq1/Scaffold0002258 | floral homeotic DEFICIENS-like | C:GO:0005634; F:GO:0003677; F:GO:0003700; P:GO:0006351; P:GO:0006355; F:GO:0046983 | Purple local | Polymorphic | A/G | 0.2725 |
| 13 | Pg_PC09_Scaf28162_40238 | HEXtgcagacattatccaaggcaG  FAMtgcagacattatccaaggcaA  gggattccctccagaagctc | comp13770_c0_seq3/ Scaffold0001275 | GDSL esterase lipase At2g23540 | F:GO:0016788; F:GO:0052886; P:GO:0055114; P:GO:0052889; F:GO:0052887 | Apple colour | Polymorphic | G/A | - |
| 14 | Pg_PC10_C2376480_8038 | HEXatttagtcagagaagaaaccaccA  FAMatttagtcagagaagaaaccaccT  atctccttccttgtactctgttctg | comp25715_c0_seq38/ Scaffold0006132 | hypothetical protein LR48_Vigan05g085700 | - | Apple colour  Purple local | Polymorphic | T/A | 0.1575 |
| 15 | Pg_Comp25759_C1_S1_183_ | HEXaccgcttgttcaacgatggG  FAMaccgcttgttcaacgatggC  gaagccatccttatagctttcc | comp25759_c1_seq7 | (R,S)-reticuline 7-O-methyltransferase-like | - | - | Polymorphic | G/C | 0.3744 |
| 16 | Pg_Comp25759_C1_S1_862_ | HEXcgggcatatatcgactgaagacG  FAMcgggcatatatcgactgaagacA  tgtatgctcgatttgcgaaag | comp25759_c1_seq11 | (R,S)-reticuline 7-O-methyltransferase-like | - | - | Polymorphic | C/T | 0.0830 |
| 17 | Pg_Comp28595_Scaf28129_8277 | HEXgaggacgcgatcgaagtcA  FAMgaggacgcgatcgaagtcG  acaagagccgcatcatcgac | comp28595_c0_seq1/ Scaffold0002949 | not found | - | - | Non-polymorphic | A/G | - |
| 18 | Pg_PC01_KASP18 | HEXaaggatgatttgctgagaaaagaaA  FAMaaggatgatttgctgagaaaagaaT  cgcgtcgttattgtcatggac | comp26476_c0_seq14/ scaffold0015279 | phosphatase 1 regulatory subunit SDS22 | - | Purple local  Apple colour | Polymorphic | A/T | 0.3297 |
| 19 | Pg_PC01_KASP19 | HEXatctgcaagattcaccttcccC  FAMatctgcaagattcaccttcccT  taggcctcagcaacttcacg | comp9797_c0_seq1/ scaffold0000335 | GTP-binding SAR1A | F:GO:0005525; P:GO:0016192; C:GO:0005829; C:GO:0005783; C:GO:0005794; P:GO:0006886; C:GO:0005886 | Purple local | Polymorphic | C/T | 0.1516 |
| 20 | Pg_PC01_KASP20 | HEXggtggtcttaatactcgcacaaA  FAMggtggtcttaatactcgcacaaC  agaccaaagccgtgcctatg | comp26831_c0_seq23/ scaffold0002187 | receptor 12 | - | Purple local | Polymorphic | A/C | 0.2533 |
| 21 | Pg_PC01_KASP21 | HEXggcgttggactgccagttT  FAMggcgttggactgccagttC  ggtggatgagcatgagccg | comp21438_c0_seq2/ scaffold0002519 | Expansin ALPHA ,EXPA4 | C:GO:0016020; P:GO:0009664; C:GO:0005576; C:GO:0009505 | Purple local  Apple colour  Punjab pink  L-49 | Polymorphic | A/G | 0.2608 |
| 22 | Pg_PC01_KASP22 | HEXggtatctcagatgctctaccttctA  FAMggtatctcagatgctctaccttctG  cctcgacattttgatttggcac | comp27186_c0_seq85/ scaffold0003334 | SPP41-like isoform X1 | F:GO:0003677; C:GO:0000126; P:GO:0006359; F:GO:0001026 | Apple colour  Punjab pink  L-49 | Polymorphic | A/G | 0.1878 |
| 23 | Pg_PC01_KASP23 | HEXcctgcatatcatctttcaattcagT  FAMcctgcatatcatctttcaattcagC  acactcgagaatctggtgca | comp19146_c0_seq5/ scaffold0000636 | cyclic nucleotide-gated ion channel 1 | C:GO:0005887; F:GO:0005516; P:GO:0042391; P:GO:0071805; P:GO:0006816; F:GO:0005242; F:GO:0030552; F:GO:0005221; F:GO:0030553 | Purple local  Apple colour  Punjab pink  L-49 | Non-polymorphic | A/G | - |
| 24 | Pg_PC01_KASP24 | HEXgcagatggtttttcaaattattgcC  FAMgcagatggtttttcaaattattgcT  tacaagtaaaagcatccacttgaac | comp21973_c0_seq10/ scaffold0004346 | ATP-dependent RNA helicase DHX36 | F:GO:0003676; C:GO:0005634; F:GO:0005524; C:GO:0005737; P:GO:0006396; F:GO:0004004 | Purple local | Polymorphic | C/T | 0.0830 |
| 25 | Pg_PC01_KASP25 | HEXgctgaaaatgtcatctatccgtacT  FAMgctgaaaatgtcatctatccgtacC  acaaaaatgaagcagttggacca | comp25741_c0_seq59/ scaffold0000142 | zinc- peroxisomal-like | - | Punjab pink | Polymorphic | A/G | 0.3666 |
| 26 | Pg_PC01_KASP26 | HEXaaatcaaccaagtttgatccacA  FAMaaatcaaccaagtttgatccacG  ggtttgcgtacaaattctggaca | comp21639_c1_seq3/ scaffold0000656 | clp protease-related chloroplastic | C:GO:0009570; P:GO:0006508; C:GO:0009941; F:GO:0008233; C:GO:0009579 | Purple local | Polymorphic | A/G | 0.2078 |
| 27 | Pg_PC01_KASP116 | HEXtcaagacaccacataaaacaaacgA  FAMtcaagacaccacataaaacaaacgT  ttgacctttccctcgttgca | comp25058_c1_seq17/ scaffold0004810 | zinc finger JACKDAW | F:GO:0003676; F:GO:0003700; F:GO:0046872; P:GO:0006355 | Apple colour  Punjab Pink  L-49 | Polymorphic | A/T | - |
| 28 | Pg_PC02_KASP27 | HEXgtagcaaccatacttcctggA  FAMgtagcaaccatacttcctggG  agtagcagaaagacacggct | comp22111_c0_seq15/ scaffold0002208 | F-box family isoform 3 | - | Purple local | Polymorphic | A/G | 0.3729 |
| 29 | Pg_PC02_KASP28 | HEXaacgccggatcaccatcagT  FAMaacgccggatcaccatcagG  cggagactgaagaattgaagctg | comp19347_c0_seq3/ scaffold0000404 | myb-related 306-like | C:GO:0005634; F:GO:0003677; P:GO:0001666; P:GO:0042761 | Purple local  L-49 | Polymorphic | T/G | 0.3618 |
| 30 | Pg_PC02_KASP29 | HEXacggagaagagagagaggatT  FAMacggagaagagagagaggatC  gatgaagaggctacaccggc | comp20892_c0_seq12/ scaffold0004860 | probable galacturonosyltransferase 6 | F:GO:0047262; P:GO:0045489; C:GO:0000139; P:GO:0071555 | Purple local | Polymorphic | A/G | 0.3618 |
| 31 | Pg_PC02_KASP30 | HEXcttcaagaaatcagcagcactC  FAMcttcaagaaatcagcagcactT  ttccaactccaaagccgaag | comp19284_c0_seq9/ scaffold0007932 | nucleolar and coiled-body phospho 1-like | F:GO:0003677 | Purple local | Polymorphic | G/A | 0.2896 |
| 32 | Pg_PC02_KASP31 | HEXccttcaacccaactatatccgaatC  FAMccttcaacccaactatatccgaatG  gaggaaggtgatggaaggaca | comp22558_c0_seq4/ scaffold0002405 | pentatricopeptide repeat-containing chloroplastic-like | - | Purple local | Polymorphic | C/G | 0.3750 |
| 33 | Pg_PC02_KASP32 | HEXcagtgtagatgtgtcttaagaaagC  FAMcagtgtagatgtgtcttaagaaagA  agaatgaaattgacaatcacactca | comp19565_c0_seq1/ scaffold0000028 | serine threonine- phosphatase PP2A catalytic subunit | F:GO:0004721; P:GO:0006470 | Purple local | Polymorphic | C/A | 0.3745 |
| 34 | Pg_PC02_KASP33 | HEXtccttctacctgctcagtgT  FAMtccttctacctgctcagtgC  cggaggagggtggaagtaag | comp21563_c0_seq8/ scaffold0006758 | nucleolar complex 2 homolog | F:GO:0004601; C:GO:0030691; P:GO:0042273; C:GO:0030690; P:GO:0098869; C:GO:0005730; C:GO:0005654 | Purple local | Polymorphic | T/C | 0.3047 |
| 35 | Pg_PC02_KASP34 | HEXtcgtaactgaaaatcgatgtaaacA  FAMtcgtaactgaaaatcgatgtaaacC  gacccccgggaaatctaagtc | comp13591_c0_seq1/ scaffold0009276 | CBL-interacting serine threonine- kinase 11-like | F:GO:0005524; P:GO:0007165; P:GO:0009268; F:GO:0004674; P:GO:0006468 | Purple local | Polymorphic | T/G | 0.2078 |
| 36 | Pg_PC02_KASP117 | HEXcaacaagcaagccttatagggC  FAMcaacaagcaagccttatagggA  acgttcatcgattctttcattgt | comp25827_c0_seq37/ scaffold0001778 | TBC1 domain family member 13-like |  | Purple Local | Polymorphic | C/A | - |
| 37 | Pg_PC02_KASP118 | HEXggatcgaaacccgcgtcA  FAMggatcgaaacccgcgtcG  ggtacgtgtcgttcgatggg | comp28413_c0_seq1/ scaffold0016636 | hypothetical protein EUGRSUZ_B03018 |  | Apple colour | Polymorphic | T/C | - |
| 38 | Pg_PC03_KASP35 | HEXactccaatttgctgtgtatctctG  FAMTactccaatttgctgtgtatctctA  cagaaggtggttcttgccct | comp23590_c0_seq8/ scaffold0000774 | microtubule-associated TORTIFOLIA1 | C:GO:0005874 | Purple local | Polymorphic | C/T | 0.1239 |
| 39 | Pg_PC03_KASP36 | HEXctgggaatctgctctccgT  FAMctgggaatctgctctccgG  gtctgcccagagcttgtagc | comp20053_c0_seq8/ scaffold0001897 | coat | C:GO:0019013; C:GO:0019028 | Purple local | Polymorphic | T/G | 0.2318 |
| 40 | Pg_PC03_KASP37 | HEXtctgcagaattttctctgaccA  FAMtctgcagaattttctctgaccG  cagacgaggaggaggaggag | comp18309_c0_seq12/ scaffold0003919 | RNA polymerase-associated CTR9 homolog | P:GO:0045893; C:GO:0016020; C:GO:0016593; P:GO:0016571; P:GO:0051569; F:GO:0000993; P:GO:0009910 | Purple local | Polymorphic | A/G | 0.3398 |
| 41 | Pg_PC03_KASP38 | HEXggagtatttctttctgcggtatagT  FAMggagtatttctttctgcggtatagC  ttggccaatgagacggaact | comp24709_c0_seq27/ scaffold0000943 | F-box kelch-repeat At1g55270-like | F:GO:0004842; C:GO:0031463; P:GO:0016567 | Purple local | Polymorphic | T/C | 0.1812 |
| 42 | Pg_PC03_KASP39 | HEXtctccatttcaagcttcggT  FAMtctccatttcaagcttcggC  agaaaaagctccatcgcccc | comp58481_c0_seq1/ scaffold0000184 | probable glycosyltransferase At5g25310 | - | L-49 | Non-polymorphic | T/C | - |
| 43 | Pg_PC03_KASP40 | HEXtgctctgcttttccgactatatcG  FAMtgctctgcttttccgactatatcA  ccggagagaagggttccaga | comp19679_c0_seq1/ scaffold0012657 | F-box At3g07870-like | F:GO:0004842; P:GO:0031146; C:GO:0019005; P:GO:0016567 | L-49 | Non-Polymorphic | C/T | - |
| 44 | Pg_PC03_KASP41 | HEXatccggcggaataactcccA  FAMatccggcggaataactcccG  aattacagaaggccttcagagg | comp25688_c0_seq64/ scaffold0006316 | lactation elevated 1 | - | Apple colour  Purple local | Polymorphic | T/C | 0.3685 |
| 45 | Pg_PC03_KASP42 | HEXtggagaagttgatggaagagcaC  FAMtggagaagttgatggaagagcaT  tttctttcctccttggccagc | comp27540_c0_seq61/ scaffold0020679 | 1-phosphatidylinositol-3-phosphate 5-kinase FAB1A isoform X1 | F:GO:0005524; F:GO:0016307; F:GO:0046872; P:GO:0016043; P:GO:0046854 | Purple local | Non-polymorphic | G/A | - |
| 46 | Pg_PC03_KASP43 | HEXcagttacatgggatttgctgcC  FAMcagttacatgggatttgctgcT  cttccttcacttgagcgacg | comp19295_c0_seq5/ scaffold0001649 | PXMP2 4 family 2 | C:GO:0016021 | Purple local | Polymorphic | C/T | 0.2725 |
| 47 | Pg_PC03_KASP44 | HEXggaacaagaagagaggataccaaT  FAMggaacaagaagagaggataccaaC  gacgactgcaccatcttcca | comp16922_c0_seq2/ scaffold0001659 | calmodulin-binding 60-D | F:GO:0005516; P:GO:0006950 | Apple colour | Polymorphic | A/G | 0.3557 |
| 48 | Pg_PC03_KASP119 | HEXtccgctgtatctataagttcatgaT  FAMtccgctgtatctataagttcatgaC  tcccccatcaagctggctat | comp23422_c0_seq30/ scaffold0039085 | DEAD-box ATP-dependent RNA helicase 56 isoform X1 | F:GO:0003676; F:GO:0005524; F:GO:0004386 | Purple Local | Non-polymorphic | T/C | - |
| 49 | Pg_PC04_KASP45 | HEXgagaaggatgttgaggcccG  FAMgagaaggatgttgaggcccC  accacagttccctctccaga | comp20622_c0_seq12/ scaffold0003396 | plasma membrane-associated cation-binding 1 | P:GO:0051716; C:GO:0046658 | Apple colour  Purple local  L-49  Punjab pink | Polymorphic | G/C | 0.3457 |
| 50 | Pg_PC04_KASP46 | HEXggggctcactataggaatccA  FAMggggctcactataggaatccG  tccttcataaacaagcctaagcg | comp19313_c0_seq12/ scaffold0005037 | transcription factor GTE12 | F:GO:0003743; C:GO:0005737; F:GO:0016740; P:GO:0006413 | L-49  Punjab pink | Polymorphic | A/G | 0.3398 |
| 51 | Pg_PC04_KASP47 | HEXcgtaaggtgtgatagcgtcG  FAMcgtaaggtgtgatagcgtcA  gtttccgagctttattatgttagct | comp22866_c0_seq3/ scaffold0000002 | fiber Fb34 | C:GO:0016021 | L-49  Punjab pink  Purple local | Polymorphic | C/T | 0.3727 |
| 52 | Pg_PC04_KASP48 | HEXcacatgacgcgtcggctatA  FAMcacatgacgcgtcggctatC  tctttgtctgattgctgcga | comp27403_c0_seq78/ scaffold0000685 | RING finger and CHY zinc finger domain-containing 1 | F:GO:0008270 | Apple colour | Polymorphic | T/G | 0.2970 |
| 53 | Pg_PC04_KASP49 | HEXtagctcagaaatcgcggaaT  FAMtagctcagaaatcgcggaaC  gcgatcgcttttgagctcaa | comp18385_c0_seq6/ scaffold0000842 | nucleolin isoform X1 | F:GO:0000166; F:GO:0003676; C:GO:0030529; C:GO:0019013 | Purple local | Non-polymorphic | T/C | - |
| 54 | Pg_PC04_KASP50 | HEXtccctcctttatttgcaagagaC  FAMtccctcctttatttgcaagagaA  aaatggtgcacggggaagag | comp17067_c0_seq8/ scaffold0002341 | probable serine threonine- kinase clkA | - | L-49  Punjab pink  Purple local | Polymorphic | C/A | 0.3729 |
| 55 | Pg_PC04_KASP51 | HEXaagccattgccacatgcctA  FAMaagccattgccacatgcctG  gtctgggtctattgaaatggaca | comp23164_c0_seq17/ scaffold0014117 | Tryptophan RNA-binding attenuator -like | C:GO:0016021; F:GO:0016853 | Purple local | Polymorphic | T/C | 0.2149 |
| 56 | Pg_PC04_KASP52 | HEXgcagatcccactaaatgaaacgT  FAMgcagatcccactaaatgaaacgC  tcatgtgagcacgggtactg | comp9333_c0_seq1/ scaffold0000129 | Embryo defective isoform 1 | P:GO:0009651; C:GO:0016021; C:GO:0005829; P:GO:0046777; P:GO:0046854; F:GO:0004430; F:GO:0004674 | Purple local | Polymorphic | T/C | - |
| 57 | Pg_PC04_KASP53 | HEXcgctcttgatggaaaccaaagT  FAMcgctcttgatggaaaccaaagC  ccaggtcttccatgacgacc | comp8480_c0_seq1/ scaffold0002980 | AAA-type ATPase family | F:GO:0005524; C:GO:0016021; F:GO:0016787 | Purple local | Polymorphic | A/G | 0.1878 |
| 58 | Pg_PC04_KASP120 | HEXaatttgatgcctaaatacaccacaG  FAMaatttgatgcctaaatacaccacaT  cctttgttcagttgttttcggc | comp22609_c0_seq14/ scaffold0007092 | polyadenylate-binding RBP45B isoform X3 | F:GO:0000166; F:GO:0003723; P:GO:0009735 | Apple colour | Non-polymorphic | C/A | - |
| 59 | Pg_PC05_KASP54 | HEXgagactagtagtggcggtcT  FAMgagactagtagtggcggtcC  gctgaagaaggtgcgctaga | comp13366_c0_seq3/ scaffold0013163 | F-box family isoform 1 | F:GO:0003677; P:GO:0006355 | Purple local | Polymorphic | T/C | 0.3618 |
| 60 | Pg_PC05_KASP56 | HEXtaggtcacaagacggacggC  FAMtaggtcacaagacggacggT  ttgaattcctcactcggcga | comp22924_c0_seq31/ scaffold0001438 | PREDICTED: uncharacterized protein LOC104443934 | C:GO:0016020; C:GO:0016021 | Purple local | Polymorphic | C/T | 0.3297 |
| 61 | Pg_PC05_KASP57 | HEXggatgatagagaaggtggatgcC  FAMggatgatagagaaggtggatgcA  gcaagtgcgattcttgatggt | comp27477_c0_seq182/ scaffold0006479 | nucleotide binding | C:GO:0012505; C:GO:0019898; F:GO:0005543 | Purple local | Polymorphic | C/A | 0.3666 |
| 62 | Pg_PC05_KASP58 | HEXcttcctggtcaaaagcacgaatC  FAMcttcctggtcaaaagcacgaatT  tgcagaatcactagcaggagc | comp18822_c0_seq2/ scaffold0000194 | ABC transporter C family member 15 | F:GO:0005524; F:GO:0042626; P:GO:0055085; C:GO:0016021 | L-49 | Polymorphic | C/T | 0.3729 |
| 63 | Pg_PC05_KASP59 | HEXactccctctcgtcctgcA  FAMactccctctcgtcctgcG  ttgttggagaggcgaacgag | comp24837_c0_seq3/ scaffold0014133 | cytochrome P450 71A1-like | F:GO:0016491; F:GO:0046872; C:GO:0016021 | L-49 | Polymorphic | A/G | 0.2533 |
| 64 | Pg_PC05_KASP60 | HEXgctcttcactccgtgttacaG  FAMgctcttcactccgtgttacaC  gacaacgcggattcttgaagt | comp25259_c0_seq140/ scaffold0002088 | TMV resistance N-like | P:GO:0044699; P:GO:0009987; P:GO:0050896 | Purple local | Polymorphic | G/C | 0.3297 |
| 65 | Pg_PC05_KASP61 | HEXcgaagaagctgagctcctcC  FAMcgaagaagctgagctcctcT  ccgacggggaactgcagg | comp14122_c0_seq2/ scaffold0006338 | At4g21310p | C:GO:0016021 | Purple local | Polymorphic | C/T | 0.2318 |
| 66 | Pg_PC05_KASP62 | HEXtgttcatttggcctgcatcA  FAMtgttcatttggcctgcatcG  gcccatatgagaaaggatgga | comp19297_c0_seq12/ scaffold0004540 | glycine cleavage system H mitochondrial | P:GO:0019464; C:GO:0005960 | L-49 | Polymorphic | T/C | 0.3180 |
| 67 | Pg_PC05_KASP121 | HEXccacggttacacatcgccaT  FAMccacggttacacatcgccaC  cttcaggatctgtctaatgtagga | comp25528_c0_seq73/ scaffold0006739 | E3 ubiquitin- ligase BRE1-like 1 | P:GO:0010162; F:GO:0008270; F:GO:0042803; P:GO:0010390; P:GO:0009965; P:GO:0009817; P:GO:0051301; C:GO:0005634; F:GO:0004842; C:GO:0005739; P:GO:0010228; F:GO:0016874; P:GO:0010389; P:GO:0016049; P:GO:0033523 | Purple local  Apple colour | Polymorphic | A/G | - |
| 68 | Pg_PC06_KASP63 | HEXttgacccggcccaaggaC  FAMttgacccggcccaaggaT  tggatggataggggcgagaa | comp19000_c1_seq1/ scaffold0000604 | serpin-ZX-like | C:GO:0005615; P:GO:0006508; F:GO:0008233; F:GO:0016740 | Purple local | Polymorphic | G/A | 0.3297 |
| 69 | Pg_PC06_KASP64 | HEXcatcgccgggtgaggtaaC  FAMcatcgccgggtgaggtaaT  ccccgttctgaccgaatca | comp26322_c0_seq35/ scaffold0001343 | FRIGIDA 1 |  | Apple colour | Polymorphic | C/T | 0.3750 |
| 70 | Pg_PC06_KASP65 | HEXtctctctgtttctctgatgcaaaA  FAMtctctctgtttctctgatgcaaaT  gactgacaagaagcagcagc | comp13917_c0_seq2/ scaffold0004441 | #NAME? | P:GO:0006605; F:GO:0015450; P:GO:0065002; C:GO:0016020; P:GO:0015031; C:GO:0016021; P:GO:0043952; P:GO:0006886; P:GO:0006810; C:GO:0005622; C:GO:0005886 | Purple local | Polymorphic | A/T | 0.1812 |
| 71 | Pg_PC06_KASP66 | HEXtcacccttccaagttgagtaaT  FAMtcacccttccaagttgagtaaA  tcagcactcatgagatcacct | comp28391_c0_seq1/ scaffold0007922 | PREDICTED: uncharacterized protein At4g26450 | - | Purple local | Polymorphic | T/A | 0.3047 |
| 72 | Pg_PC06_KASP67 | HEXgtcttgcatgatcccattcgA  FAMgtcttgcatgatcccattcgG  gcactctcaccaaaatctgca | comp13428_c0_seq3/ scaffold0005130 | probable alkaline neutral invertase D | F:GO:0033926; P:GO:0008152; F:GO:0004575 | Purple local | Polymorphic | A/G | 0.3618 |
| 73 | Pg_PC06_KASP68 | HEXtcttgaacaacggaaagctacttT  FAMtcttgaacaacggaaagctacttC  tcatctgctatgctgcaagaga | comp23334_c1_seq4/ scaffold0000662 | adenylate chloroplastic | C:GO:0009570; F:GO:0004017; F:GO:0005524; P:GO:0046939; P:GO:2000505 | Purple local  Apple colour | Polymorphic | T/C | - |
| 74 | Pg_PC06_KASP69 | HEXtgaatgtctcactatcaaagcgtG  FAMTtgaatgtctcactatcaaagcgtA  gggaatctcctatctcaccct | comp13795_c0_seq2/ scaffold0000358 | scarecrow 21 | C:GO:0005634; F:GO:0003700; P:GO:0006351; P:GO:0006355; F:GO:0043565 | Purple local  Apple colour | Polymorphic | G/A | 0.2896 |
| 75 | Pg_PC06_KASP70 | HEXccaaaaaggagctcatgccG  FAMTccaaaaaggagctcatgccA  ctctgcctcagaactggtct | comp24692_c0_seq39/ scaffold0001118 | clustered mitochondria homolog | F:GO:0003743; C:GO:0005829; P:GO:0006413 | Purple local | Polymorphic | C/T | 0.3047 |
| 76 | Pg_PC06_KASP71 | HEXgatgccgctgtccctgcA  FAMTgatgccgctgtccctgcG  gacccgtgagtgatgaagaca | comp25749_c1_seq24/ scaffold0008755 | peptidyl-tRNA hydrolase mitochondrial | - | Purple local | Polymorphic | T/C | 0.3618 |
| 77 | Pg_PC06_KASP122 | HEXccattcccatcagcaggagA  FAMccattcccatcagcaggagG  taatgttgtcggggtggcac | comp29202_c0_seq1/ scaffold0003260 | probable BOI-related E3 ubiquitin- ligase 2 | C:GO:0005634; F:GO:0004842; F:GO:0008270; P:GO:0031347; P:GO:0016567 | Purple local | Non-polymorphic | A/G | - |
| 78 | Pg_PC06_KASP123 | HEXgtcgaatacacccaactcatgT  FAMgtcgaatacacccaactcatgC  gaacgaccgtcagctgagag | comp27202_c5_seq2/ scaffold0011036 | double-stranded RNA-binding 1-like | F:GO:0035198; P:GO:0090502; P:GO:0031053; P:GO:0031054; P:GO:0009735; P:GO:0009733; F:GO:0004525; P:GO:0009737; F:GO:0003725; P:GO:0010589; C:GO:0010445; P:GO:0010305; P:GO:0035279; P:GO:0010267 | Purple local | Polymorphic | T/C | - |
| 79 | Pg_PC07_KASP72 | HEXcccatatactacagtggagcgaC  FAMcccatatactacagtggagcgaT  tggtcaactgctcaaggtgg | comp26024_c0_seq70/ scaffold0002174 | nuclear pore complex NUP160 isoform X1 | - | L-49 | Polymorphic | G/A | 0.2896 |
| 80 | Pg_PC07_KASP73 | HEXctctccctgttcttcccctG  FAMctctccctgttcttcccctT  ggcttctcaactcttcgcca | comp29105_c0_seq1/ scaffold0001159 | probable beta-D-xylosidase 5 isoform X2 | - | L-49 | Polymorphic | C/A | 0.2078 |
| 81 | Pg_PC07_KASP74 | HEXcctgggacactgaattagcgA  FAMcctgggacactgaattagcgG  acaatttaatgcagcaattgttgt | comp25105_c0_seq7/ scaffold0000270 | DNA ligase 1-like | P:GO:0051103; F:GO:0003677; F:GO:0005524; F:GO:0003910; P:GO:0006260; P:GO:0006310; P:GO:0071897 | Purple local | Polymorphic | A/G | 0.3398 |
| 82 | Pg_PC07_KASP75 | HEXgaaccctgtcaattatccccgA  FAMgaaccctgtcaattatccccgT  tcttctccaggaaagccaga | comp28206_c0_seq1/ scaffold0003921 | alpha-xylosidase 1 | P:GO:0000023; F:GO:0004558; F:GO:0032450; F:GO:0030246 | Purple local  Apple colour | Polymorphic | T/A | 0.3648 |
| 83 | Pg_PC07_KASP76 | HEXcaactgaaggttttctgagagccA  FAMcaactgaaggttttctgagagccG  cggagccagacctagcagta | comp26052_c0_seq90/ scaffold0004447 | U-box domain-containing 14 | - | Apple colour | Polymorphic | A/G | 0.3618 |
| 84 | Pg_PC07_KASP77 | HEXcgttattggttttcgattggtctG  FAMcgttattggttttcgattggtctT  ggaggaggaggaggagatga | comp10412_c0_seq2/ scaffold0008867 | coronatine-insensitive 1 | - | Apple colour  Purple local | Polymorphic | G/T | 0.3180 |
| 85 | Pg_PC07_KASP78 | HEXacttttaacttgttcaggtttgtgG  FAMacttttaacttgttcaggtttgtgA  ttaagacacgaccagattgaca | comp26845_c1_seq47/ scaffold0001044 | cycloartenol-C-24-methyltransferase | - | Purple local | Polymorphic | G/A | 0.3180 |
| 86 | Pg_PC07_KASP124 | HEXactttcctctatacttcggctttG  FAMactttcctctatacttcggctttT  agggcgacaccaaagttagt | comp22393_c0_seq19/ scaffold0005836 | tRNA (guanine(10)-N2)-methyltransferase homolog | F:GO:0003676; F:GO:0008168; P:GO:0006400; P:GO:0032259; P:GO:0080180 | Purple local | Non-polymorphic | C/A | - |
| 87 | Pg_PC07_KASP125 | HEXctcacatgtgcaagaccgaC  FAMctcacatgtgcaagaccgaT  atgggtccaattgcgagctg | comp21087_c0_seq7/ scaffold0011302 | ubiquitin-conjugating enzyme E2 28-like | F:GO:0005524; F:GO:0016874 | Purple local  Apple colour | Polymorphic | G/A | - |
| 88 | Pg_PC08_KASP79 | HEXgatccaatcaatttgctcacagT  FAMgatccaatcaatttgctcacagC  agctgtgctgagattggaca | comp21422_c0_seq9/ scaffold0001420 | chaperone chloroplastic | C:GO:0009570; F:GO:0016887; F:GO:0005524; P:GO:0019538; F:GO:0042803; C:GO:0009941; P:GO:0034214 | Purple local | Polymorphic | A/G | 0.1575 |
| 89 | Pg_PC08_KASP80 | HEXggagaagttgatgaagggtacT  FAMggagaagttgatgaagggtacC  gcagacctccggaccatatc | comp27181_c4_seq5/ scaffold0002737 | NF-X1-type zinc finger NFXL1 | F:GO:0008270; P:GO:0009651; F:GO:0000977; P:GO:0006366; P:GO:0009642; P:GO:0009697; P:GO:0042742; F:GO:0000166; P:GO:0000122; C:GO:0005634; F:GO:0001078; P:GO:0010310; P:GO:0010188 | Purple local | Polymorphic | T/C | 0.3557 |
| 90 | Pg_PC08_KASP81 | HEXtgttcaaagcgttccgtgcT  FAMtgttcaaagcgttccgtgcC  agcacataatttgaggcccca | comp25505_c0_seq27/ scaffold0005678 | bromodomain and WD repeat-containing 3 | P:GO:0050794 | Purple local | Polymorphic | T/C | 0.3618 |
| 91 | Pg_PC08_KASP82 | HEXtgcttagacttgcatgtgacttT  FAMtgcttagacttgcatgtgacttG  tgcttgttttccgcatcttaaga | comp27817_c0_seq1/ scaffold0006679 | Leucine-rich repeat family | P:GO:0007165; F:GO:0019199; C:GO:0016021; P:GO:0006468 | Purple local | Polymorphic | A/C | 0.3297 |
| 92 | Pg_PC08_KASP83 | HEXcgcgttgaaggcccagttaC  FAMcgcgttgaaggcccagttaA  tgctgggtctctccgattct | comp26375_c0_seq67/ scaffold0001990 | ubiquitin-like-specific protease 1D isoform X2 | - | Purple local | Polymorphic | G/T | 0.1516 |
| 93 | Pg_PC08_KASP84 | HEXttctatgttttcttttggccatcA  FAMttctatgttttcttttggccatcG  accaacagttctgctacgga | comp27792_c0_seq1/ scaffold0010352 | malate dehydrogenase | P:GO:0005975; F:GO:0030060; P:GO:0006108; C:GO:0005737; P:GO:0006099 | L-49 | Polymorphic | T/C | 0.3297 |
| 94 | Pg_PC08_KASP85 | HEXaaccaatcaaaccgaaataaaggC  FAMaaccaatcaaaccgaaataaaggT  tggaaactgggatgagctga | comp14242_c0_seq3/ scaffold0002852 | chromatin-remodelling complex ATPase chain | F:GO:0016887; F:GO:0003677; F:GO:0005524; F:GO:0031491; P:GO:0043044; F:GO:0004386; F:GO:0016740; C:GO:0016589 | Purple local | Polymorphic | G/A | 0.2533 |
| 95 | Pg_PC08_KASP86 | HEXccatttgcgcccactagcA  FAMccatttgcgcccactagcT  aagaagcaagccgacgaaga | comp28429_c0_seq1/ scaffold0000758 | probable nucleolar 5-2 | C:GO:0031428; C:GO:0032040; P:GO:0000154; F:GO:0030515 | Apple colour | Polymorphic | A/T | 0.3557 |
| 96 | Pg_PC08_KASP87 | HEXacctcagctcttgaacgcC  FAMacctcagctcttgaacgcG  ggggtcagaagcagaaagaga | comp2733_c0_seq1/ scaffold0002545 | transcription factor bHLH94-like | F:GO:0046983 | L-49 | Polymorphic | G/C | 0.3047 |
| 97 | Pg_PC08_KASP88 | HEXagactcccattccaccaaaaataC  FAMagactcccattccaccaaaaataT  caatgctgtgttgctggcaa | comp5196_c0_seq2/ scaffold0004844 | auxilin-related 2-like | - | Apple colour  Purple local | Polymorphic | G/A | 0.3484 |
| 98 | Pg_PC08_KASP126 | HEXgcaagtttcttcctttttgcgC  FAMgcaagtttcttcctttttgcgG  gaagcagaaggagagcgaca | comp4911_c0_seq1/ scaffold0000201 | small EDRK-rich factor 2 |  | Purple local | Non-polymorphic | G/C | - |
| 99 | Pg_PC08_KASP127 | HEXactcaagttctcattccactcA  FAMactcaagttctcattccactcG  actgggtttggcaatcacct | comp28014_c0_seq1/ scaffold0006059 | proteasome subunit beta type-4-like | C:GO:0005634; C:GO:0022626; C:GO:0005839; F:GO:0004298; P:GO:0051603 | Purple local  Apple colour | Polymorphic | A/G | - |
| 100 | Pg_PC09_KASP89 | HEXatgcacctcctgtcaggttT  FAMatgcacctcctgtcaggttG  tgcagatcacactagcagatgt | comp17606_c0_seq4/ scaffold0000638 | probable phosphoribosylformylglycinamidine chloroplastic mitochondrial | F:GO:0005524; C:GO:0005737; P:GO:0006189; P:GO:0006541; F:GO:0004642 | Apple colour  Purple local | Polymorphic | A/C | 0.3538 |
| 101 | Pg_PC09_KASP90 | HEXagtcaacctcactaactaatgcG  FAMagtcaacctcactaactaatgcA  catttggatctcatgtgttttgct | comp13287_c0_seq2/ scaffold0002092 | PREDICTED: uncharacterized protein At4g37920, chloroplastic | C:GO:0009941; C:GO:0009535 | Purple local | Polymorphic | C/T | 0.3727 |
| 102 | Pg_PC09_KASP91 | HEXaccagcaccaccaccagT  FAMaccagcaccaccaccagC  tctgctggatcggggtgg | comp13374_c0_seq3/ scaffold0002090 | WRKY DNA-binding 7 isoform 1 | F:GO:0003700; P:GO:0006355; F:GO:0043565 | Purple local | Polymorphic | T/C | 0.3047 |
| 103 | Pg_PC09_KASP92 | HEXagatatcgacaaggctaagctcG  FAMagatatcgacaaggctaagctcA  gccacttgagccttacatcg | comp9564_c0_seq2/ scaffold0003705 | ferredoxin--nitrite chloroplastic | C:GO:0009570; C:GO:0005739; C:GO:0016020; C:GO:0048046; P:GO:0055114; F:GO:0051536; F:GO:0020037; F:GO:0048307 | Purple local | Polymorphic | C/T | 0.1516 |
| 104 | Pg_PC09_KASP93 | HEXcgttactggaacatcatcacgC  FAMcgttactggaacatcatcacgT  ttcgagctaccacttcgtcg | comp23510_c0_seq39/ scaffold0000424 | E3 ubiquitin- ligase UPL3 | C:GO:0005634; F:GO:0004842; C:GO:0005737; P:GO:0042023; P:GO:0010091; C:GO:0005886; F:GO:0016874; P:GO:0042787 | Purple local | Polymorphic | C/T | 0.2896 |
| 105 | Pg_PC09_KASP94 | HEXtcttgtcatggtttgctgcG  FAMtcttgtcatggtttgctgcC  acaaagacggaaggaaacaga | comp28669_c0_seq1/ scaffold0010707 | cinnamyl alcohol dehydrogenase | F:GO:0008270; F:GO:0045551; P:GO:0009809; P:GO:0055114; F:GO:0052747 | Purple local | Polymorphic | G/C | 0.3618 |
| 106 | Pg_PC09_KASP95 | HEXctctaattcaagccgtcgcA  FAMctctaattcaagccgtcgcG  aagtcgccagccatcgtc | comp19641_c0_seq15/ scaffold0000005 | uricase-2 isozyme 2 | C:GO:0005777; P:GO:0019628; F:GO:0004846; P:GO:0007031; P:GO:0055114; P:GO:0006144 | Purple local | Polymorphic | A/G | 0.1190 |
| 107 | Pg_PC09_KASP96 | HEXacaggaacaagcacatgaagC  FAMacaggaacaagcacatgaagT  ctccacgatcccttcttggt | comp22809_c0_seq9/ scaffold0005389 | la-related 1C | P:GO:0009737; C:GO:0005737; P:GO:0010150; P:GO:0009753; P:GO:0009751 | Purple local | Polymorphic | C/T | 0.3047 |
| 108 | Pg_PC09_KASP97 | HEXtctcaaagttcaggagcgtcaG  FAMtctcaaagttcaggagcgtcaA  caatggcaacggttgtgcat | comp17847_c0_seq3/ scaffold0009400 | splicing factor 3B subunit 1 | C:GO:0005689; C:GO:0009507; C:GO:0071013; F:GO:0003729; C:GO:0071004; C:GO:0005686; P:GO:0000245 | Punjab pink | Polymorphic | C/T | 0.2318 |
| 109 | Pg_PC09_KASP98 | HEXtgctgcgtcttcttctaggaaT  FAMtgctgcgtcttcttctaggaaG  gctccctcaactatggtggg | comp29045_c0_seq1/ scaffold0012291 | glucuronoxylan 4-O-methyltransferase 1 | P:GO:0045492; C:GO:0016021; F:GO:0008168; P:GO:0032259 | Purple local | Polymorphic | T/G | 0.3618 |
| 110 | Pg_PC09_KASP128 | HEXgccacttctatttcctggcC  FAMgccacttctatttcctggcT  gtcctagcaagcatggccat | comp13144_c0_seq2/ scaffold0002546 | kinesin light chain | P:GO:0031347; C:GO:0005886 | L-49 | Non-polymorphic | C/T | - |
| 111 | Pg_PC10_KASP99 | HEXcttcatcttcatcaatactgttccA  FAMcttcatcttcatcaatactgttccG  tacagggctatgcaaggttga | comp23525_c0_seq16/ scaffold0001381 | SNF2 domain-containing CLASSY 1-like | F:GO:0005524 | Purple local | Polymorphic | T/C | 0.0866 |
| 112 | Pg_PC10_KASP100 | HEXacataacatagttggatgcgctT  FAMacataacatagttggatgcgctC  tgacgtcaccctccaagttt | comp27201_c1_seq149/ scaffold0006535 | AP-2 complex subunit alpha-1-like | F:GO:0008270; P:GO:0016192; C:GO:0030131; F:GO:0008565; P:GO:0006886 | Apple colour  L-49 | Polymorphic | A/G | 0.3047 |
| 113 | Pg_PC10_KASP101 | HEXggaacttcggctgtctgcG  FAMggaacttcggctgtctgcT  cggaacacgtcactattggg | comp22591_c0_seq10/ scaffold0018597 | NAD(H) kinase 1 | F:GO:0003951; F:GO:0005516; F:GO:0042736; C:GO:0005737; P:GO:0019674; P:GO:0016310; P:GO:0006741 | Purple local | Polymorphic | C/A | 0.2078 |
| 114 | Pg_PC10_KASP102 | HEXtcgtgatgggaaaatctgacC  FAMtcgtgatgggaaaatctgacA  cggattctcttctctacattggga | comp14356_c0_seq2/ scaffold0003072 | pumilio homolog 4 isoform X1 | F:GO:0003723 | Purple local | Polymorphic | G/T | 0.3524 |
| 115 | Pg_PC10_KASP103 | HEXaatcttaagatctccaattcccaaC  FAMaatcttaagatctccaattcccaaA  tgtgtactgatgatgctgagtt | comp5086_c0_seq2/ scaffold0006292 | IQ-DOMAIN 31-like | - | Purple local | Polymorphic | C/A | 0.2533 |
| 116 | Pg_PC10_KASP104 | HEXgtcaccaccacgtcgtgC  FAMTgtcaccaccacgtcgtgT  caagcacctcctcgccca | comp13739_c0_seq2/ scaffold0000883 | NUCLEAR FUSION DEFECTIVE 4-like | C:GO:0016021 | Apple colour  Purple local | Polymorphic | G/A | 0.3729 |
| 117 | Pg_PC10_KASP105 | HEXgttccattttcaggcggtcG  FAMgttccattttcaggcggtcA  gaagcctttcaactgcaatctcc | comp4842_c1_seq1/ scaffold0003511 | formate mitochondrial | P:GO:0042183; F:GO:0051287; C:GO:0005739; F:GO:0004617; F:GO:0008863; P:GO:0055114; P:GO:0009070 | Purple local | Polymorphic | C/T | 0.2725 |
| 118 | Pg_PC10_KASP106 | HEXtgtccctttactccggtgaG  FAMtgtccctttactccggtgaC  ggcatagaccacgggaagtt | comp29078_c0_seq1/ scaffold0006354 | subtilisin-like protease | C:GO:0048046; P:GO:0006508; F:GO:0004252; C:GO:0005774; C:GO:0009505; P:GO:0010223 | Punjab pink | Polymorphic | G/C | 0.3557 |
| 119 | Pg_PC10_KASP107 | HEXgccgctgtacaaatagtgttcC  FAMgccgctgtacaaatagtgttcT  ggggagcaggtggtttcag | comp13187_c0_seq4/ scaffold0002047 | pentatricopeptide repeat-containing mitochondrial | - | Purple local | Polymorphic | C/T | 0.3180 |
| 120 | Pg_PC10_KASP129 | HEXgtgaaggattatggcaaatgaagtC  FAMgtgaaggattatggcaaatgaagtA  cccactctctcatccatatttgact | comp25103_c0_seq75/ scaffold0000541 | isoprenylcysteine alpha-carbonyl methylesterase ICME-like | C:GO:0005789; F:GO:0004061; P:GO:0008152; C:GO:0016021; C:GO:0000139 | Purple local | Non-polymorphic | G/T | - |
| 121 | Pg_PC11_KASP108 | HEXcagattgtgaagaaggggcA  FAMcagattgtgaagaaggggcG  tcgccatcacatactagctct | comp14341_c0_seq3/ scaffold0003070 | phosphatidylinositol 4-kinase gamma 3 | C:GO:0005777; P:GO:0016310; F:GO:0016301 | Purple local | Polymorphic | A/G | 0.2533 |
| 122 | Pg_PC11_KASP109 | HEXttgtggttcagcgagctcT  FAMttgtggttcagcgagctcA  tcttgcatcgagtacacgcc | comp23729_c0_seq21/ scaffold0000808 | PREDICTED: uncharacterized protein LOC104424983 | - | Purple local | Polymorphic | T/A | - |
| 123 | Pg_PC11_KASP110 | HEXgcttggcgtcgaaacaactagC  FAMgcttggcgtcgaaacaactagA  tttttcctttgttgaggcgct | comp25826_c1_seq18/ scaffold0000479 | zinc finger CCCH domain-containing 14 | - | Purple local | Polymorphic | G/T | 0.3750 |
| 124 | Pg_PC11_KASP111 | HEXaagcagccaaatccgtcgtC  FAMaagcagccaaatccgtcgtT  tgggatatggtctcattcttgtct | comp17520_c1_seq3/ scaffold0004485 | U-box domain-containing 9 | F:GO:0004842; P:GO:0016567 | Purple local | Polymorphic | G/A | 0.3730 |
| 125 | Pg_PC11_KASP112 | HEXacatcttgatcgaggttgccG  FAMacatcttgatcgaggttgccA  ttgtctcgacactaaagttgggt | comp26661_c0_seq25/ scaffold0016259 | RPA-interacting B | - | Purple local | Polymorphic | C/T | 0.3398 |
| 126 | Pg_PC11_KASP113 | HEXtgttctaaatggaaaaccttgcA  FAMtgttctaaatggaaaaccttgcG  tgaagcgaacaagaattgtcct | comp26367_c2_seq175/ scaffold0011345 | FAD synthase | - | Purple local  L-49 | Polymorphic | T/C | 0.3484 |
| 127 | Pg_PC11_KASP114 | HEXatcaccagaagacccatccT  FAMatcaccagaagacccatccC  ggctcctaggtgtgtgctac | comp13988_c0_seq4/ scaffold0002171 | cysteine protease ATG4 isoform X1 | P:GO:0000045; P:GO:0051697; P:GO:0044804; F:GO:0004197; C:GO:0005829; P:GO:0016485; P:GO:0006501; P:GO:0000422; P:GO:0006612 | Apple colour | Polymorphic | T/C | 0.3557 |
| 128 | Pg_PC11_KASP115 | HEXgggtggccggactcttgT  FAMgggtggccggactcttgG  tcatcacctgctaagaccca | comp12124_c0_seq2/ scaffold0001102 | gibberellin receptor GID1B-like | P:GO:0048444; P:GO:0008152; P:GO:0009939; F:GO:0016787; P:GO:0010476 | Purple local | Polymorphic | A/C | 0.3398 |
| 129 | Pg_PC11_KASP130 | HEXtcccacaaaatacagaggatatacG  FAMtcccacaaaatacagaggatatacT  tgtaggttcccgtcatcatct | comp18472_c0_seq3/ scaffold0000401 | polygalacturonase ADPG2 | P:GO:0005975; F:GO:0016829; F:GO:0004650; P:GO:0016310; P:GO:0071555; C:GO:0005576; C:GO:0005774; F:GO:0016301 | Apple colour | Non-polymorphic | G/T | - |
